# Slow magnetic relaxation in distorted tetrahedral Dy(iii) aryloxide complexes†

**DOI:** 10.1039/d1cc02707g

**Published:** 2021-08-12

**Authors:** Vijay S. Parmar, Gemma K. Gransbury, George F. S. Whitehead, David P. Mills, Richard E. P. Winpenny

**Affiliations:** Department of Chemistry, School of Natural Sciences, The University of Manchester, Oxford Road Manchester M13 9PL UK david.mills@manchester.ac.uk

## Abstract

Three distorted tetrahedral Dy(iii) aryloxide complexes, [Na(THF)_6_][Dy(OAr^Ad_2_*t*Bu^)_2_Cl_2_] (1) (OAr^Ad_2_*t*Bu^ = OC_6_H_2_Adamantyl_2_-2,6-^*t*^Bu-4) and [Na(THF)_6_][Dy(OMes*)_3_X] (X = Cl, 2; BH_4_, 3), (OMes* = OC_6_H_2_^*t*^Bu_3_-2,4,6) exhibit easy axis magnetic anisotropy and slow magnetic relaxation at zero field, with relaxation rates 1 < 2 < 3.

## Abstract 2

Magnetostructural correlations: The magnetic relaxation dynamics of three distorted tetrahedral dysprosium aryloxide complexes are shown to vary according to their local coordination environments.

## Graphical abstract

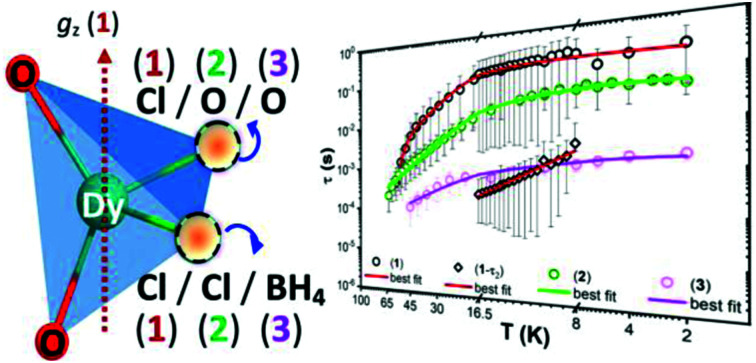

Single molecule magnets (SMMs) exhibit slow relaxation of magnetisation at the molecular level,^1,2^ and could be utilised in high density data storage, molecular spintronics, and quantum computing applications.^3^ SMMs containing a low coordination number (CN) Dy(iii) centre with axial crystal fields (CF) have their most magnetic *m*_J_ states stabilised, enhancing the energy barrier to magnetic reversal (*U*_eff_).^4,5^ Weakly coordinating ligands (or no ligands) in the equatorial plane can reduce the transverse CF components that are responsible for relaxation *via* quantum tunnelling of magnetisation (QTM).^1,2,6^ Magnetic relaxation in SMMs involves multiple pathways; it is necessary to improve our understanding of these processes in order to rationally target SMMs with longer relaxation times.^2^

The relaxation dynamics and magnetic anisotropy of Dy SMMs can be tuned by modulating the crystal field (CF) about the Dy centre.^6^ Bulky alkoxide and aryloxide ligands have proven useful in synthesising low-coordinate Dy SMMs with appropriate CF requirements.^5^ In recent years, axial CN7 Dy alkoxide SMMs with pentagonal bipyramidal geometries have been studied extensively.^7–10^ Only a handful of Dy alkoxide or aryloxide SMMs with CN < 6 have been reported to date.^11–14^ The only previous example of a CN4 Dy alkoxide SMM, [Dy(NPh_2_)(OCPh_3_)(μ-OCPh_3_)_2_Li(THF)], was disclosed by Yu *et al.* in 2016;^12^ conversely, there are a few CN4 lanthanide (Ln) amide SMMs; namely [Li(THF)_4_][Er{N(SiMe_3_)_2_}_3_Cl],^15^ [Ln{N(SiMe_3_)_2_}_3_(μ-Cl)Li(THF)_3_] (Ln = Dy, Er)^16^ and [K(DME)_3_][Ar^N^DyCl_2_] (Ar^N^ = (C_6_H_4_{(2,6-^i^PrC_6_H_3_)NC_6_H_4_}_2_)).^17^ Here, we report the synthesis of three CN4 Dy aryloxide complexes, [Na(THF)_6_][Dy(OAr^Ad_2_*t*Bu^)_2_Cl_2_] (**1**) (OAr^Ad_2_*t*Bu^ = OC_6_H_2_Ad_2_-2,6-^*t*^Bu-4) and [Na(THF)_6_] [Dy(OMes*)_3_X] (X = Cl (**2**), BH_4_ (**3**); OMes* = OC_6_H_2_^*t*^Bu_3_-2,4,6), which show slow magnetic relaxation in the absence of an external magnetic field; the relaxation dynamics of **1–3** vary according to their local coordination environments.

Complexes **1–3** were prepared by salt metathesis reactions of the sodium salt of the respective aryloxide ligand and anhydrous DyX_3_ (X = Cl for **1** and **2** and BH_4_ for **3**) in THF (Scheme 1); work-up and recrystallisation gave all complexes in *ca.* 40% yield. The diamagnetic Y(iii) analogue, **1-Y**, and a 5% doped sample, **5%Dy@1-Y**, were synthesised by analogous methods to perform complementary NMR spectroscopy and magnetic dilution experiments, respectively. The elemental analysis, and NMR and IR spectra of all complexes performed for characterisation purposes are compiled in the ESI.†

**Scheme 1:**
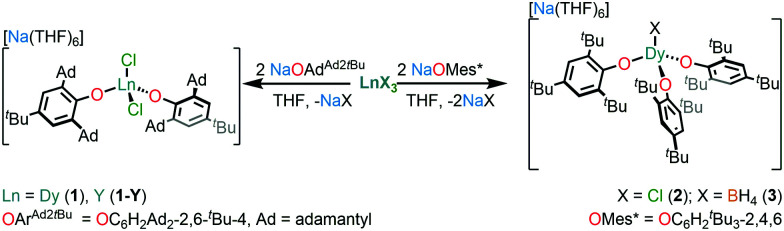
Synthesis of **1**, **1-Y**, **2** and **3**.

The solid state structures of **1–3** and **1-Y** were determined by single crystal XRD (see Fig. 1 for depictions of **1–3** and ESI† Fig. S8 and Table S1 for **1-Y** and crystallographic data). All structures contain octahedral [Na(THF)_6_]^+^ cations with unremarkable metrical parameters. The Ln(iii) centres in the anions exhibit distorted tetrahedral geometries, as confirmed by Shape2.0^18^ (Table S5, Fig. S11, ESI† and Fig. 2) and an angular index, *τ*_4_ = [[360 − (*a* + *b*)]/141] (*a* and *b* are the two largest angles about a four-coordinate metal centre);^19^*τ*_4_ values are 0.84, 0.89, 0.9 and 0.85 for **1**, **2**, **3** and **1-Y**, respectively. Complexes **1** and **1-Y** contain two OAr^Ad_2_*t*Bu^ and two Cl anions, whereas **2** and **3** each contain three OMes*, with the fourth ligand being Cl in **2** and the *pseudo*-halide BH_4_ in **3**.

**Fig. 1:**
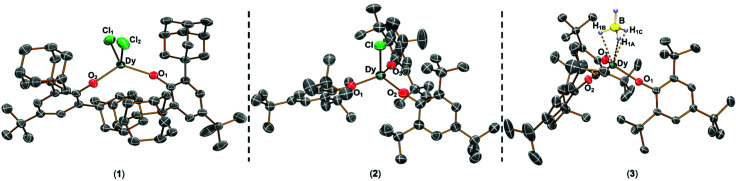
Molecular structures of **1–3** from single crystal XRD at 150 K with thermal ellipsoids drawn at 40% probability level (Dy teal, Cl green, O red, B yellow, C grey, H pale blue). H atoms (apart from those on BH_4_), counter-ions and lattice solvent are omitted for clarity. A κ^3^-binding mode is assigned for **3** from modelling the single crystal XRD data. We were not able to verify if this binding mode is representative of the bulk sample by IR spectroscopy as the diagnostic B–H stretching frequency region was not clear-cut.^20^

**Fig. 2:**
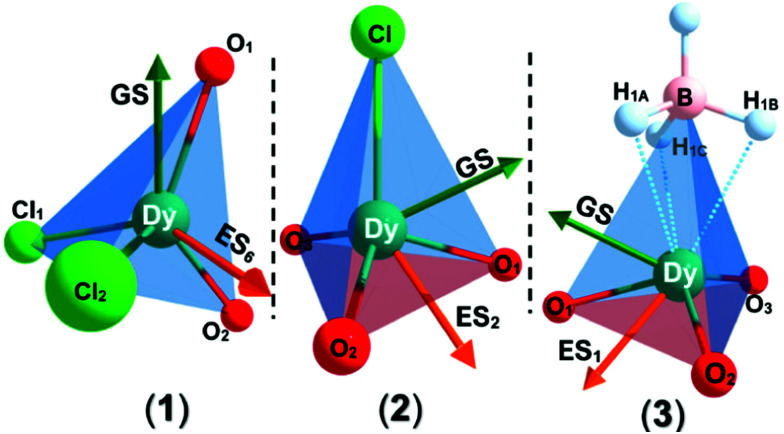
Local coordination environments of **1–3** (Dy teal, Cl green, O red, B pink, H pale blue). Arrows represent CASSCF-SO calculated magnetic axis (*g*_*z*_ vector), GS (dark green), and selected ES (orange) with significant angles of deviation of the *g*_*z*_ vector from the GS.

The Dy–O_Ar_ distances range from 2.083(2)–2.103(2) Å, 2.091(6)–2.132(5) Å and 2.109(2)–2.144(2) Å in **1**, **2** and **3**, respectively. The Dy–X distances are 2.549(1) Å for **1**, 2.553(2) Å for **2**, and 2.537(4) Å for **3**. As expected from steric arguments, the Dy–O_Ar_ distances are longer in the tris-aryloxide complexes **2** and **3** than in **1**. The largest O_Ar_–Dy–O_Ar_ angles in **1**, **2** and **3** are 122.37(8), 123.2(2) and 122.76(8)°, respectively. The nearest respective intermolecular Dy···Dy distances are 11.33 Å, 10.764 Å, and 10.747 Å in **1**, **2** and **3** (see Fig. S12–S15, ESI† for depictions of crystal packing). The only structurally authenticated CN4 Dy alkoxide SMM reported previously, [Dy(NPh_2_)(OCPh_3_)(μ-OCPh_3_)_2_Li(THF)], exhibits a distorted tetrahedral geometry, with three OCPh_3_ and one NPh_2_ coordinated to the Dy centre and two of the alkoxides bridging to a Li^+^ cation, showing shorter Dy–O bond distances (range 2.068(3)–2.273(4) Å) than in **1–3**.^12^

Static (direct current, dc) magnetic measurements were performed on polycrystalline samples of **1–3** immobilised in eicosane under a 1000 Oe external magnetic field between 2–300 K. At 300 K, molar *χ*_m_*T* values of 13.78, 14.42 and 14.28 cm^3^ mol^−1^ were observed for **1**, **2** and **3**, respectively (Fig. S20–S22, ESI†), which are within the range of calculated and reported values for mononuclear Dy(iii) complexes (Dy(iii) free ion ^6^H_15/2_, *χ*_m_*T* = 14.17 cm^3^ mol^−1^).^11,21,22^ The magnetisation at 2 K and 7 T saturates at *M*_sat_ = 4.67, 5.35, 5.36 and 4.64 N_A_μ_B_ for **1**, **2**, **3** and **5%Dy@1-Y** (when normalised per mole of Dy complex), respectively (Fig. S23–S26, ESI†). Magnetisation *vs.* Field traces also show the onset of slow dynamics at lower field and temperatures.

The ac magnetic susceptibility of **1–3** was measured. Peaks in the out of phase susceptibility (*χ*′′) between 2–56 K for **1**, 2–63 K for **2** and 2–45 K for **3** at various frequencies and zero external field show slow magnetic relaxation (Fig. S27–S29, ESI†). For **1**, a secondary peak in the intermediate temperature range (8–17 K), indicates two simultaneous relaxation processes (Fig. S33, ESI†); between 2–6 K, the two peaks overlap and are unresolved, whilst between 17–56 K the second peak shifts to outside the instrument's frequency limits. Consequently, the Cole–Cole isotherms (*χ*′ *vs. χ*′′) for **1** do not obey a generalised Debye (GD) model from 2–56 K, thus the high frequency ac data was omitted for fitting with the GD model between 2–8 K. The data between 17–56 K was fitted at all frequencies with the GD model, whilst the extended Debye (ED) model for two simultaneous relaxation processes was used to extract the magnetic relaxation times (*t*) and their associated distributions as a function of temperature from 8–16.5 K (Fig. S30 and S33, ESI†). *χ*′′ peaks were observed up to 63 K and 45 K for **2** and **3**, respectively, and were fitted with the GD model to extract their *τ* and *α* values (Fig. 3 and Fig. S31, S32, ESI†). The extracted α values range from 0.06–0.36 and 0.05–0.29 for **2** and **3**, respectively (Fig. S35–S37 and Tables S10–S13, ESI†). The extracted relaxation times from the simultaneous fitting of the temperature and frequency dependence of *χ*′ and *χ*′′ for **1** are greater than for **2** and **3**. The *α* parameters for **1–3** are large, representing a wide distribution of relaxation times (*τ*). The large errors on *τ* (Fig. 3), come from the log-normal distribution model which includes the *α* values.^23^

**Fig. 3:**
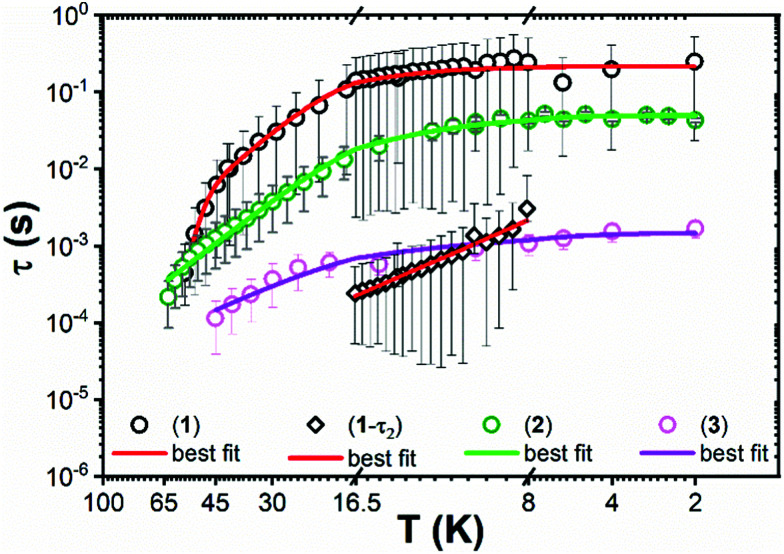
Temperature dependence (*K*) of relaxation time (*τ*) from 2–56 K for **1**, 2–63 K for **2** and 2–45 K for **3**. Circles represent primary relaxation channel and diamonds show secondary relaxation channel from 8–16.5 K in **1**. Error bars represent one standard deviation in the log-normal distributions of *τ* incorporating *α* parameters.^23^ Solid lines represent the best fits.

In the absence of an external dc field, magnetic relaxation occurs mainly *via* a combination of Orbach, Raman, and QTM relaxation mechanisms as follows:1
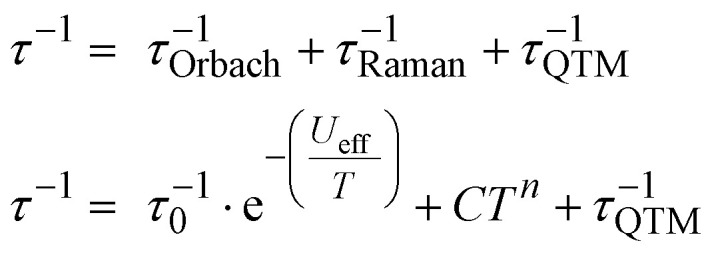


The temperature-dependent *τ* values (*τ vs. T*^−1^ curve) for **1–3** were parameterised with the logarithmic form of eqn (1) to extract the relaxation parameters for individual relaxation mechanisms (Table 1). Multiple attempts of fitting the temperature dependent relaxation rates were performed both with and without including the Orbach process; the best fit and meaningful parameters are presented here, whilst other fits are compiled in the ESI† (Fig. S38 and Table S14).

**Table 1:** *U*_eff_, *τ*_0_, *C*, *n* and *τ*_QTM_ parameters generated from the fit of the relaxation time-temperature dependence for **1–3**; parameters are given with their one-Sigma ESDs (±), subscripts and superscripts

Parameters	**1**	**1-** *τ*_2_	**2**	**3**
*τ*_0_ (10^−11^ s)	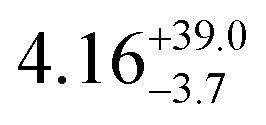			
*U*_eff_ (K)	916 ± 126			
*C* (10^−3^ s^−1^ K^−*n*^)	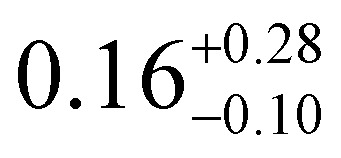	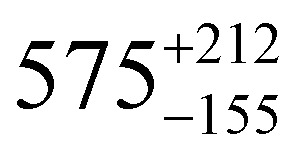	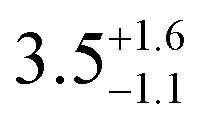	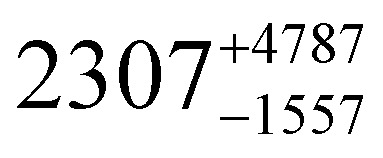
*n*	3.56 ± 0.29	3.19 ± 0.12	3.27 ± 0.10	2.08 ± 0.32
*τ*_QTM_ (10^−3^ s)	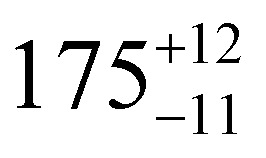		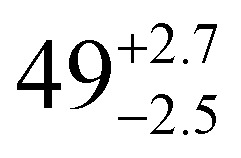	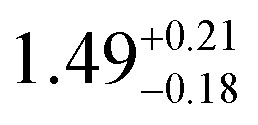

For **2** and **3** the Orbach region is not seen over a wide enough temperature range to include these relaxation rates in the fitting presented here, but it is clear that the observed relaxation rates trend as **1** < **2** < **3** (Fig. 3).

To probe the electronic structures of **1**, **2** and **3**, CASSCF-SO (complete active space self-consistent field spin–orbit) calculations were performed on Molcas 8^24^ using coordinates from single crystal XRD (see ESI† for details). The calculated principal magnetic axis for **1** aligns with the two aryloxides, whilst it is not aligned with any pair of aryloxides in **2** and **3** (Fig. 2). Complex **1** shows an axial ligand field with *g*_*x*_ = *g*_*y*_ = 0 and *g*_*z*_ = 19.72; the ground CF state is defined by 〈*m*_J_ ≈ 97%| ± 15/2〉 + 〈3%| ± 11/2〉. The *g* factors and ground *m*_J_ state compositions for **2** are *g*_*x*_ = *g*_*y*_ = 0.01, *g*_*z*_ = 19.74, 〈*m*_J_ ≈ 97.5%| ± 15/2〉 + 〈2.4%| ± 11/2〉, and for **3** are *g*_*x*_ = 0.02, *g*_*y*_ = 0.03, *g*_*z*_ = 19.70, 〈*m*_J_ ≈ 97%| ± 15/2〉 + 〈3%| ± 11/2〉 (Table S6, ESI†). The subsequent ES_1_ (Excited State 1) and ES_2_ are at 200 and 366 cm^−1^ for **1**, 178 and 274 cm^−1^ for **2** and 139 and 172 cm^−1^ for **3**, with higher mixing of the *m*_J_ levels occurring above this energy. The QTM rates were calculated at each *m*_J_ level within the thermally-assisted QTM (TA-QTM) frameworks^25^ to show significant QTM probabilities on all *m*_J_ levels; the low-lying state QTM rates are 7.1 s^−1^ (GS) and 2.3 × 10^3^ s^−1^ (ES_1_) for **1**, 8.8 × 10^2^ s^−1^ (GS) and 6.1 × 10^6^ s^−1^ (ES_1_) for **2** and 6.4 × 10^3^ s^−1^ (GS) and 4.5 × 10^8^ s^−1^ (ES_1_) for **3** (Table S8, ESI†). The calculated QTM rates at GS and ES1 are lowest for **1** and highest for **3**, indicating an increase in QTM from **1** < **2** < **3**.

The ground and excited states for **1** follow the order *m*_J_ = ±15/2 → ±13/2 → ±11/2; these states contain fractional mixtures of other *m*_J_ states. The calculated transition probabilities for **1** become significant around ES3 (735 K) and ES4 (905 K) (Tables S6 and S8, ESI†). Accounting for *m*_J_ state mixing and increased transition probabilities gives a calculated *U*_eff_ of *ca.* 700–900 K for **1** (Fig. S16, ESI†).

The highly mixed subsequent low-lying *m*_J_ states for **2** and **3** indicate less blocking of relaxation than in **1**; the GS-ES_1_ gap trends from highest to lowest in **1–3** (Fig. S19, ESI†). The calculated LoProp^26^ charges for the first coordination spheres of **1–3** are tabulated in Table S9 (ESI†). The calculated charges on the Dy, O_average_, and (*pseudo*-) halide centres are 2.448, −0.842 and −0.952 for **1**, 2.488, −0.958 and −0.842 for **2**, and 2.520, −0.952 and −0.901 for **3**. The Dy centre becomes more positively charged from **1** to **3**; the average negative O_Ar_ charge is invariant from **2** to **3**, whilst it decreases significantly in **1** due to the presence of two competing Cl donors *vs.* one (*pseudo*-) halide donor in **2** and **3**.

The magnetic relaxation parameters reported for **1** contrast with those reported for [Dy(NPh_2_)(OCPh_3_)(μ-OCPh_3_)_2_Li(THF)] (*U*_eff_ = 35.9 K, *τ*_0_ = 1.22 × 10^−8^ s, *C* = 0.59 K^−n^ s^−1^ and *n* = 6.53);^12^ for the former the principal magnetic axis aligns with the two O-donors of the aryloxides, whereas for the latter a single alkoxide is colinear and two alkoxides are transverse to the principal magnetic axis. The magnetic behaviour of **1** is more similar to the CN4 Dy SMM [K(DME)_3_][Ar^N^DyCl_2_] (Ar^N^ = (C_6_H_4_{(2,6-^i^PrC_6_H_3_)NC_6_H_4_}_2_)), which has a DyN_2_Cl_2_ core in a see-saw geometry and a *U*_eff_ of 1334 K, with QTM at low temperature restricting magnetic hysteresis at zero field.^17^ The second relaxation channel in **1** is strongly dependent on temperature and its relaxation rates are 100 times faster than the primary relaxation channel. The presence of multiple relaxation channels in Ln SMMs is not unusual,^27–29^ but identifying the contributions of the individual processes and their relation to the molecule and the CF is challenging due to the complex electronic structures of Ln.^7,28–30^ Each relaxation channel has its own unique combination of processes (Orbach, Raman, QTM or Direct), which can often provide multiple relaxation barriers; these can sometimes be assigned to lattice disorder giving a fraction of molecules with slightly different magnetocrystalline anisotropy, leading to varied dynamics.^30^

The magnetically diluted sample **5%Dy@1-Y** was studied to estimate the extent of dipolar field effects on QTM process and the two relaxation channels in **1**. The ZFC-FC plot for **5%Dy@1-Y** showed a peak in ZFC mode at 6 K in 500 and 1000 Oe field (Fig. S39, ESI†), which was absent in pure **1**. The *χ*′′ *vs. T* plots for **5%Dy@1-Y** show peaks from 9–52 K, and two relaxation peaks between 13–43 K. The ac data for **5%Dy@1-Y** is noisy, hence the fitting results were unreliable; the extracted *τ*, *τ*_2_, *α* and *α*_2_ parameters can be found in the ESI† (Table S16). The relaxation times for **5%Dy@1-Y** in the primary relaxation channel are similar (within errors) to **1** between 9–52 K, whilst *τ*_2_ in the secondary relaxation channel are two orders of magnitude slower than in **1**, suggesting a change in the Raman relaxation process of the second relaxation channel upon magnetic dilution (Fig. S44, ESI†). The magnetic relaxation rate in **5%Dy@1-Y** at *T* < 8 K, as measured by dc magnetisation decay (Fig. S43, ESI†), is a thousand times slower than in **1**, consistent with the ZFC-FC trace (Fig. S39 and S44, ESI†).

Despite the non-ideal arrangement of the ligands about Dy for favourable SMM properties, **1** exhibits easy-axis anisotropy and Ising-type SMM behaviour with a *m*_J_ ≈ ±15/2 ground state and a high *U*_eff_, which is consistent with the *ab initio* calculated results. Axial ligand fields and easy axis *m*_J_ ≈ ±15/2 ground states in distorted tetrahedral Dy(iii) SMMs are consistent with the literature.^31^ Upon introducing a third aryloxide in the distorted tetrahedral coordination environments of **2** and **3**, the calculated GS-ES_1_ gap decreases and a significant increment in the *m*_J_ mixing of the lowest-lying excited state is seen (Fig. S19, ESI†) which is consistent with the lack of an Orbach process in **2** and **3**. (Fig. 3). In contrast with these results, [Li(THF)_4_][Er{N(SiMe_3_)_2_}_3_Cl]^15^ and [Er{N(SiMe_3_)_2_}_3_(μ-Cl)Li(THF)_3_]^16^ show slow magnetic relaxation at zero field, indicating that ErL_3_Cl local configurations are a relatively favourable geometry for Er(iii) SMMs; [Dy{N(SiMe_3_)_2_}_3_(μ-Cl)Li(THF)_3_] shows diminished SMM performance, with an easy plane calculated ground state.^16^

In conclusion, the magnetic properties of three CN4 Dy complexes with distorted tetrahedral geometries were shown to vary due to their ligand substitution patterns. Complex **1** has a DyO_2_Cl_2_ local coordination environment with an axial ligand field and an easy axis magnetic ground state, to show a large *U*_eff_ of ≈ 900(100) K; in contrast **2** and **3** possess DyO_3_X local environments, and magnetic relaxation is faster in both.

We thank the University of Manchester for the President's Doctoral Scholarship for V. P. and access to the Computational Shared Facility, the European Research Council (ADG-786734 for R. E. P. W. and CoG-816268 for D. P. M. and G. K. G.) and the UK EPSRC (EP/P002560/1, EP/R011079/1) for funding, and the EPSRC National Electron Paramagnetic Resonance Service for access to the SQUID magnetometer (EP/S033181/1). Research data files supporting this publication are available from Figshare at DOI: 10.48420/14635464.

## Conflicts of interest

The authors declare no conflict of interest.

## Supplementary Material

CC-057-D1CC02707G-s001

CC-057-D1CC02707G-s002
