# Supplementary material for: Slow magnetic relaxation in distorted tetrahedral Dy(iii) aryloxide complexes
Source: Chem Commun (Camb). 2021 Aug 12;57(73):9208–11. doi: 10.1039/d1cc02707g (PMC8438701; doi:10.1039/d1cc02707g)
Supplement: CC-057-D1CC02707G-s001 [file CC-057-D1CC02707G-s001.pdf]

Electronic Supplementary Material (ESI) for ChemComm.
This journal is © The Royal Society of Chemistry 2021

Slow Magnetic Relaxation in Tetrahedral Dy(III) Aryloxide Complexes.

Vijay S. Parmar, Gemma K. Gransbury, George F. S. Whitehead David P. Mills*, Richard E. P. Winpenny*

Department of Chemistry, The University of Manchester, Manchester M13 9PL, UK.

1. General experimental procedures 2

2. Experimental synthesis 2

3. Crystallography 10

4. Computational details 20

5. Magnetism 28

6. References 53

1. General experimental procedures

All syntheses and manipulations were conducted under argon with the rigorous exclusion of oxygen and water using Schlenk line and glovebox techniques. THF and hexane were obtained from a central solvent purification facility (SPS drying columns), and diethyl ether was dried by refluxing over Na/K alloy and distillation. Hexane and diethyl ether were stored over potassium mirrors and THF was stored over 4 Å molecular sieves. All solvents were degassed before use. For NMR spectroscopy, THF-d_8 was dried by refluxing over potassium, then vacuum transferred and degassed by three freeze-pump-thaw cycles before use. Anhydrous $LnCl_3$ (Ln = Y or Dy) were purchased from Alfa Aesar and were used as received. Mes*OH (2,4,6-tris-*tert*-butylphenol) was purchased from Merck and dried *in vacuo* for six hours at room temperature with occasional gentle heating. $HOAr^{Ad2tBu}$[1] and NaOMes*[2] were prepared by literature procedures. ^{1}H (500 MHz) and $^{13}C\{^{1}H\}$ (100 MHz and 125 MHz) NMR spectra were obtained on an Avance III 400 MHz or 500 MHz spectrometers at 298 K. These were referenced to the solvent used or to external TMS (^{1}H). ATIR spectra were recorded as microcrystalline powders using a Bruker Tensor 27 ATR-Fourier Transform infrared (ATR-FTIR) spectrometer. Elemental analyses were performed by Mrs Anne Davies and Mr Martin Jennings at the Microanalysis Service in the Department of Chemistry at the University of Manchester, UK.

2. Experimental synthesis

$NaOAr^{Ad2tBu}\cdot Et_2O$: $NaOAr^{Ad2tBu}$ was prepared following a similar procedure to the synthesis of $KOAr^{Ad2tBu}$ in reference 3. A mixture of the white suspension of $Ar^{Ad2tBu}OH$ (4.19 g, 10.00 mmol) and $[Na\{N(SiMe_3)_2\}]$ (1.90 g, 12.00 mmol) was dissolved in 100 mL of diethyl ether at room temperature. After stirring overnight, a pale yellow solution

with thick white precipitate was produced. The supernatant was filtered off and the precipitate was washed with diethyl ether (20 mL) and hexane (2 x 20 mL). The product was dried *in vacuo* for several hours; purity was assumed for subsequent reactions. Yield 70% (3.60 g, 7.00 mmol).

[Na(THF)$_6$][Y(OAr^{Ad2tBu})$_2$Cl] (1-Y)

A Schlenk was charged with YCl_3 (0.196 g; 1.00 mmol) and cooled to -78 ºC before adding 20 ml of THF; this mixture was allowed to warm to room temperature with stirring and was refluxed for 2 hours. The resultant slurry was added to NaOAr^{Ad2tBu}·Et_2O (1.00 g; 2.00 mmol) suspended in 15 mL THF at room temperature. The grey turbid reaction mixture was stirred at room temperature overnight. The reaction mixture was allowed to settle to give a clear, colourless supernatant and grey precipitate, which was expected to be NaCl. The supernatant was filtered to another Schlenk and concentrated to approximately 5-8 mL volume for crystallisation. Colourless crystals were formed at -25 ^{o}C after 48 hrs; these were isolated and dried in vacuo to afford a white crystalline solid, yield 37% (0.530 g, 0.37 mmol). The NMR data for **1-Y** in THF-d_8 shows a small amount of decomposition to form Ar^{Ad2tBu}OH in the presence of a polar solvent, as shown by the salient peaks at 7.11, 5.70, 2.08, 2.19 and 1.29 in the ^{1}H NMR spectrum and peaks at 122.1, 33 and 35.9 in the ^{13}C-DEPT-Q NMR spectrum. The decomposition can also be visually observed when the white powder dissolved in THF rapidly gives a blue tint, which may arise from a possible phenoxide radical formation upon dissociation of **1-Y**. Complex **1-Y** and Ar^{Ad2tBu}OH have very similar solubility; they are only sparingly soluble in arenes; hence a well-resolved spectrum in non-polar solvent was not achieved and the ^{13}C NMR spectrum could not be interpreted. These observations with this ligand system are not

uncommon; Evans *et al.* recently reported similar behaviour whilst utilising the $Ar^{Ad2tBu}OH$ ligand.[4] The characteristic sharp O–H stretch (3600-3700 cm^{-1}) of $Ar^{Ad2tBu}OH$ was not observed in the infrared spectrum of **1-Y**. The resultant residual $Ar^{Ad2tBu}OH$ impurity as quantified by NMR analysis is ~15%. Elemental analysis calculated for $C_{84}H_{130}Cl_2NaO_8Y$: C, 69.54; H, 9.03 Found (%): C, 70.29; H, 8.54. ^1H NMR (500 MHz, THF-d_8) δ ppm 1.25 (s, 18 H), 1.29 (s, 4 H – $Ar^{Ad2tBu}OH$), 1.59 - 1.61 (br d, 12 H), 1.74 (br s, 8 H), 1.85 - 1.88 (br d, 15 H), 1.93 (br m, 10 H – $Ar^{Ad2tBu}OH$), 2.08 (br m, 3 H), 2.19 - 2.24 (br, m, 32 H – $Ar^{Ad2tBu}OH$ overlap), 3.60 (br m, 8 H) , 5.70 (s, 1 H – $Ar^{Ad2tBu}OH$), 6.95 (s, 4 H), 7.11 (s, 1 H – $Ar^{Ad2tBu}OH$). ^1H NMR (126 MHz, THF-d_8) δ = 25.6 - 26.2 (–C_{THF}), 31.0 (–Ad-*C*H), 38.6 (–Ad-*C*H_2-distal to Ph ring), 38.8 (–C(*C*H_3)$_3$), 39.4 (–Ad-*C*), 44.4 (–Ad-*C*H_2-closer to Ph ring), 67.6 - 68.4 (–C_{THF}), 122.7 (–C_{meta}), 137.2 (–C_{para}), 137.5 (–C_{ortho}), 153.7 (–O*C*). FTIR (ATR, microcrystalline) ῦ: 2900 (w), 2846 (w), 1442 (w), 1428 (w), 1391 (w), 1358 (w), 1342 (w), 1311 (w), 1278 (w), 1260 (w), 1231 (w), 1202 (w), 1139 (w), 1102 (w), 1044 (w), 980 (w), 871 (w), 845 (w), 810 (w), 771 (w), 736 (w), 615 (w), 545 (w), 469 (w), 450 (w), 430 (w), 405 (w) cm^{-1}.

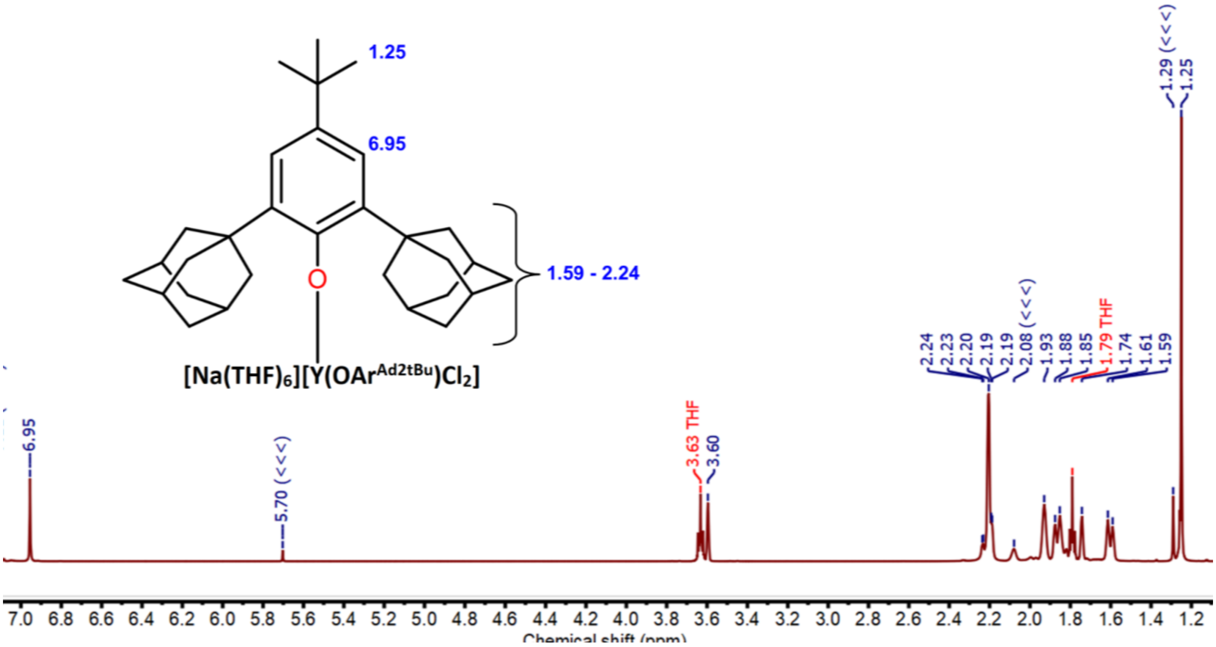

Figure S1. ^{1}H NMR spectrum of **1-Y** [Y(OAr^{Ad2tBu})$_2$Cl$_2$][Na(THF)$_6$] in THF-d_8. Residual HOAr^{Ad2tBu} impurities are marked with (<<<).

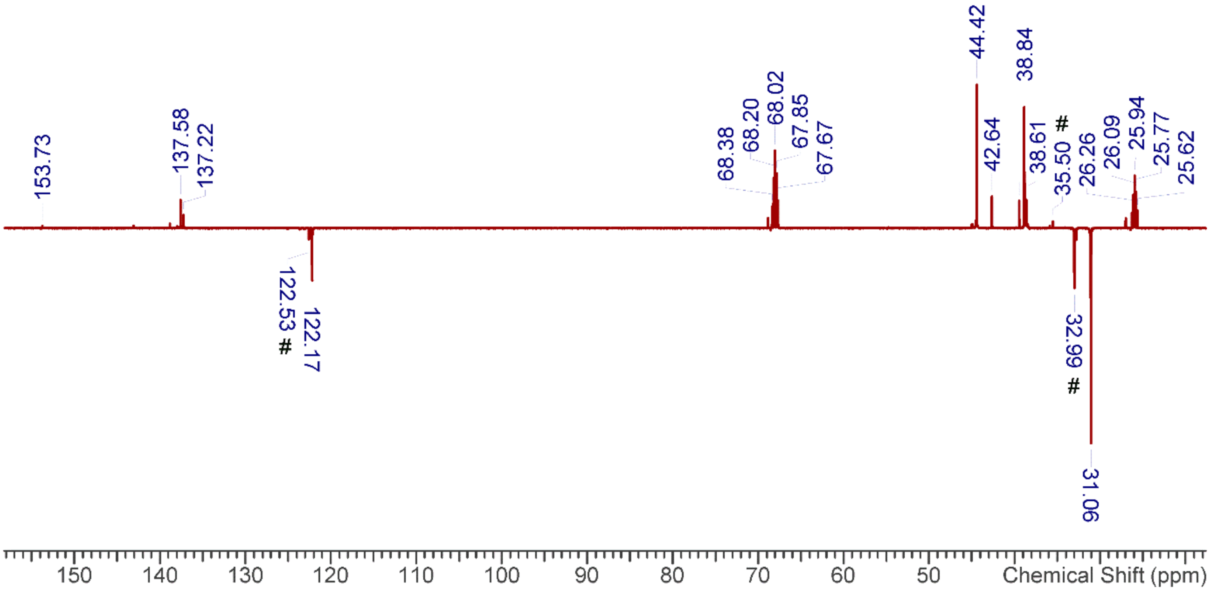

Figure S2. ^{13}C DEPT-Q NMR spectrum of **1-Y** [Y(OAr^{Ad2tBu})$_2$Cl$_2$][Na(THF)$_6$] in THF-d_8. Residual HOAr^{Ad2tBu} impurities are marked with (#).

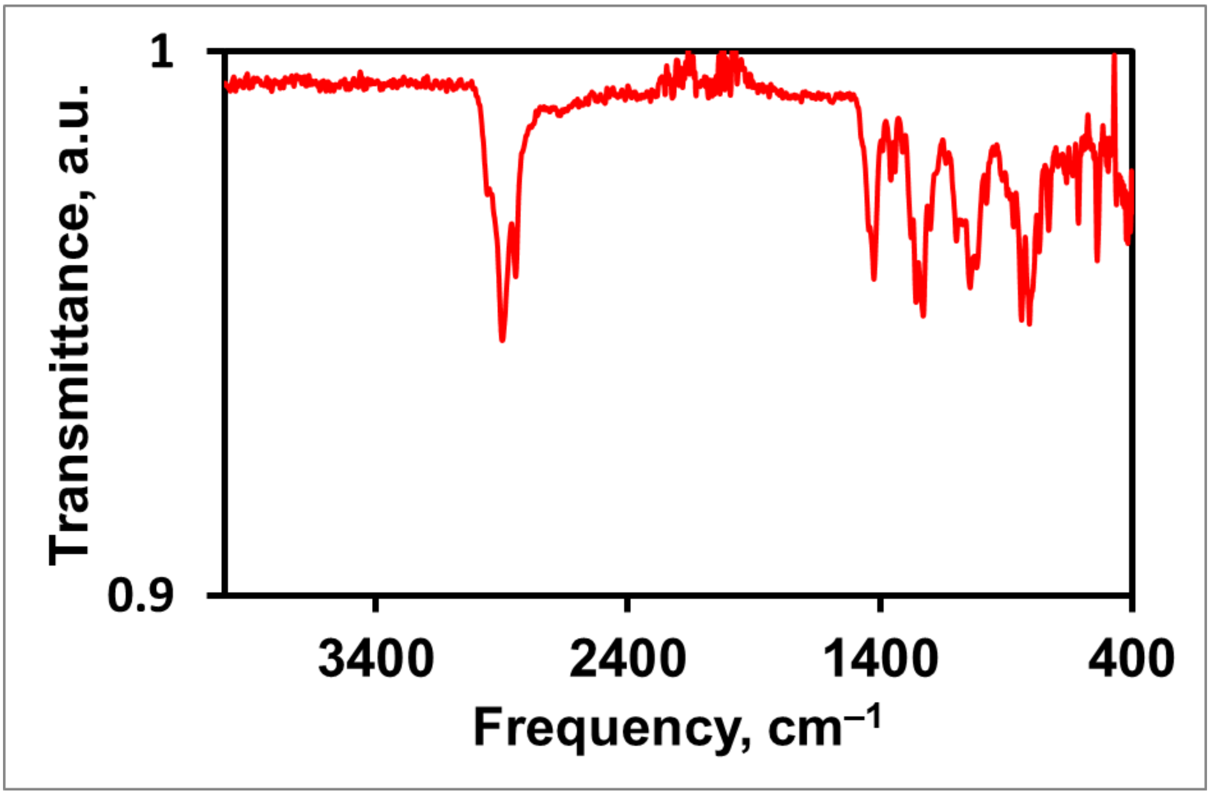

Figure S3. ATR-IR spectrum of **1-Y**, 400-4000 cm^{-1}.

[Na(THF)$_6$][Dy(OAr^{Ad2tBu})$_2$Cl$_2$] (1):

Complex **1** was synthesised analogously to **1-Y** with $DyCl_3$ (0.268 g; 1.00 mmol). Colourless crystals were formed at -25 °C after 48 hours; yield 30% (0.447 g, 0.30 mmol). The crystals were dried in vacuo to afford a white crystalline solid, which elemental analysis indicates is partially desolvated **1**. Anal. Calcd for $C_{84}H_{130}Cl_2DyNaO_8$: C, 69.91; H, 8.60 Found (%): C, 68.29; H, 8.62. Elemental analysis results show lower carbon values than predicted, which we attribute to carbide formation from incomplete combustion. FTIR (ATR, microcrystalline) ũ: 2960 (w), 2959 (w), 2900 (w), 2846 (w), 1447 (w), 1426 (w), 1358 (w), 1342 (w), 1278 (w), 1260 (w), 1231 (w), 1202 (w), 1100 (w), 1044 (w), 1019 (w), 980 (w), 871 (w), 841 (w), 810 (w), 771 (w), 734 (w), 615 (w), 541 (w), 464 (w), 425 (w), 419 (w), 409 (w) cm^{-1}.

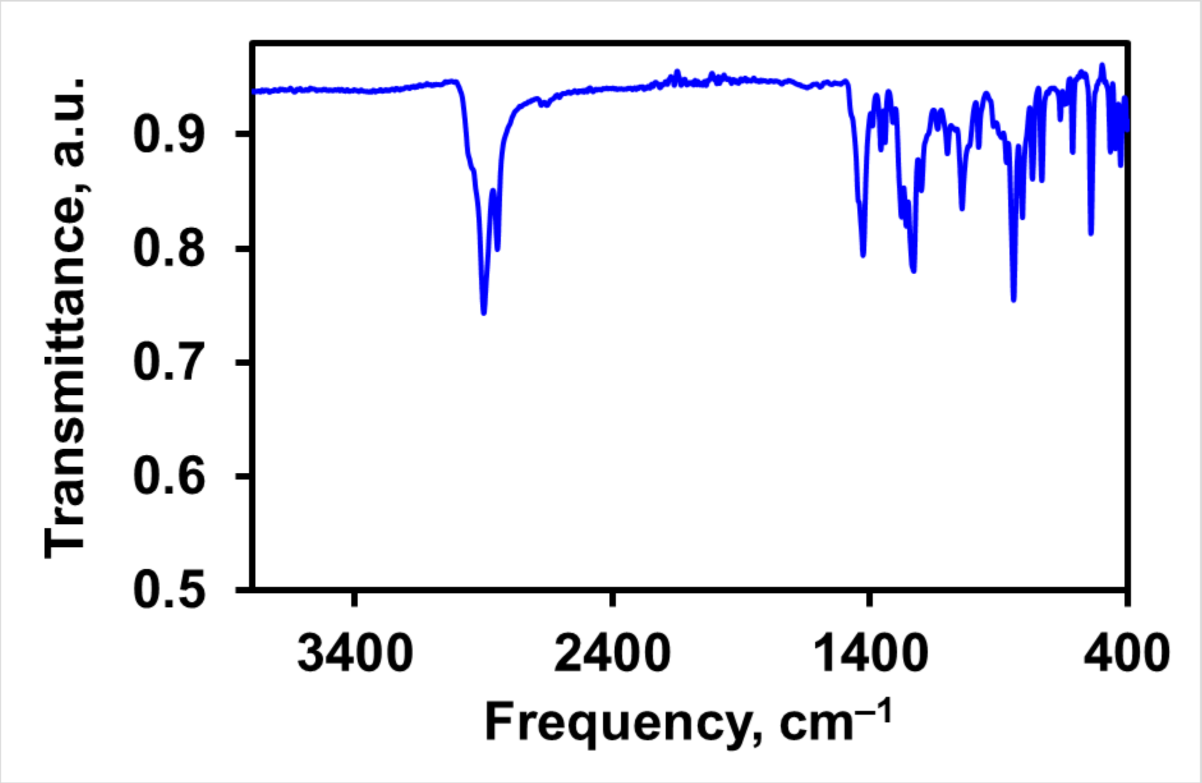

Figure S4. ATR-IR spectrum of **1**, 400-4000 cm^{-1}.

[Na(THF)$_6$][Dy(OMes*)$_3$Cl] (2)

A Schlenk charged with $DyCl_3$ (0.536 g; 2.00 mmol) and NaOMes* (1.434 g; 4.00 mmol) was cooled to -78 ºC before adding 30 ml of THF. The grey turbid reaction mixture was refluxed for 2 hours. The reaction mixture was allowed to settle to give colourless supernatant and an off-white precipitate, which was expected to be NaCl. The supernatant was filtered to another Schlenk and concentrated to approximately 10 mL volume for crystallisation. Colourless crystals were formed at -25 ºC after 48 hrs, yield 36% (1.024 g, 0.71 mmol). The crystals were dried in vacuo to afford a white crystalline solid, which elemental analysis indicates is desolvated **2**. Anal. Calcd for $C_{78}H_{135}ClDyNaO_9$: C, 65.16; H, 9.46, Found (%): C, 62.31, 9.10. Elemental analysis results show lower carbon values than predicted, which we attribute to carbide formation from incomplete combustion. FTIR (ATR, microcrystalline) ũ: 2949 (w), 2904 (w), 2869 (w), 1476 (w), 1455 (w), 1418 (w), 1385 (w), 1358 (w), 1254 (w), 1237 (w), 1198 (w), 1118 (w), 1048 (w), 1015 (w), 913 (w), 890 (w), 876 (w), 834 (w), 818 (w), 781 (w), 744 (w), 643 (w), 532 (w), 456 (w), 400 (w). cm^{-1}.

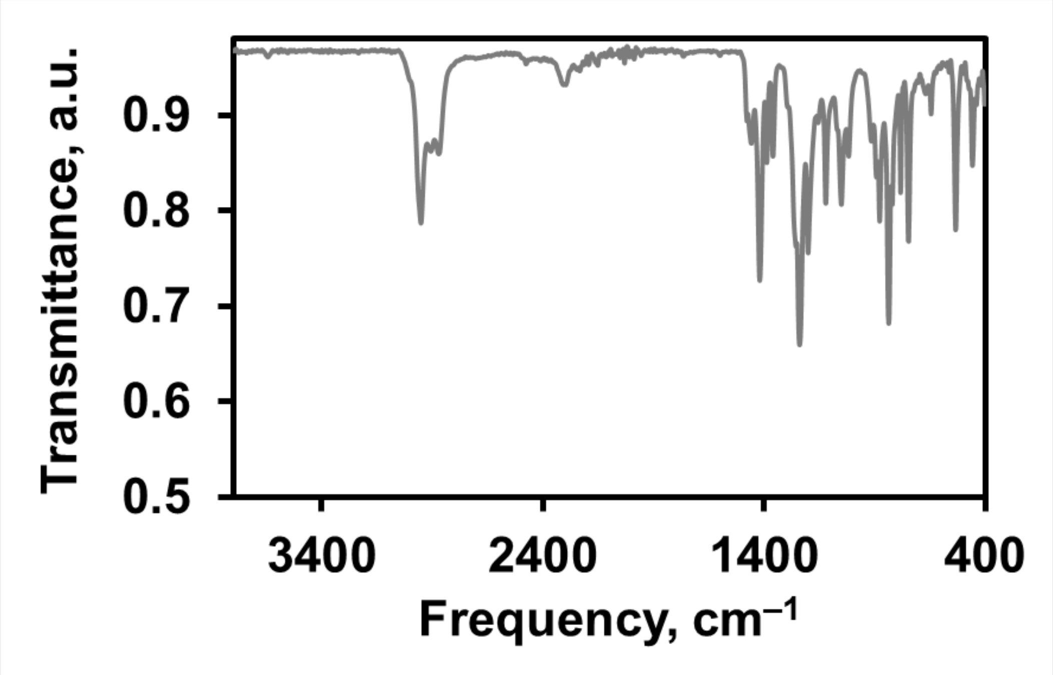

Figure S5. ATR-IR spectrum of **2**, 400-4000 cm^{-1}.

[Na(THF)$_6$][Dy(OMes*)$_3$(BH$_4$)] (3)

A Schlenk was charged with Dy(BH$_4$)$_3$THF$_{3.5}$ (0.666 g; 1.00 mmol) and NaOMes* (0.717 g; 2.00 mmol) and cooled to -78 ºC before adding 30 mL of THF. The white turbid reaction mixture was stirred at room temperature for 48 hours. The reaction mixture was allowed to settle to give colourless supernatant and a minimal amount of white precipitate, which was expected to be NaBH$_4$. The supernatant was filtered to another Schlenk and concentrated to approximately 10 mL volume, and left at 4 ^{o}C overnight. Some more white precipitate was observed; the colourless supernatant was filtered into another Schlenk, which was stored at -25 ^{o}C for crystallisation. Colourless crystals were formed at -25 ^{o}C after 48-96 hrs, yield 37% (0.523 g, 0.369 mmol). The crystals were dried in vacuo to afford a white crystalline solid, which elemental analysis indicates is desolvated **3**. Anal. Calcd for C$_{78}$H$_{139}$BDyNaO$_9$: C, 66.10; H, 9.89, Found (%): C, 59.58, 9.53. Elemental analysis results show lower carbon values than predicted, which we attribute to carbide formation from incomplete combustion. FTIR (ATR, microcrystalline) ũ: 2949 (w), 2904 (w), 2871 (w), 2470 (w), 2302 (w), 2219(w), 1476 (w), 1453 (w), 1414 (w), 1385 (w), 1358 (w), 1276 (w), 1260 (w), 1231 (w), 1198 (w), 1155 (w), 1118 (w), 1036 (w), 1015 (w), 913 (w), 890 (w), 876 (w), 830 (w), 818 (w), 781 (w), 744 (w), 672 (w), 641 (w), 528 (w), 452 (w), 436 (w) cm^{-1}.

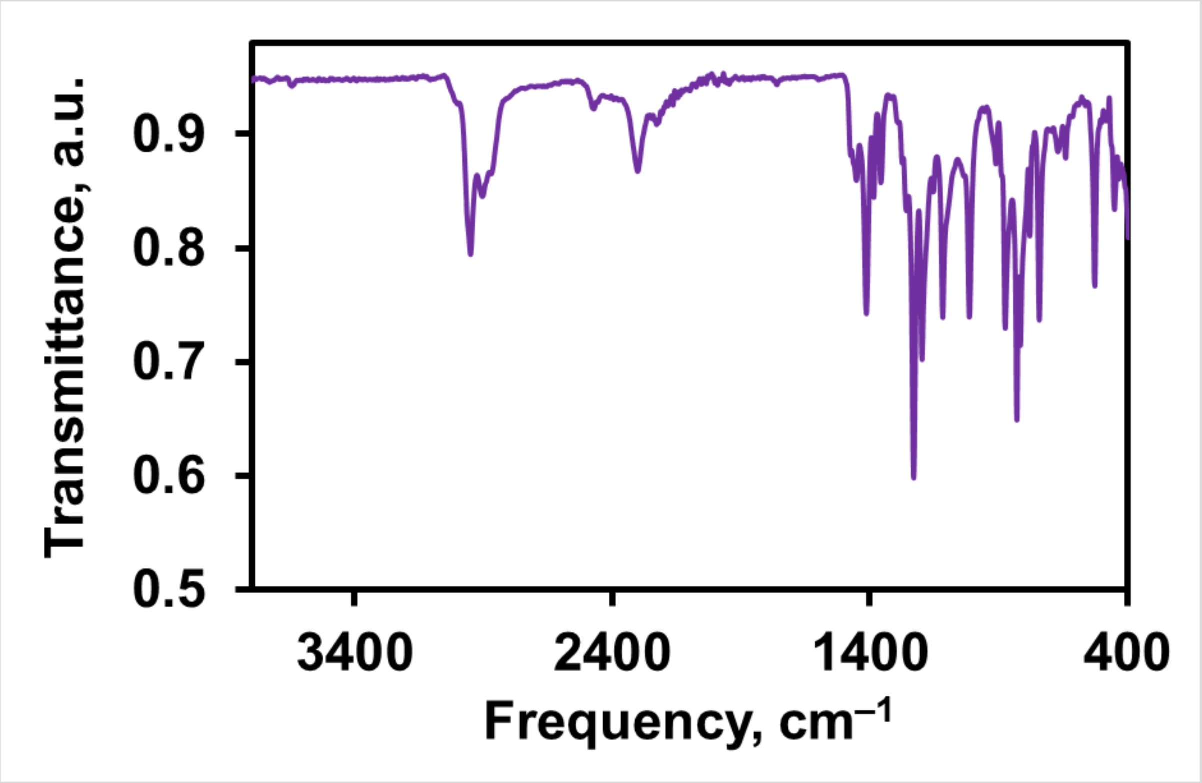

Figure S6. ATR-IR spectrum of **3**, 400-4000 cm^{-1}.

5%Dy@1-Y:

This doped sample was synthesised analogously to **1-Y** with $DyCl_3$ (0.0134 g; 0.05 mmol) and YCl_3 (0.186 g; 0.95 mmol). Colourless crystals were formed at -25 ^{o}C, yield 30% (0.450 g, 0.30 mmol).

3. Crystallography

The crystal data for complexes **1**, **1-Y**, **2** and **3** are compiled in Table S1. Crystals of **1**, **1-Y** and **3** were examined using a Rigaku XtalLAB AFC11 diffractometer with a CCD area detector and a graphite-monochromated Cu Kα (λ = 1.54178 Å). Crystals of **2** were examined using a Bruker Apex II diffractometer, equipped with a CCD area detector and a graphite-monochromated Cu Kα radiation (λ = 1.54178 Å). Intensities were integrated from data recorded on 1° frames by ω rotation. Cell parameters were refined from the observed positions of all strong reflections in each data set. A multi-scan absorption correction with a beam profile was applied.[5] The structures were solved using direct methods by SHELXS; the datasets were refined by full-matrix least-squares on all unique F^2 values.[6] CrysAlisPro[5] was used for control and integration, and SHELX[6,7] was employed through OLEX2[8] for structure solution and refinement. ORTEP-3[9] and POV-Ray[10] were employed for molecular graphics. CCDC 2085017-2085020 contain the supplementary crystal data for this article. Complex **5%Dy@1-Y** is isostructural to **1** and **1-Y** with unit cell parameters a = 11.3242(2), b = 46.4369(6), c = 16.9101(3); α = 90° β = 94.248(2)° γ = 90°. These data can be obtained free of charge from the Cambridge Crystallographic Data Centre via www.ccdc.cam.ac.uk/data_request/cif.

Table S1. Crystallographic data for **1**, **1-Y**, **2** and **3**.

	**1**	**1-Y**	**2**	**3**
Formula	$C_{96}H_{154}Cl_2DyNaO_{11}$	$C_{96}H_{154}Cl_2NaO_{11}Y$	$C_{86}H_{151}ClDyNaO_{11}$	$C_{86}H_{155}BDyNaO_{11}$
Fw, g mol^{-1}	1740.57	1666.98	1582.00	1561.39
Cryst size, mm	0.358 × 0.12 × 0.088	0.31 × 0.169 × 0.105	0.1 × 0.1 × 0.1	0.217 × 0.105 × 0.089
Crystal system	monoclinic	monoclinic	monoclinic	monoclinic
Space group	$P2_1/c$	$P2_1/c$	$P2_1/c$	$P2_1/c$
Collection Temperature (K)	150	100	150	150
a, (Å)	11.39679(13)	11.3031(4)	15.7770(15)	15.72802(17)
b, (Å)	46.8821(5)	46.2765(15)	19.5854(16)	19.6357(2)
c, (Å)	16.9513(2)	16.8926(7)	28.945(3)	28.9797(3)
α, (°)	90	90	90	90
β, (°)	94.0073(11)	94.260(4)	92.058(9)	92.6173(10)
γ, (°)	90	90	90	90
V, (Å^3)	9035.02(18)	8811.6(6)	8938.2(14)	8940.47(17)
Z	4	4	4	4
ρ_{calc} g cm^{-3}	1.280	1.257	1.176	1.160
μ, mm^{-1}	5.463	0.787	5.203	4.924
No. of reflections measured	50657	61122	37966	52900
No. of unique reflections, R_{int}	18249, 0.0470	17943, 0.0602	10044, 0.1291	18351, 0.0479
No. of reflections with $F^2 > 2s(F^2)$	14786	11942	6468	14731
Transmission coefficient range	0.612-1.000	0.821-1.000	0.15523-1.00000	0.471-0.735
R_1 $(I > 2\sigma(I))$	0.0463, 0.1267	0.0874, 0.1840	0.0744, 0.1723	0.0472, 0.1212
wR_2 (all data)	0.0578, 0.1354	0.1349, 0.1998	0.1132, 0.2056	0.0606, 0.1286
S^a	1.065	1.090	0.921	1.092
Parameters, Restraints	1370, 3979	1301, 2777	1196, 3208	1308, 4045
Max./min. difference map, e Å^{-3}	0.586, -0.735	0.525, -0.451	1.083, -0.949	0.898, -1.559

[a]Conventional $R = \Sigma||F_o| - |F_c||/\Sigma|F_o|$; $R_w = [\Sigma w(F_o^2 - F_c^2)^2/\Sigma w(F_o^2)^2]^{1/2}$; $S = [\Sigma w(F_o^2 - F_c^2)^2/\text{no. data} - \text{no. params})]^{1/2}$ for all data.

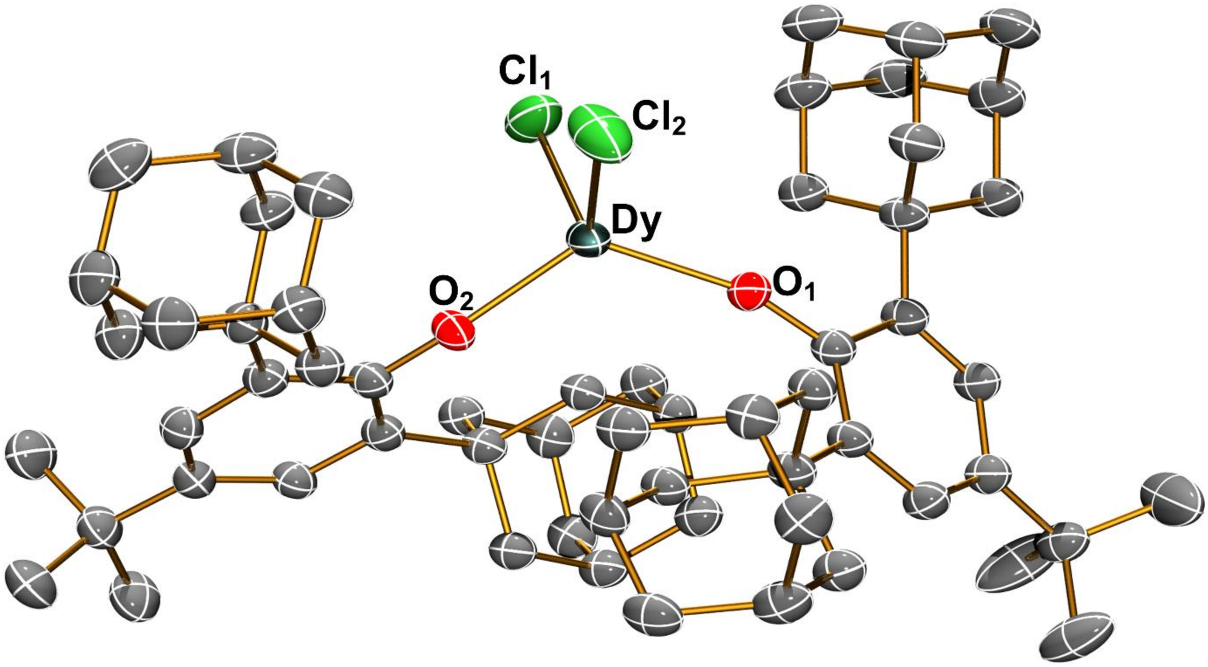

Figure S7. View of the molecular structure of $[Dy(OAr^{Ad2tBu})_2Cl_2]^-$ (**1**) from single crystal XRD at 150 K with thermal ellipsoids drawn at 40% probability level (Dy teal, Cl green, O red, C grey). H atoms, counter ion and lattice solvents are omitted for clarity.

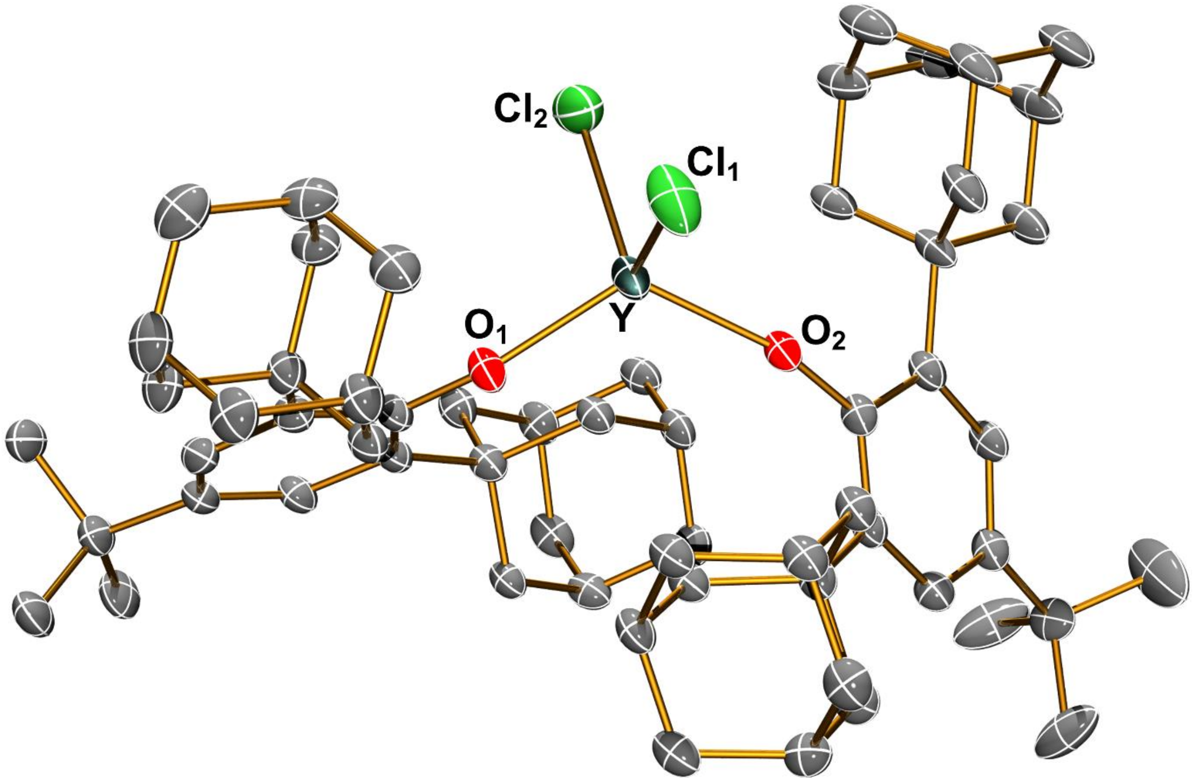

Figure S8. View of the molecular structure of $[Y(OAr^{Ad2tBu})_2Cl_2]^-$ (**1-Y**) from single crystal XRD at 150 K with thermal ellipsoids drawn at 40% probability level (Y teal, Cl green, O red, C grey). H atoms, counter ion and lattice solvents are omitted for clarity.

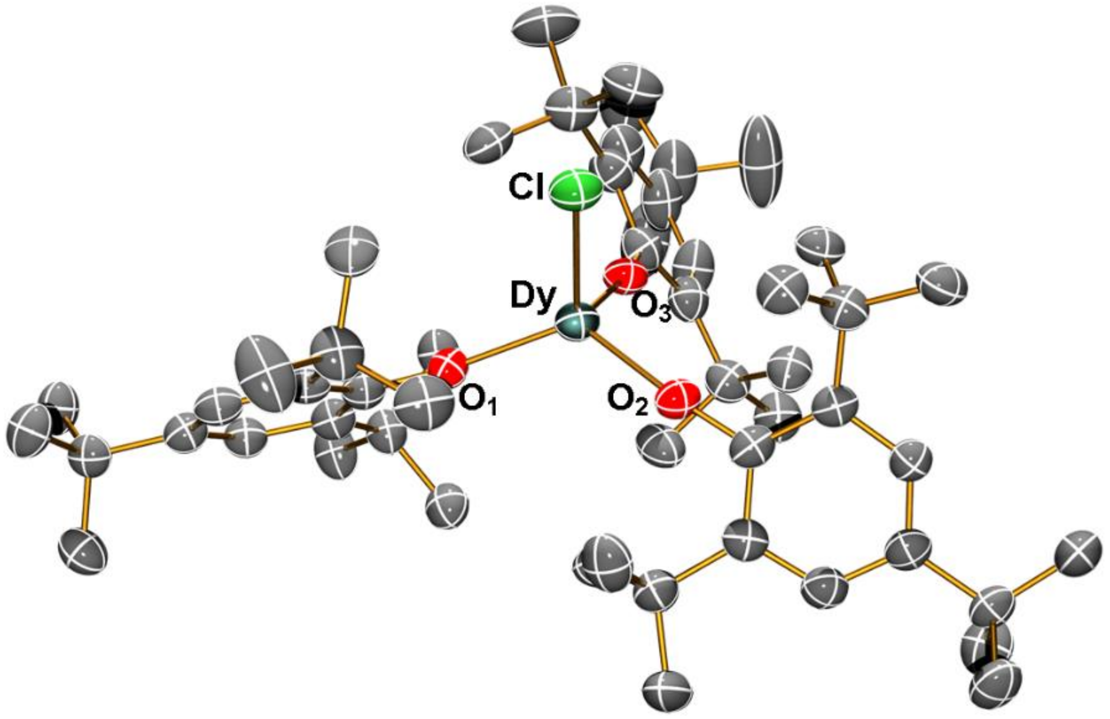

Figure S9. View of the molecular structure of $[Dy(OMes^*)_3Cl]^-$ (**2**) from single crystal XRD at 150 K with thermal ellipsoids drawn at 40% probability level (Dy teal, Cl green, O red, C grey). H atoms, counter ion and lattice solvents are omitted for clarity.

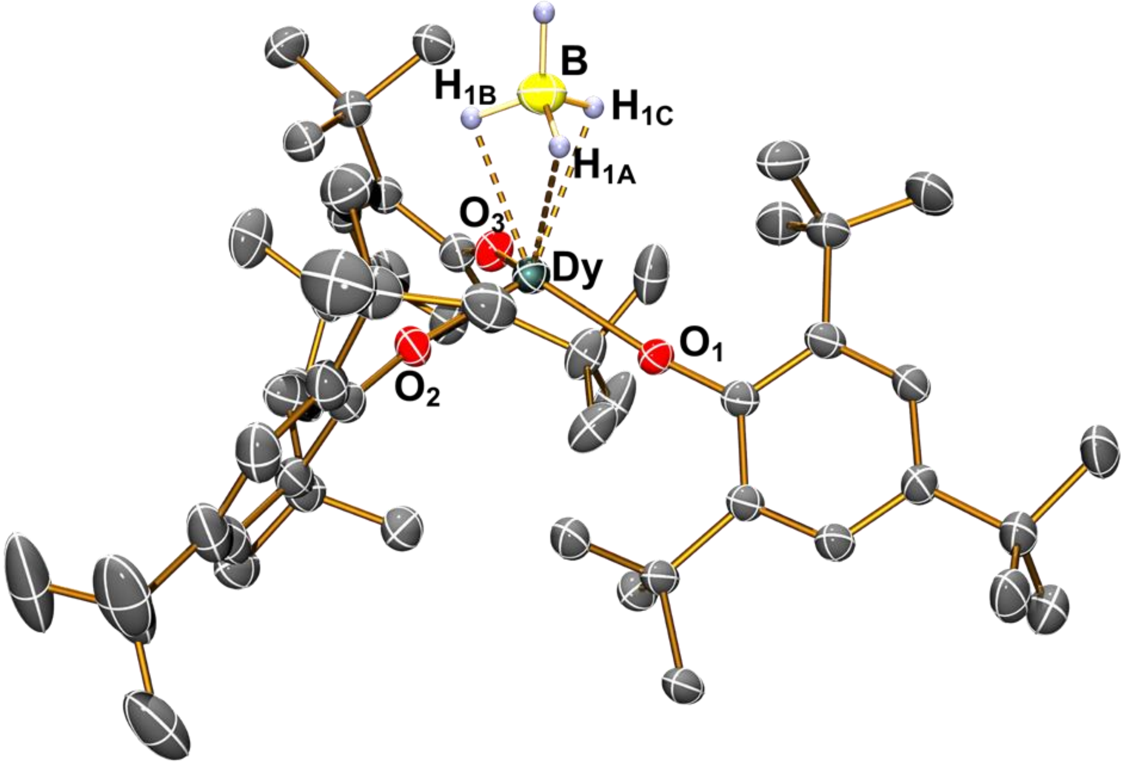

Figure S10. View of the molecular structure of $[Dy(OMes^*)_3BH_4]^-$ (**3**) from single crystal XRD at 150 K with thermal ellipsoids drawn at 40% probability level (Dy teal, B yellow, O red, C grey, H lightblue). H atoms (except for BH_4), counter ion and lattice solvents are omitted for clarity.

Table S2. Selected bond lengths and angles (°) in complexes **1** and **1-Y**.

	**1**	**1-Y**
Ln - O1	2.083(2)	2.063(3)
Ln - O2	2.103(2)	2.076(3)
Ln - Cl1	2.5492(10)	2.537(2)
Ln - Cl2	2.5493(10)	2.5373(14)
O1 - Ln - O2	122.37(8)	120.52(11)
O1 - Ln - Cl1	101.10(6)	102.07(10)
O1 - Ln - Cl2	112.05(6)	112.82(9)
O2 - Ln - Cl1	119.16(6)	119.42(9)
O2 - Ln - Cl2	97.93(6)	97.79(9)
Cl1 - Ln - Cl2	102.88(5)	103.20(6)

Table S3. Selected bond lengths and angles (°) in complex **2**.

**Lengths**	**2**	**Angles**	**2**
Dy - O1	2.116(5)	O1 - Dy - O2	123.2(2)
Dy - O2	2.132(5)	O1 - Dy - O3	106.52(2)
Dy - O3	2.091(6)	O3 - Dy - O2	108.1(2)
Dy - Cl1	2.553(2)	O2 - Dy - Cl1	111.45(13)
		O3 - Dy - Cl1	107.06(14)
		O1 - Dy - Cl1	99.35(14)

Table S4. Selected bond lengths and angles (°) in complex **3**.

**Lengths**	**3**	**Angles**	**3**
Dy - O1	2.144(2)	O1 - Dy - O3	122.76(8)
Dy - O2	2.109(2)	O1 - Dy - O2	108.65(8)
Dy - O3	2.126(2)	O2 - Dy - O3	105.67(8)
Dy···B1	2.537(4)	O1 - Dy - B1	110.04(12)
Dy - H1A	2.30	O2 - Dy - B1	109.22(12)
Dy - H1B	2.40	O3 - Dy - B1	99.75(12)
Dy - H1C	2.42		

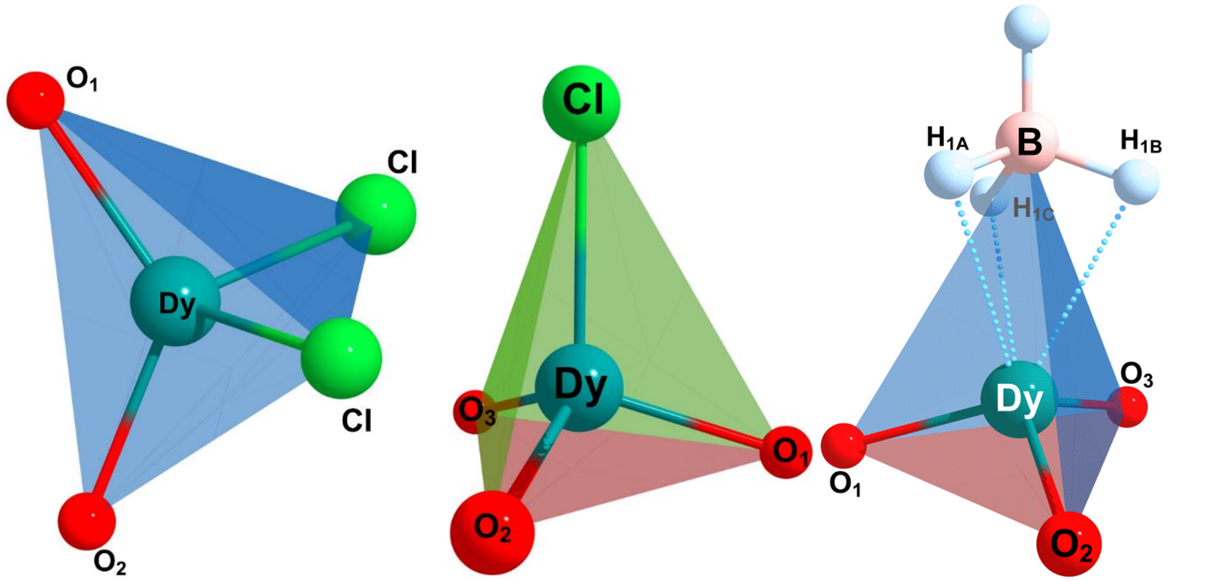

Figure S11. View of the first coordination sphere geometries in **1** (*left*), **2** (*centre*) and **3** (*right*) from single crystal XRD at 150 K (Dy teal, Cl green, O red, B pink, H white).

Table S5. Continuous Shape Measures (**CShM**)[11] calculations for complexes **1**, **1-Y**, **2** and **3**.

**Structure**[a]		SP-4	T-4	SS-4	vTBPY-4
**1**	**CShM**	28.225	1.172	7.405	3.883
**1-Y**	**CShM**	27.865	1.189	7.350	4.023
**2**	**CShM**	29.226	0.854	7.465	3.102
**3**	**CShM**	28.428	0.815	7.376	3.300

[a] SP-4 Square (D_4h)
T-4 Tetrahedron (T_d)
SS-4 Seesaw (C_2v)
vTBPY-4 Vacant trigonal bipyramid (C_3v)

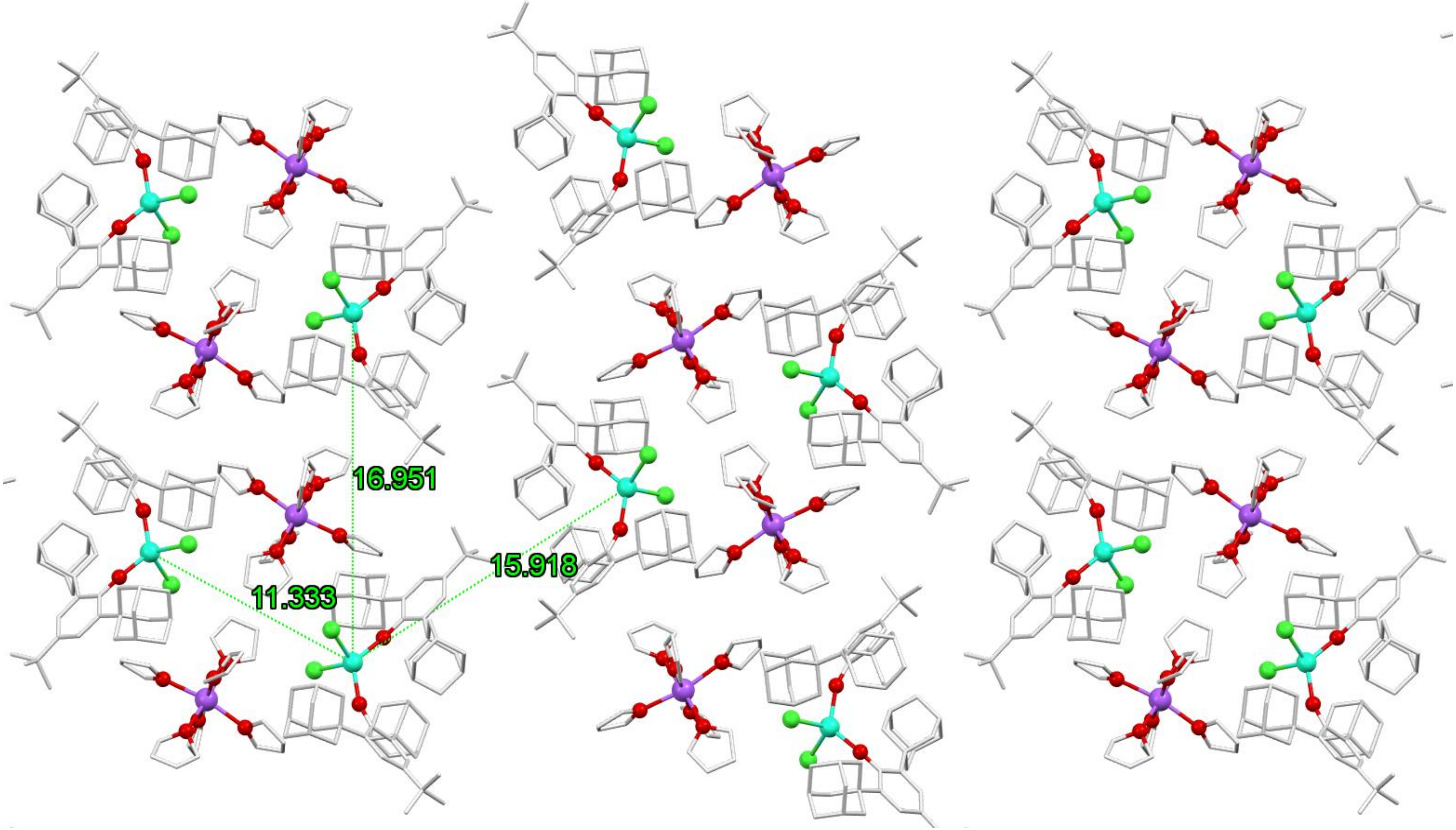

Figure S12. View of the crystal packing in $[Dy(OAr^{Ad2tBu})_2Cl_2]^-$ **1** along the x-axis (Dy turquoise, Cl green, O red, Na purple, C light grey). H atoms and lattice solvent are omitted for clarity.

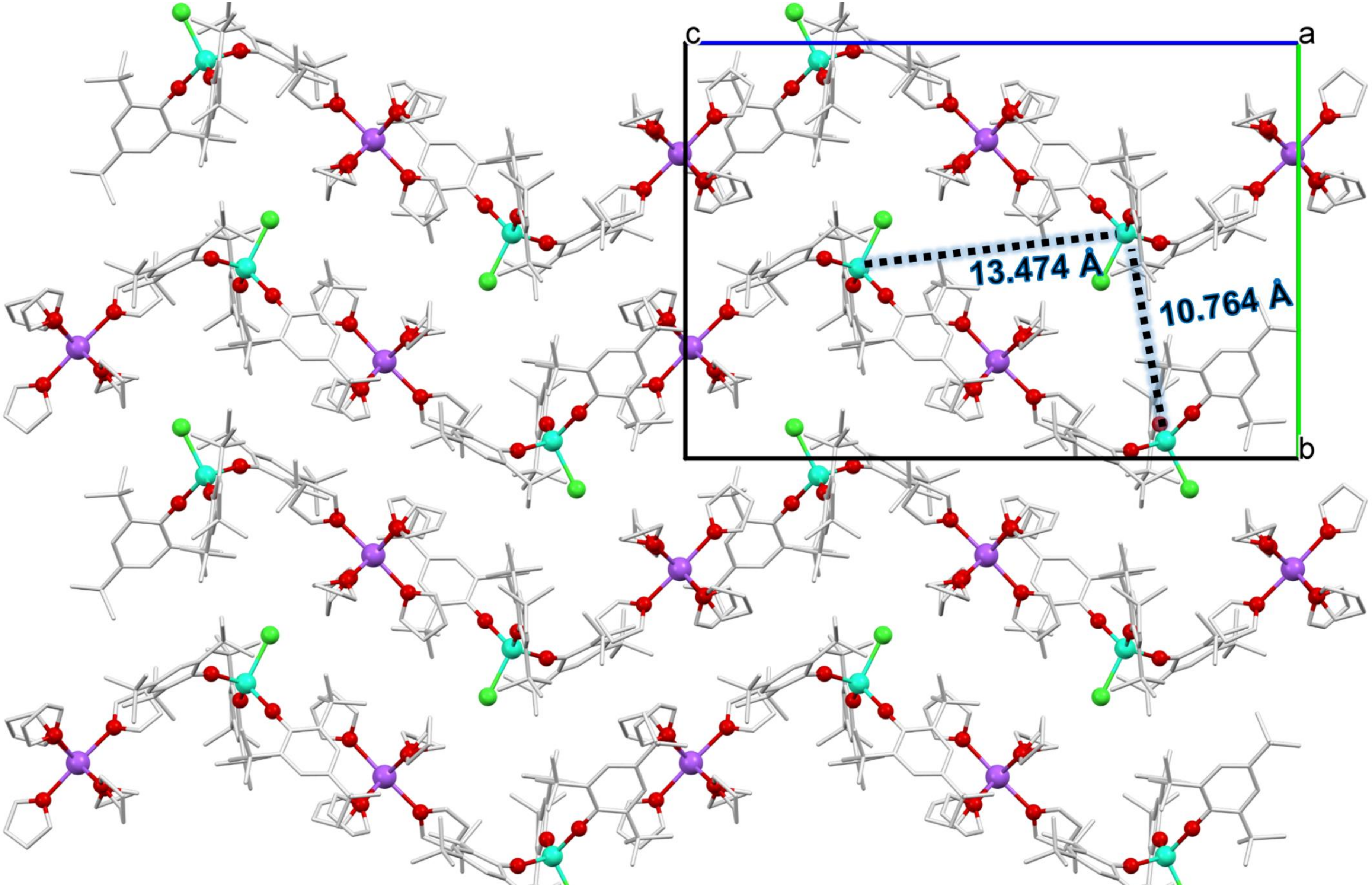

Figure S13. View of the crystal packing in $[Dy(OMes^*)_3Cl]^-$ **2** along the x-axis (Dy turquoise, Cl green, O red, Na purple, C light grey). H atoms and lattice solvent are omitted for clarity.

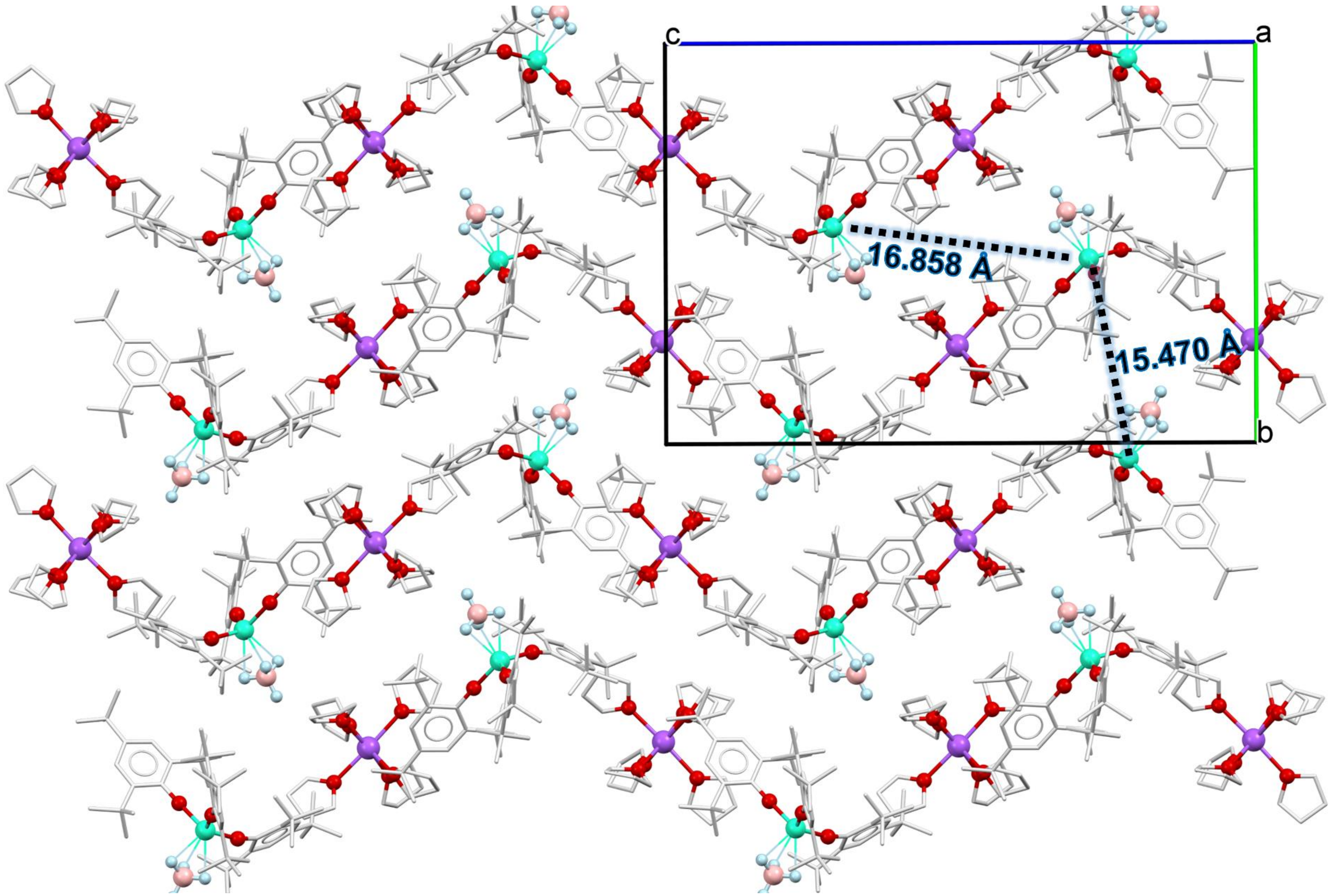

Figure S14. View of the crystal packing in $[Dy(OMes^*)_3BH_4]^-$ **3** along the x-axis (Dy turquoise, B pink, O red, Na purple, C light grey, H light blue). H atoms (except for BH_4) and lattice solvent are omitted for clarity.

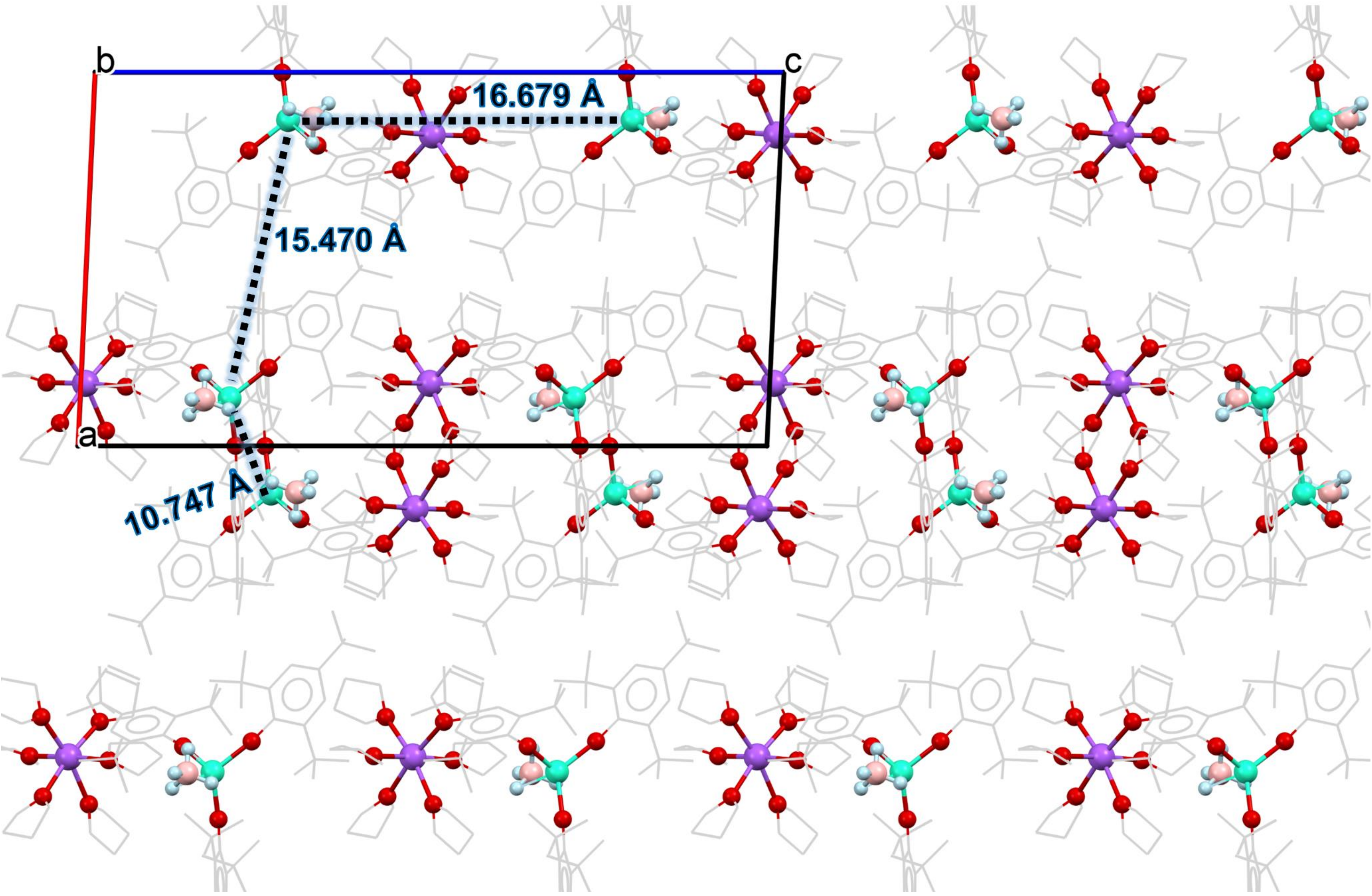

Figure S15. View of the crystal packing in $[Dy(OMes^*)_3BH_4]^-$ **3** along the y-axis (Dy turquoise, B pink, O red, Na purple, C light grey, H light blue). H atoms (except for BH_4) and lattice solvent are omitted for clarity.

4. Computational details

All the first-principle complete active space self-consistent field spin-orbit (CASSCF-SO) calculations were performed with Molcas 8.0[12] and OpenMolcas[13] software packages. The coordinates obtained from single crystal X-ray crystallography were used without optimisation. In the active space, we have performed state-averaged CASSCF calculations with nine electrons in the seven 4f orbitals (i.e. CAS(9,7)) for 21, 128 and 130 roots for the sextet, quartet and doublet spin states, respectively. The basis set for the ab initio calculations were chosen from the ANO-RCC library; [14,15] the Dy atom was treated with VTZP quality, the O, Cl, Br atoms with VDZP and others (C and H atoms) with VDZ quality with scalar relativistic effects accounted using the second-order Douglas–Kroll–Hess transformation. The RASSCF, RASSI and SINGLE_ANISO[16] modules were utilised to compute the g-tensors, CF parameters, energy and mixing of the m_J levels and interstate tunnelling probabilities.

Table S6. *Ab initio* results for the J = 15/2 multiplet of Dy(III) in **1**, **2** and **3**.

	***Ab initio* Energy (cm^{-1})**	g_x	g_y	g_z	**g_z Angle (º)**	**Wavefunction**
**1**	0.00	0.00	0.00	19.72	--	97%|±15/2⟩ + 3%|±11/2⟩
	200.24	0.01	0.01	16.74	6.68	90%|±13/2⟩ + 8%|±9/2⟩
	365.69	0.05	0.07	13.92	9.47	78%|±11/2⟩ + 12%|±7/2⟩ + 4%|±9/2⟩ + 2%|±15/2⟩ + 2%|±13/2⟩ + 2%|±5/2⟩
	511.41	0.66	0.77	11.39	14.27	64%|±9/2⟩ + 12%|±7/2⟩ + 11%|±5/2⟩ + 7%|±13/2⟩ + 3%|±11/2⟩ + 3%|±3/2⟩
	629.69	1.27	2.24	9.19	25.99	47%|±7/2⟩ + 28%|±5/2⟩ + 12%|±11/2⟩ + 6%|±3/2⟩ + 4%|±1/2⟩ + 3%|±9/2⟩
	715.55	1.35	4.91	7.03	45.18	52%|±3/2⟩ + 26%|±5/2⟩ + 13%|±9/2⟩ + 7%|±1/2⟩ + 2%|±7/2⟩
	766.50	2.01	6.98	12.46	88.28	67%|±1/2⟩ + 12%|±7/2⟩ + 10%|±3/2⟩ + 8%|±5/2⟩ + 2%|±9/2⟩
	890.47	0.09	0.18	19.57	74.38	29%|±3/2⟩ + 26%|±5/2⟩ + 21%|±1/2⟩ + 15%|±7/2⟩ + 6%|±9/2⟩ + 2%|±11/2⟩
**2**	0.00	0.01	0.01	19.74	--	97%|±15/2⟩ + 2%|±11/2⟩
	178.38	0.38	0.75	16.32	1.36	30%|±13/2⟩ + 3%|±9/2⟩
	274.05	3.19	4.70	13.06	77.42	27%|±11/2⟩ + 20%|±3/2⟩ + 19%|±7/2⟩
	340.72	0.86	4.92	7.63	81.88	46%|±11/2⟩ + 2%|±9/2⟩ + 2%|±7/2⟩ + 2%|±5/2⟩ + 2%|±3/2⟩
	436.67	2.41	2.70	11.23	82.69	34%|±9/2⟩ + 7%|±7/2⟩ + 5%|±3/2⟩ + 4%|±11/2⟩ + 3%|±13/2⟩ + 3%|±5/2⟩ + 3%|±1/2⟩
	535.13	0.02	0.17	14.59	86.42	32%|±7/2⟩ + 6%|±11/2⟩ + 4%|±5/2⟩ + 2%|±9/2⟩
	633.83	0.14	0.16	17.34	83.05	25%|±5/2⟩ + 8%|±1/2⟩ + 6%|±3/2⟩ + 3%|±9/2⟩ + 3%|±7/2⟩
	723.46	0.02	0.03	19.56	86.94	23%|±1/2⟩ + 11%|±3/2⟩ + 9%|±5/2⟩
**3**	1.00	0.02	0.03	19.70	--	97%|±15/2⟩ + 3%|±11/2⟩
	138.62	1.63	6.75	13.20	74.43	36%|±13/2⟩ + 16%|±1/2⟩ + 14%|±3/2⟩ + 12%|±5/2⟩ + 11%|±9/2⟩ + 3%|±11/2⟩ + 3%|±7/2⟩
	171.64	2.77	4.97	8.60	6.73	55%|±13/2⟩ + 12%|±3/2⟩ + 12%|±1/2⟩ + 6%|±7/2⟩ + 6%|±5/2⟩ + 5%|±11/2⟩ + 2%|±9/2⟩
	272.22	5.20	5.88	8.63	27.83	58%|±11/2⟩ + 11%|±7/2⟩ + 11%|±5/2⟩ + 8%|±9/2⟩ + 5%|±1/2⟩ + 3%|±13/2⟩ + 2%|±15/2⟩
	350.58	1.21	2.04	11.34	77.70	38%|±9/2⟩ + 20%|±11/2⟩ + 16%|±3/2⟩ + 9%|±5/2⟩ + 7%|±7/2⟩ + 4%|±13/2⟩ + 3%|±1/2⟩
	451.98	0.43	0.56	14.28	87.57	26%|±9/2⟩ + 16%|±1/2⟩ + 15%|±7/2⟩ + 13%|±5/2⟩ + 7%|±11/2⟩ + 6%|±3/2⟩ + 2%|±13/2⟩
	572.58	0.10	0.12	16.97	83.77	30%|±5/2⟩ + 21%|±3/2⟩ + 12%|±9/2⟩ + 9%|±1/2⟩ + 4%|±7/2⟩ + 3%|±11/2⟩
	692.93	0.01	0.02	19.65	84.79	39%|±1/2⟩ + 31%|±3/2⟩ + 19%|±5/2⟩ + 3%|±9/2⟩

Table S7. Ab initio calculated crystal field parameters in **1**, **2** and **3**.

B_k^q	**1**	**2**	**3**
B_2^{-2}	-2.30E-01	-6.48E-02	4.10E-02
B_2^{-1}	5.36E-01	2.23E-01	-4.51E-02
B_2^0	-4.60E+00	3.30E+00	3.44E+00
B_2^1	-7.36E-01	-4.17E-02	-3.87E-02
B_2^2	2.59E+00	-3.03E+00	-1.85E+00
B_4^{-4}	-2.88E-03	1.89E-03	-2.76E-03
B_4^{-3}	9.53E-04	-8.33E-02	7.18E-02
B_4^{-2}	1.25E-03	-5.94E-03	1.08E-02
B_4^{-1}	-6.89E-04	1.08E-02	-5.82E-03
B_4^0	-6.17E-04	-2.69E-03	-6.15E-04
B_4^1	2.42E-02	6.99E-03	8.79E-03
B_4^2	2.21E-02	-5.44E-03	-6.78E-03
B_4^3	1.84E-04	-5.99E-03	-3.28E-03
B_4^4	1.31E-02	-2.31E-02	-2.72E-02
B_6^{-6}	-1.79E-05	5.70E-05	-5.85E-05
B_6^{-5}	1.20E-04	1.35E-04	-4.79E-07
B_6^{-4}	-1.13E-05	4.02E-05	-4.17E-06
B_6^{-3}	-9.37E-05	-6.18E-05	-1.13E-05
B_6^{-2}	2.93E-05	-3.44E-06	2.71E-05
B_6^{-1}	8.09E-05	-6.05E-06	-6.85E-05
B_6^0	-5.49E-07	-2.93E-06	-4.17E-06
B_6^1	-5.18E-05	-1.95E-05	-3.83E-05
B_6^2	-9.66E-05	4.09E-06	7.44E-05
B_6^3	3.08E-05	-3.56E-05	-9.88E-05
B_6^4	4.70E-05	-2.47E-05	4.85E-05
B_6^5	-2.37E-04	1.16E-04	-2.10E-05
B_6^6	-1.94E-05	3.45E-05	1.35E-05

Calculation of τ_{QTM} and estimation of U_{eff} from CASSCF-SO results:[17]

Yin *et al.* recently reported a method to estimate the relaxation time of QTM and the effective barrier of magnetic reversal for a Kramers SMM using the results from the CASSCF-SO/Single_Aniso calculated g_x, g_y and g_z factors.[18] The whole procedure is described in reference 18, which describes the rate of QTM as:

$$\tau_{\mathrm{QTM}}^{-1} = \frac{\beta B_{\mathrm{ave}}}{2h} \frac{g_{xy}^2}{\left(g_{xy}^2 + g_z^2\right)^{1/2}} \; ; \quad g_{xy} = \left(g_x^2 + g_y^2\right)^{\frac{1}{2}} \tag{1}$$

Where B_{ave} is the average local magnetic field at the Dy(III) centre due to the dipolar interactions with its neighbouring molecules in the crystal. The QTM rate within excited KDs could also be calculated using the corresponding g factors. With the assumption that all the KDs experience the same local magnetic field and accounting for the respective Boltzmann population at all the KDs, the U_{eff} can be estimated as below:

$$\tau_{\mathrm{QTM},i}^{-1}(T) \propto \left(\frac{\beta B_{\mathrm{ave}}}{2h} \frac{g_{xy}^2}{\left(g_{xy}^2 + g_z^2\right)^{\frac{1}{2}}} \right) \cdot \frac{\exp(-\frac{Ei}{k_{\mathrm{B}}T})}{\sum_i \exp\left(-\frac{Ei}{k_{\mathrm{B}}T}\right)} \tag{2}$$

$$U_{\mathrm{eff}}\,(T) = \sum\nolimits_i \frac{\tau_{\mathrm{QTM},i}^{-1}(T)}{\sum_i \tau_{\mathrm{QTM},i}^{-1}(T)} \tag{3}$$

To calculate B_{ave} for complexes **1** - **3**, we have performed classical simulations of the dipolar magnetic field in each compound. Taking the crystallographic coordinates of each compound, we simulate a classical magnetic dipole S = 1/2 with anisotropy given by the effective g-values from CASSCF-SO (Table S3) and performed stochastic spin flips under the field of all dipoles in a sphere of 40 Å radius. The estimated average dipolar field at the Dy(III) centre was found to be 149(67), 170(58) and 156(57) Oe for **1**, **2** and **3**, respectively. This was then used to calculate the TA-QTM rates and U_{eff} (*T*) as shown above.

Table S8. Calculated $g_{x\text{-}y}$ and rate and relaxation time of QTM process within the TA-QTM framework for **1**, **2** and **3** using a recently reported approach by Yin *et al.*[18]

	Energy (cm^{-1})	g_x	g_y	g_z	$g_{x\text{-}y}$	τ_{QTM}^{-1} (s^{-1})	τ_{QTM} (s)
**1**	0.00	0.00	0.00	19.72	0.00	7.10E+00	7.05E-02
	200.24	0.01	0.01	16.74	0.02	2.31E+03	2.17E-04
	365.69	0.05	0.07	13.92	0.09	7.78E+04	6.43E-06
	511.41	0.66	0.77	11.39	1.02	1.26E+07	3.96E-08
	629.69	1.27	2.24	9.19	2.58	9.75E+07	5.13E-09
	715.55	1.35	4.91	7.03	5.09	4.18E+08	1.20E-09
	766.50	2.01	6.98	12.46	7.27	5.12E+08	9.76E-10
	890.47	0.09	0.18	19.57	0.20	2.88E+05	1.74E-06
**2**	0.00	0.01	0.01	19.74	0.01	8.82E+02	5.67E-04
	178.38	0.38	0.75	16.32	0.85	6.12E+06	8.17E-08
	274.05	3.19	4.70	13.06	5.68	3.17E+08	1.58E-09
	340.72	0.86	4.92	7.63	4.99	3.82E+08	1.31E-09
	436.67	2.41	2.70	11.23	3.61	1.55E+08	3.23E-09
	535.13	0.02	0.17	14.59	0.17	2.77E+05	1.81E-06
	633.83	0.14	0.16	17.34	0.21	3.52E+05	1.42E-06
	723.46	0.02	0.03	19.56	0.03	8.74E+03	5.72E-05
**3**	1.00	0.02	0.03	19.70	0.03	6.45E+03	7.75E-05
	138.62	1.63	6.75	13.20	6.95	4.53E+08	1.10E-09
	171.64	2.77	4.97	8.60	5.69	4.39E+08	1.14E-09
	272.22	5.20	5.88	8.63	7.85	7.39E+08	6.77E-10
	350.58	1.21	2.04	11.34	2.37	6.80E+07	7.35E-09
	451.98	0.43	0.56	14.28	0.71	4.94E+06	1.01E-07
	572.58	0.10	0.12	16.97	0.15	1.98E+05	2.53E-06
	692.93	0.01	0.02	19.65	0.02	2.79E+03	1.79E-04

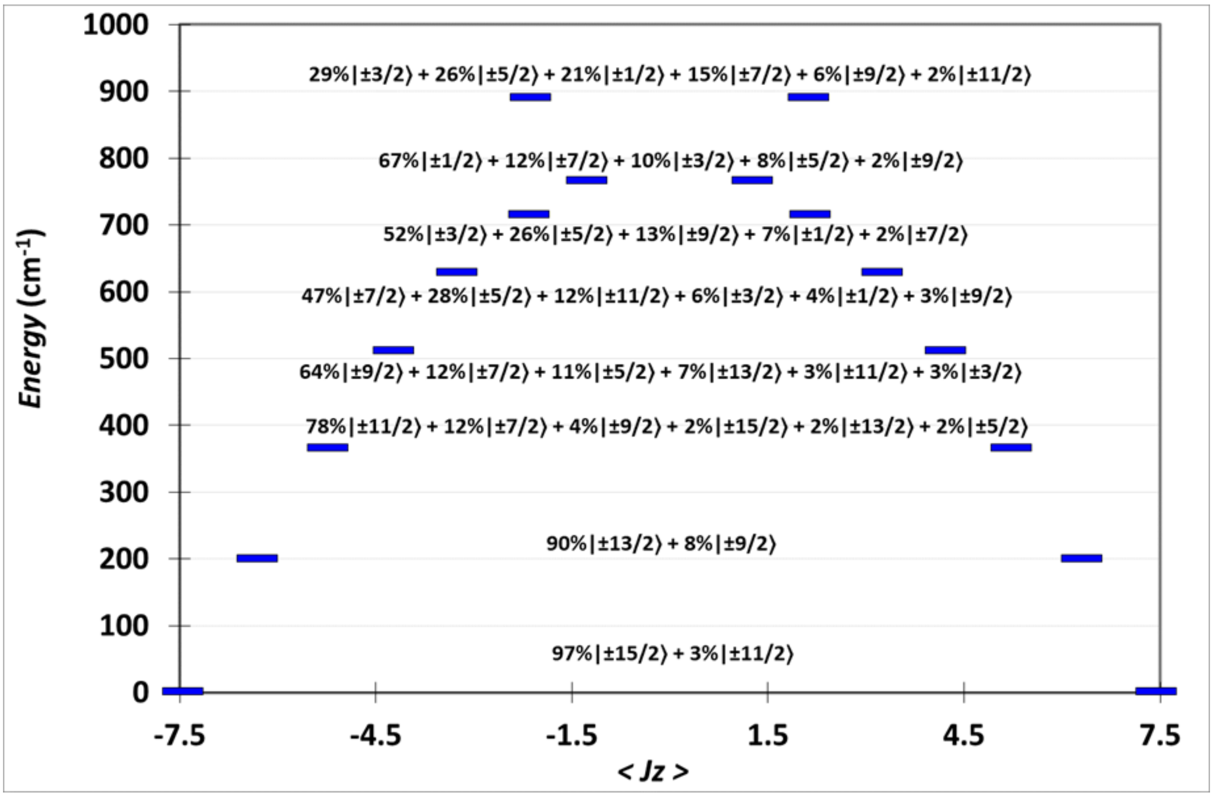

Figure S16. CASSCF-SO calculated m_J levels and J-J mixing for **1**. The percentages of mixing have been rounded off to nearest whole numbers and the contributions lower than 1% have been omitted for brevity.

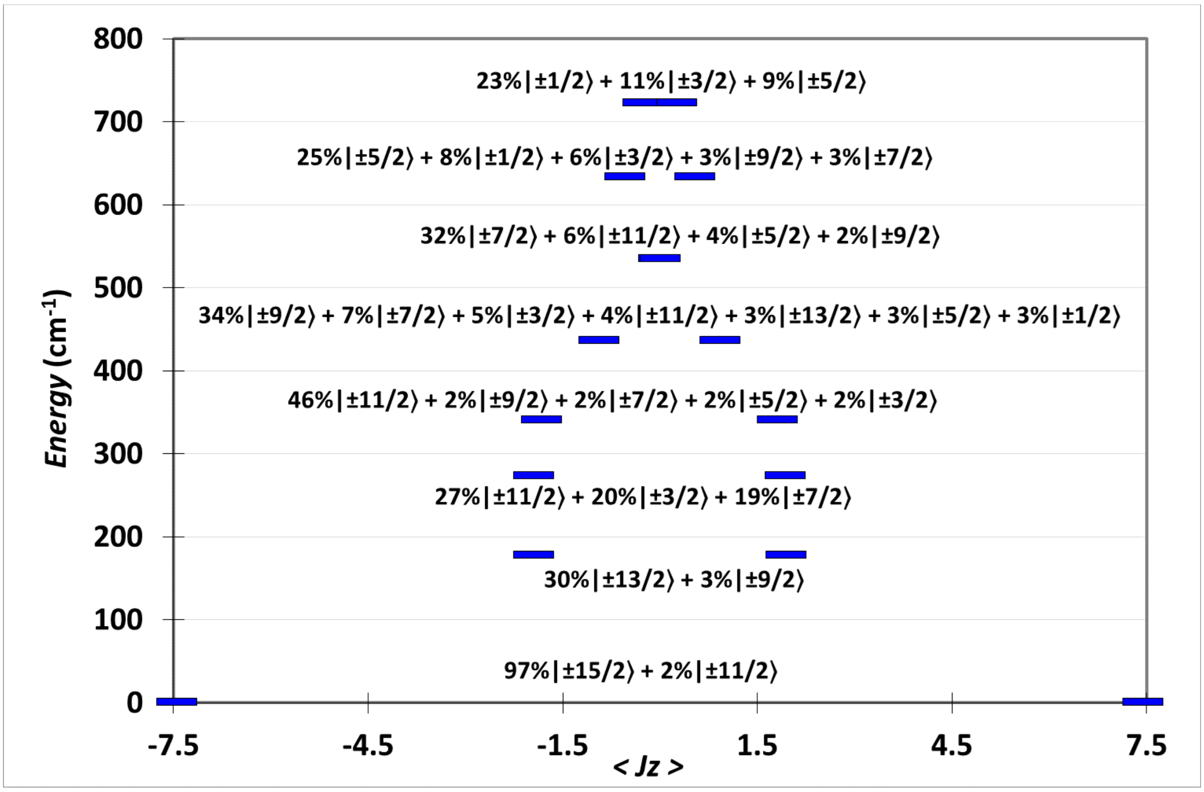

Figure S17. CASSCF-SO calculated m_J levels and J-J mixing for **2**. The percentages of mixing have been rounded off to nearest whole numbers and the contributions lower than 1% have been omitted for brevity.

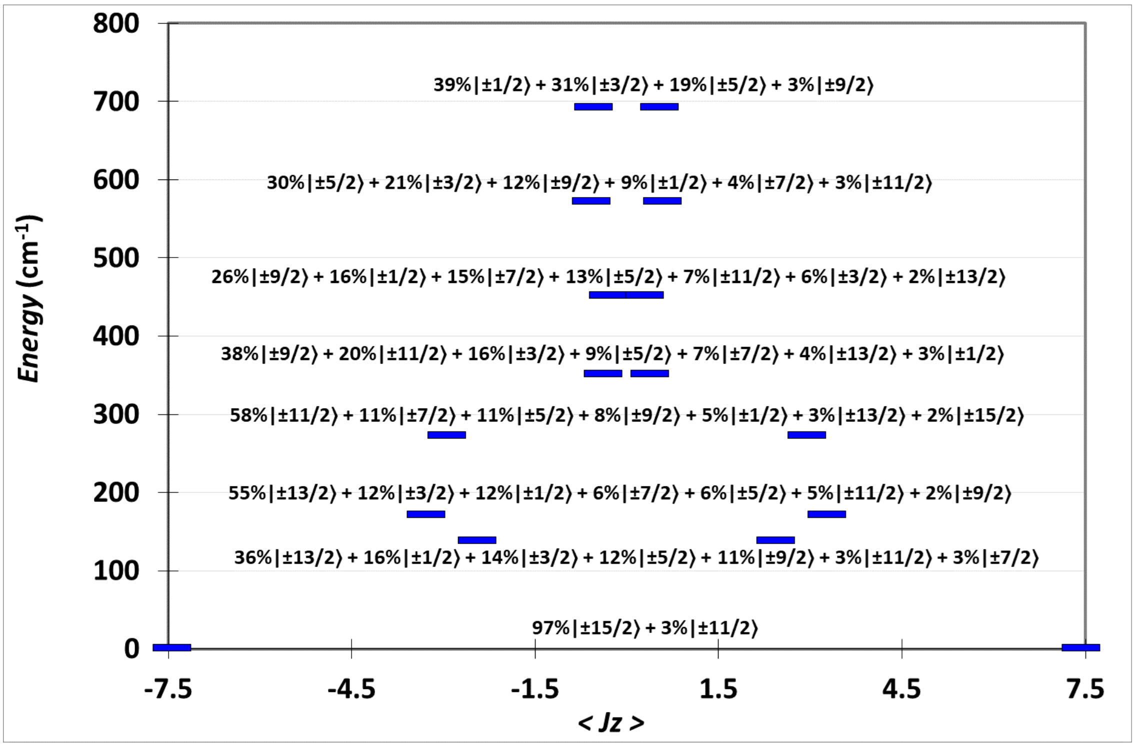

Figure S18. CASSCF-SO calculated m_J levels and J-J mixing for **3**. The percentages of mixing have been rounded off to whole numbers and the contributions lower than 5% have been omitted for clarity.

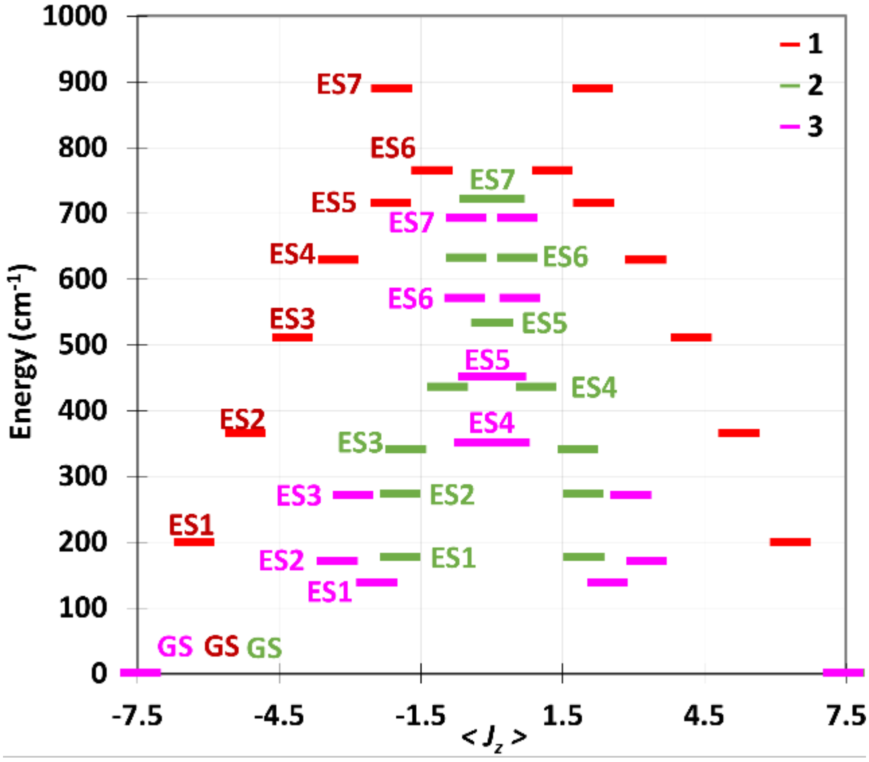

Figure S19. *Top*: CASSCF-SO-calculated energy diagram of the ground-state multiplet for **1**, indicating the zero-field magnetic transition propensities obtained from the average of the three Cartesian transition magnetic moment operators. The opacity of each arrow is proportional to the normalised transition propensity. The percentages of mixing have been rounded off to whole numbers and the contributions lower than 10% have been omitted for brevity. *Bottom*: CASSCF-SO-calculated energy levels of **2** (green) and **3** (magenta) compared to **1** (red).

Table S9. CASSCF-SO computed LoProp charges per atom for **1**, **2** and **3**.

**1**		**2**		**3**	
**Atoms**	**Charges**	**Atoms**	**Charges**	**Atoms**	**Charges**
Dy	2.4479	Dy	2.488	Dy	2.5204
O1	-0.8418	O1	-0.9526	O1	-0.9597
O2	-0.8417	O2	-0.9644	O2	-0.9517
Cl1	-0.9508	O3	-0.9563	O3	-0.9464
Cl2	-0.9531	Cl	-0.8423	B	-0.9017

5. Magnetism

Magnetic measurements were made using a superconducting quantum interference device (SQUID) magnetometer, Quantum Design MPMS 3 for **1** and **5%Dy@1-Y**, and Quantum Design MPMS-XL7 for **2** and **3**. Crystalline samples with masses ranging between 15 and 70 mg were crushed with a mortar and pestle under an inert atmosphere, and then loaded into a borosilicate glass NMR tube along with ca. 5 - 20 mg powdered eicosane, which was then evacuated and flame-sealed to a length of ca. 5 cm. The eicosane was melted by heating the tube gently with a low-power heat gun in order to immobilise the crystallites. The NMR tube was then mounted in the centre of a drinking straw using friction by wrapping it with Kapton tape, and the straw was then fixed to the end of the sample rod. The measurements were corrected for the diamagnetism of the straw, borosilicate tube and eicosane using calibrated blanks, and the intrinsic diamagnetism of the sample as $-0.5\times Mw\times10^{-6}$.

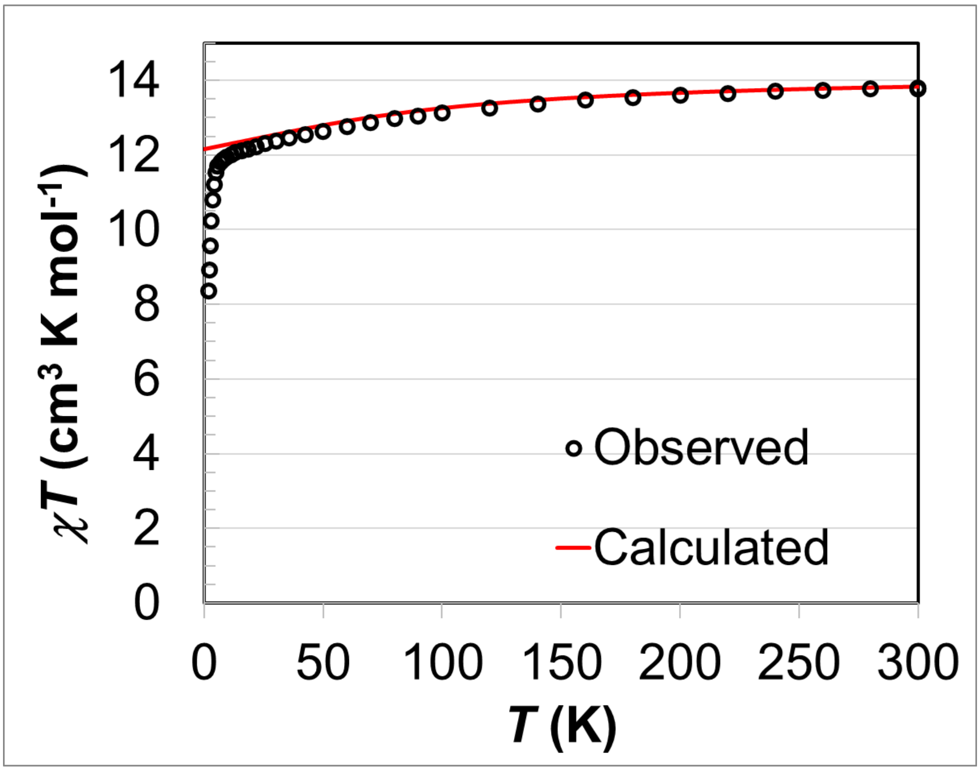

Figure S20. Temperature dependence of the χT product at 0.1 T between 0 and 300 K for **1**. χ is defined as magnetic susceptibility equal to M/H per mole of complex. A shape correction factor of 0.954 as obtained from the MPMS 3 internal program was applied to the observed data.

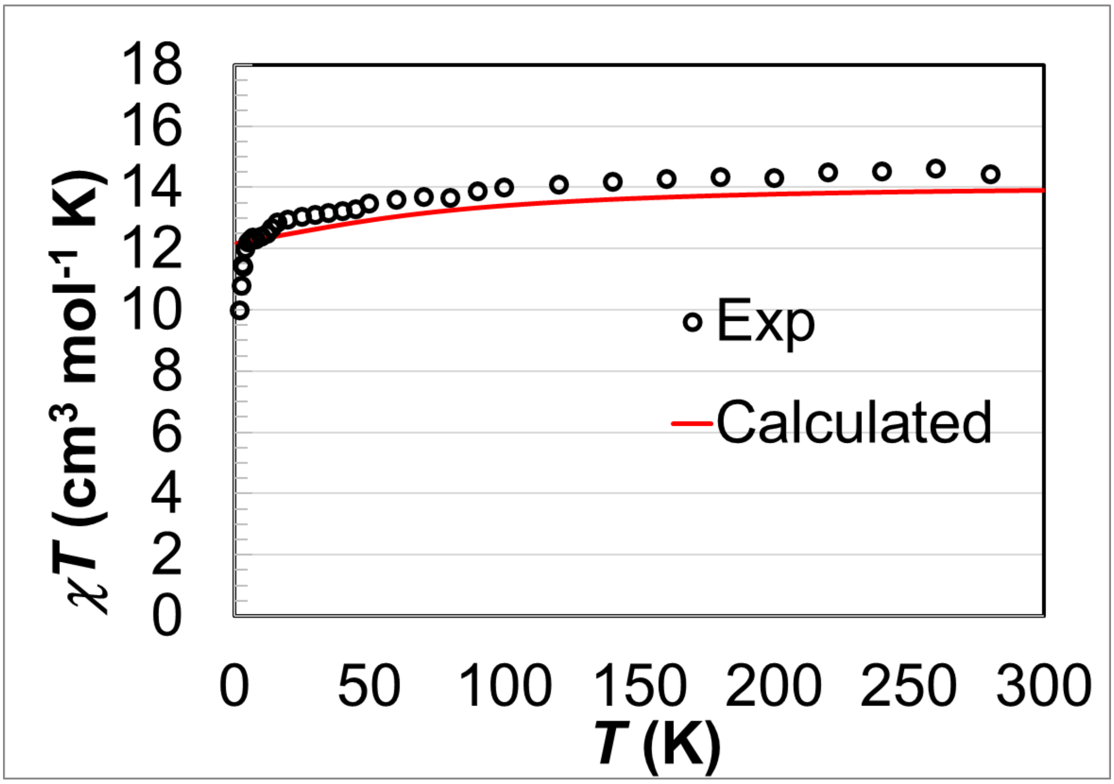

Figure S21. Temperature dependence of the χT product at 0.1 T between 0 and 300 K for **2**. χ is defined as magnetic susceptibility equal to M/H per mole of complex.

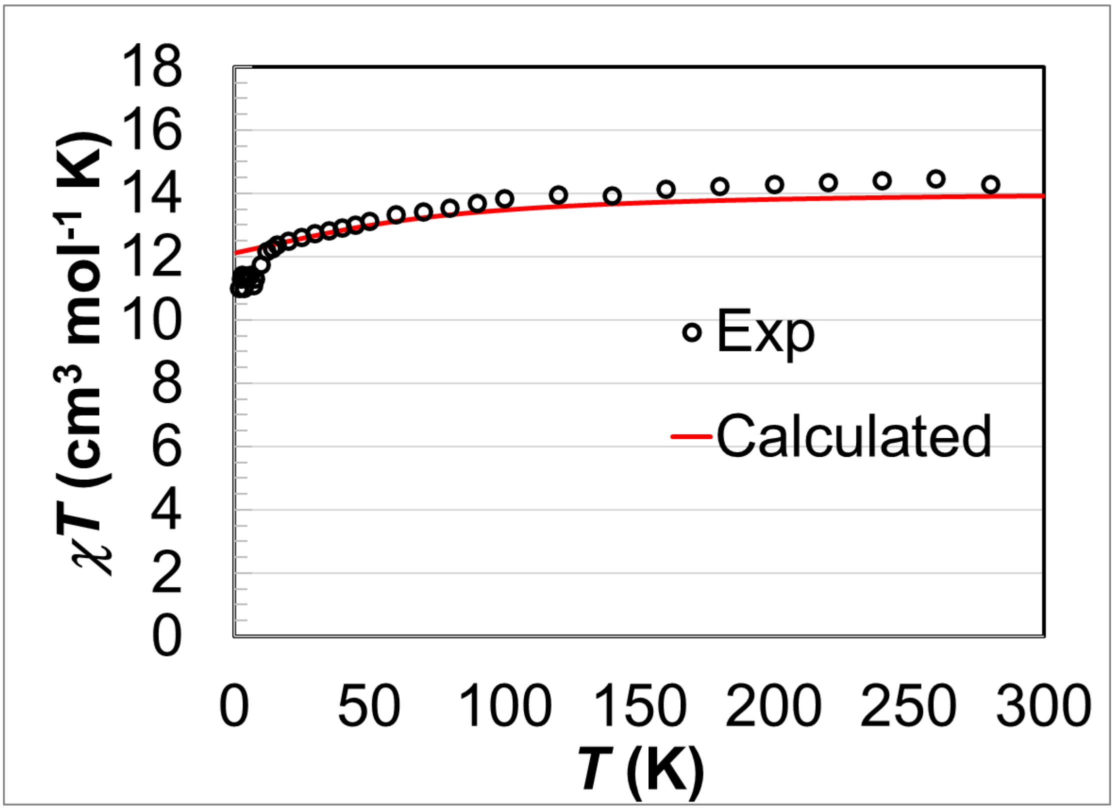

Figure S22. Temperature dependence of the χT product at 0.1 T between 0 and 300 K for **3**. χ is defined as magnetic susceptibility equal to M/H per mole of complex.

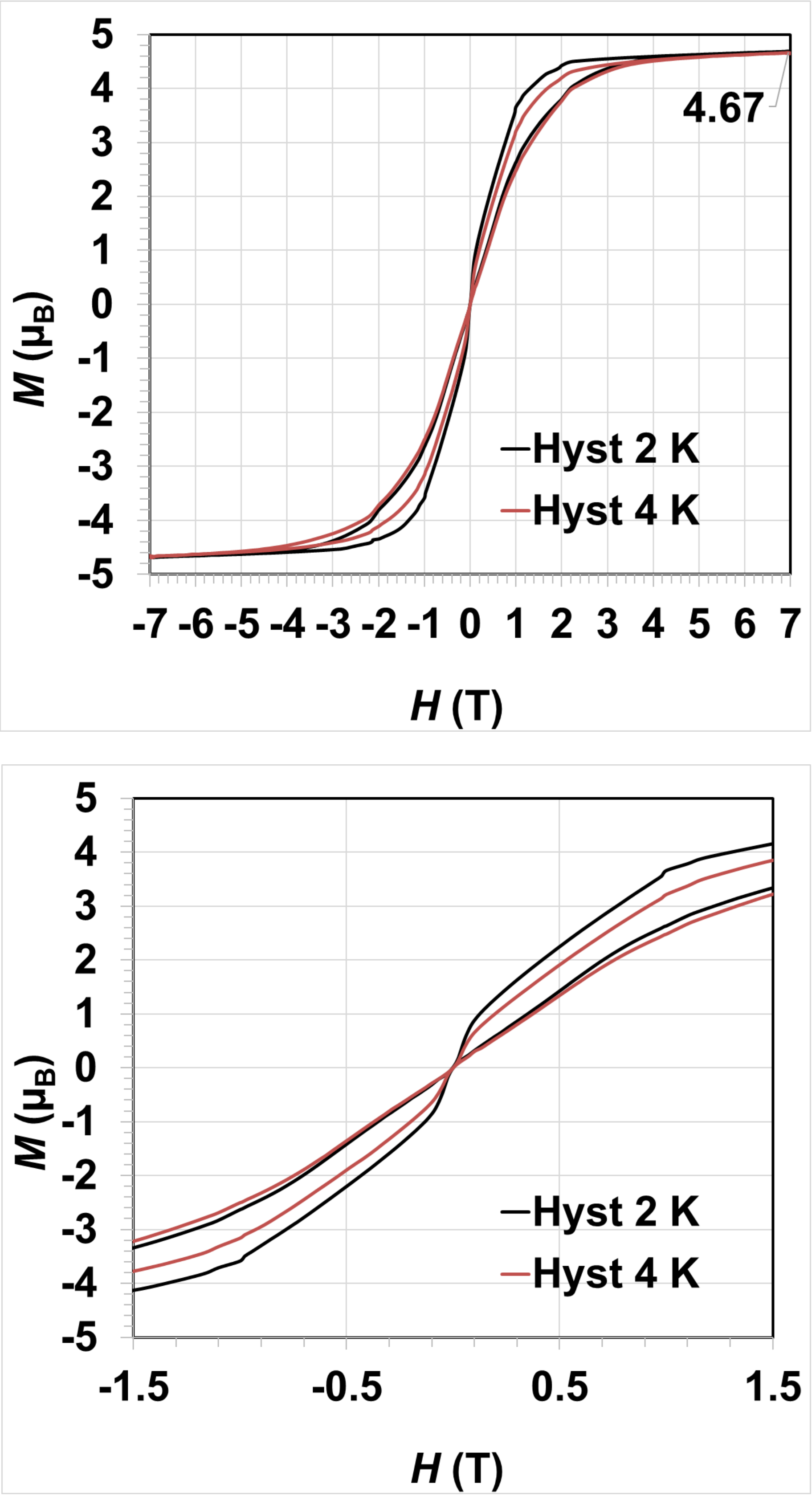

Figure S23. Magnetic hysteresis loops of **1** at 2 and 4 K from -7 T to 7 T (top) and -1.5 T to 1.5 T (bottom). Field sweep rates: 2 < |H| < 7 T, 91 Oe/s; 1 < |H| < 2 T, 52 Oe/s and |H| < 1 T, 22 Oe/s.

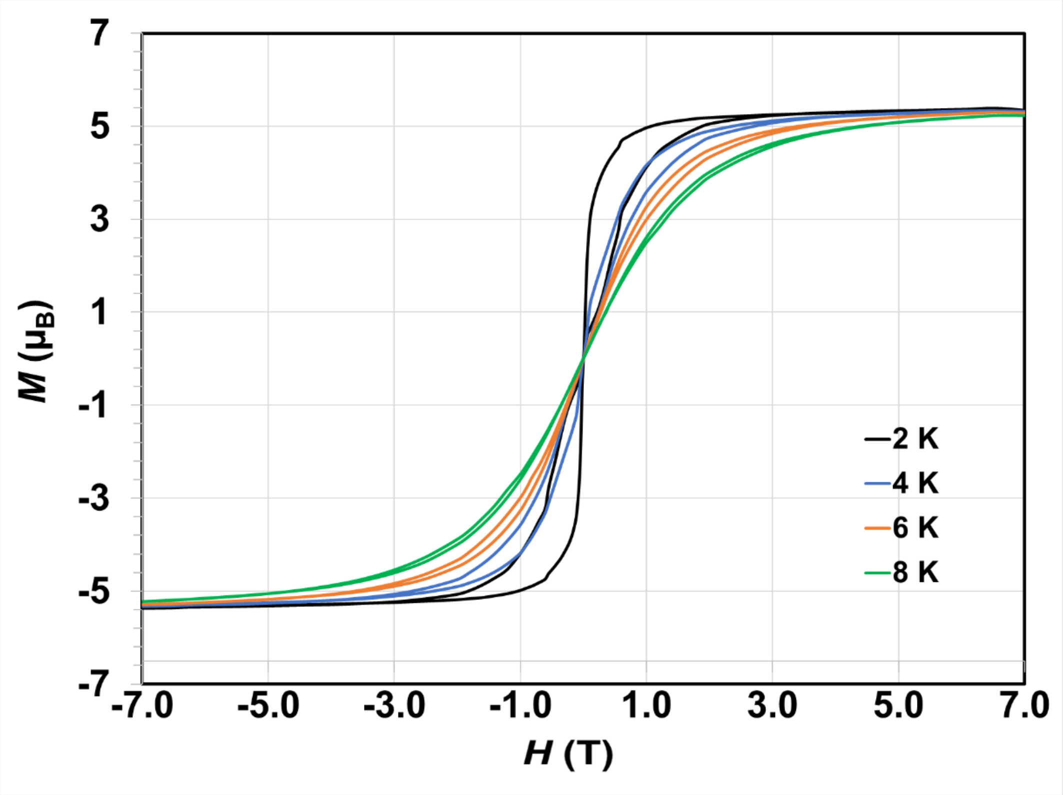

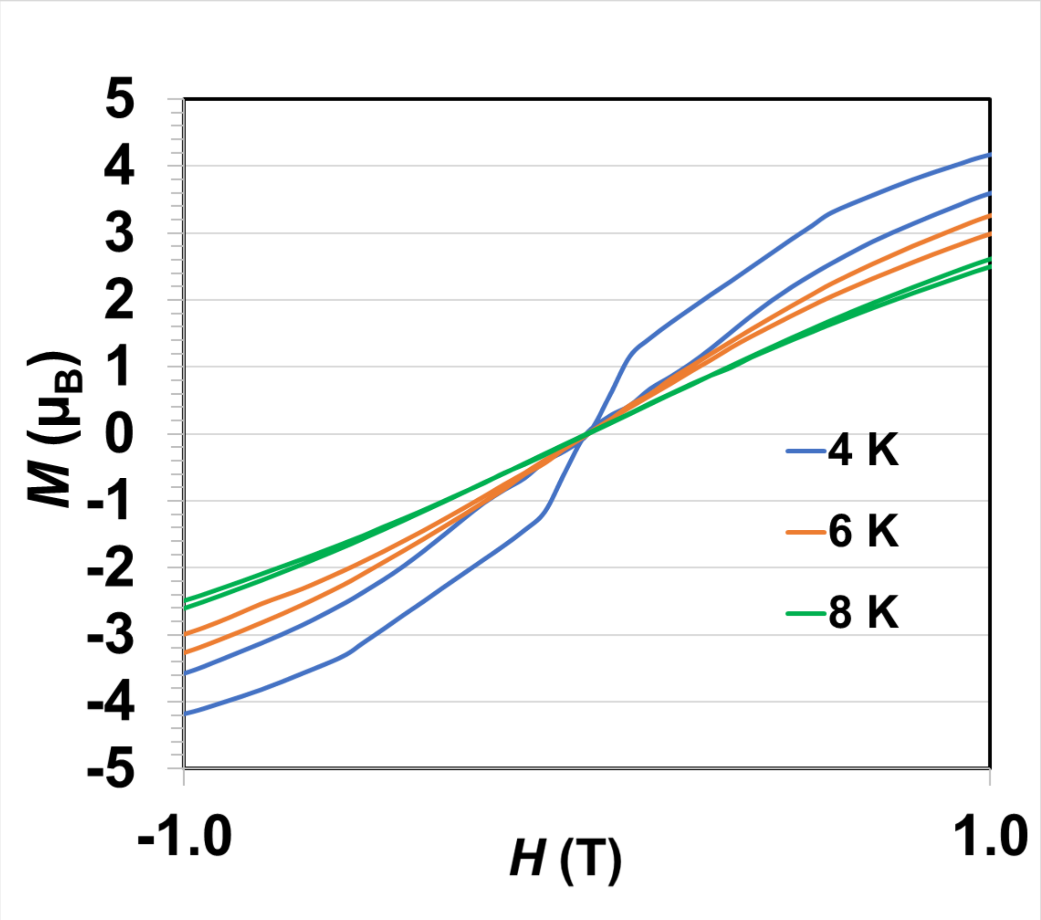

Figure S24. Magnetic hysteresis loops of **2** at 2, 4 and 6 K from -7 T to 7 T (top) and -1 T to 1 T (bottom). Field sweep rates: 1.2 < |H| < 7 T, 71 Oe/s; and |H| < 1 T, 15 Oe/s.

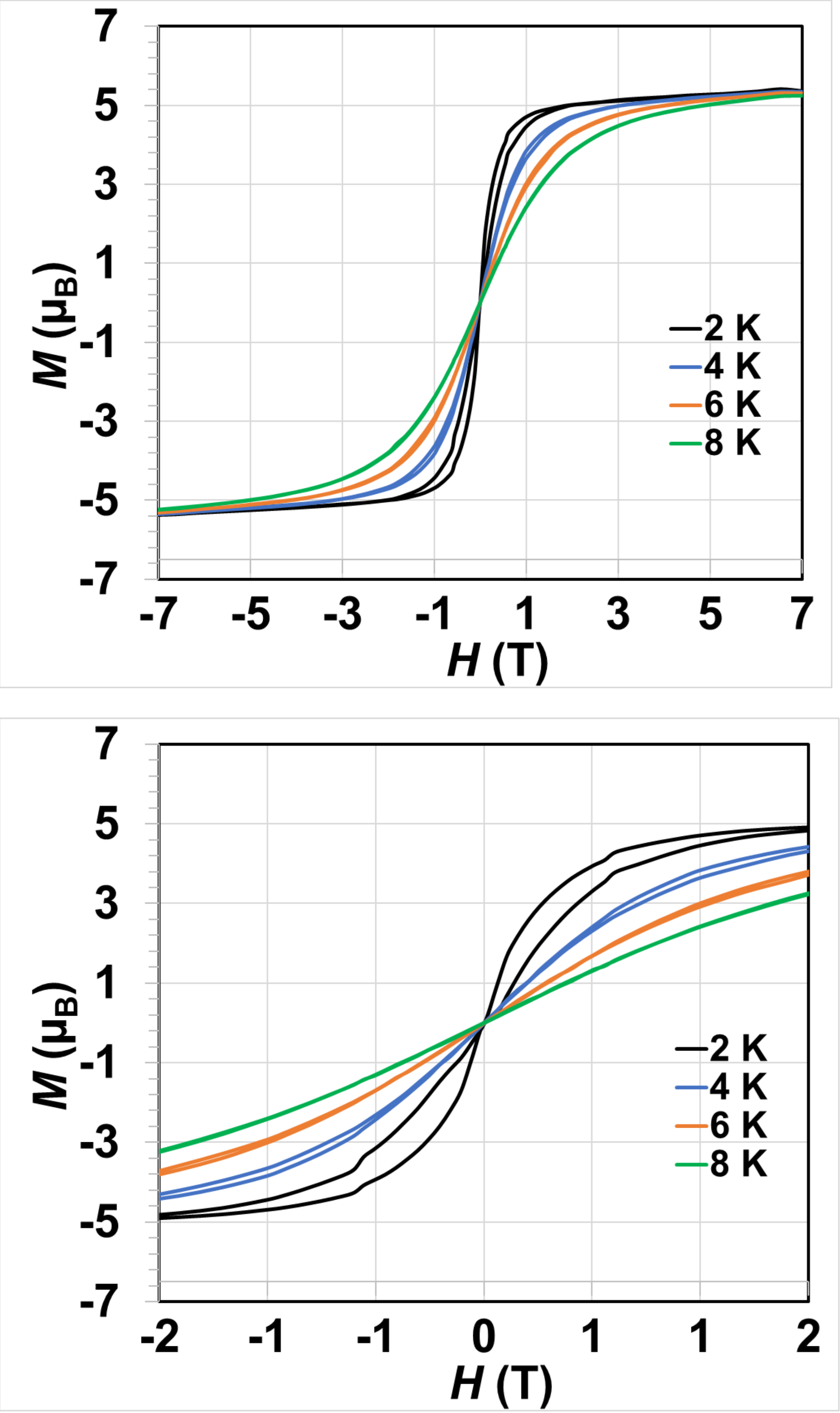

Figure S25. Magnetic hysteresis loops of **3** at 2, 4, 6 and 8 K from -7 T to 7 T (top) and -2 T to 2 T (bottom). Field sweep rates: 1.2 < |H| < 7 T, 71 Oe/s; and |H| < 1 T, 15 Oe/s.

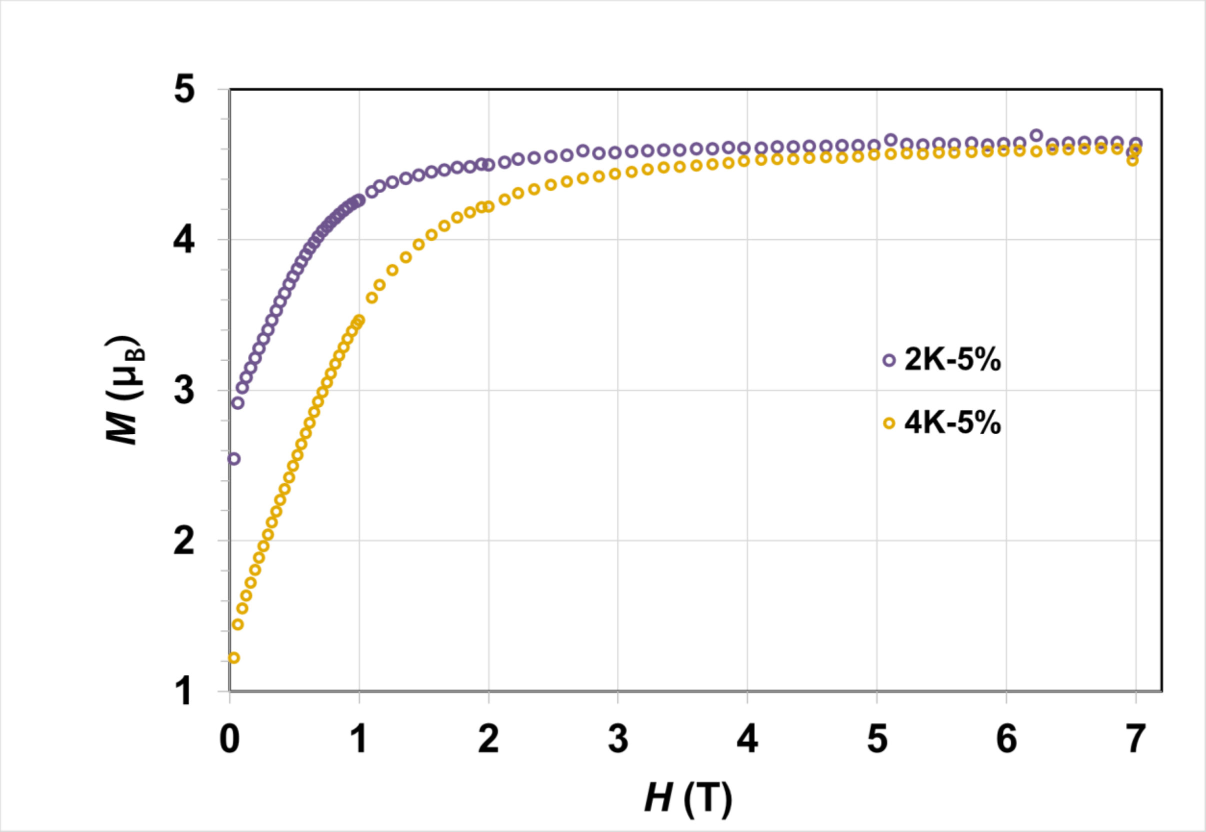

Figure S26. Magnetisation (M) vs Field (H) for **5%Dy@1-Y** at 2 and 4 K (when normalised per mole of Dy complex) from 0 to 7 T.

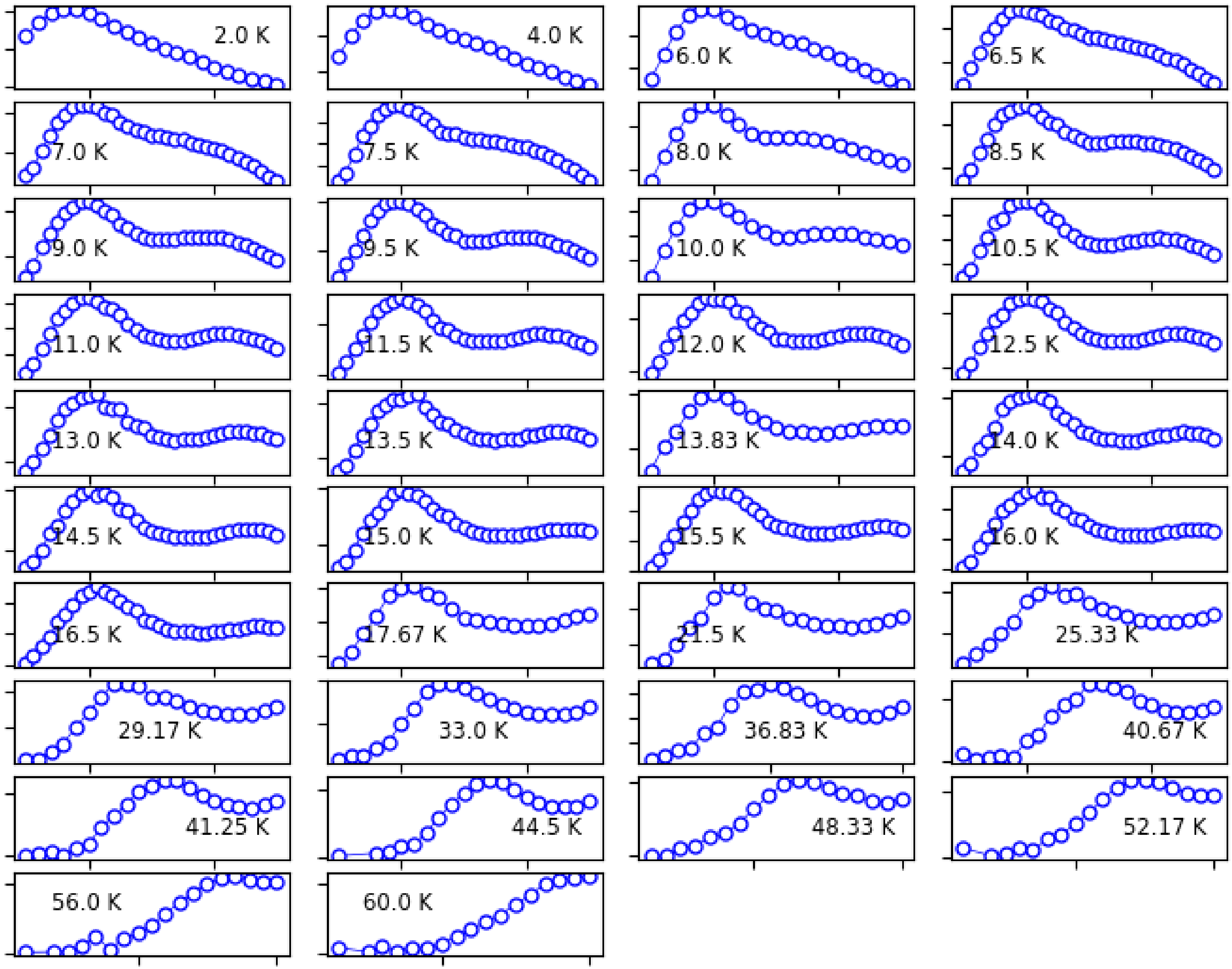

Figure S27. Out of phase susceptibility plots at zero applied field for **1** from 2 – 60 K.

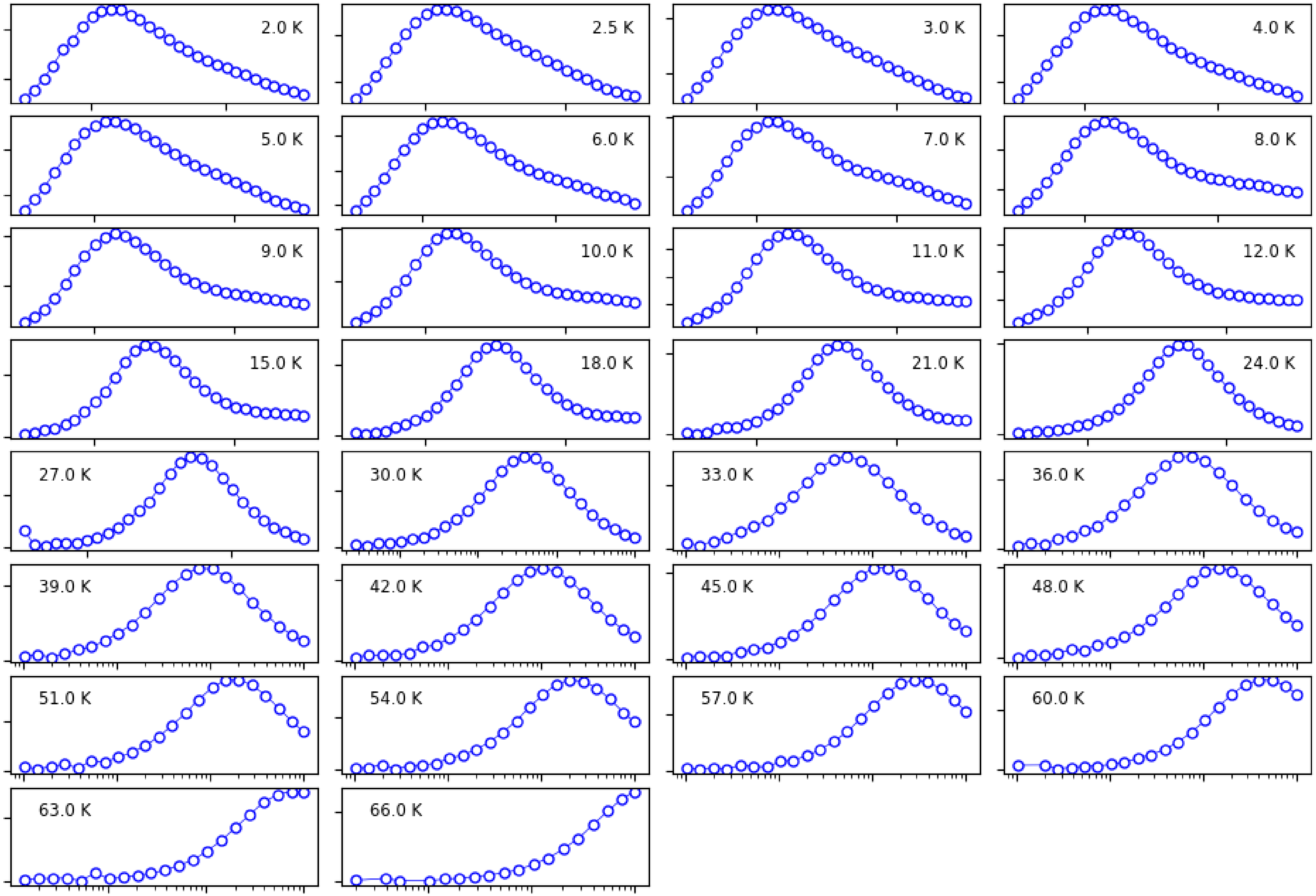

Figure S28. Out of phase susceptibility plots at zero applied field for **2** from 2 - 66 K.

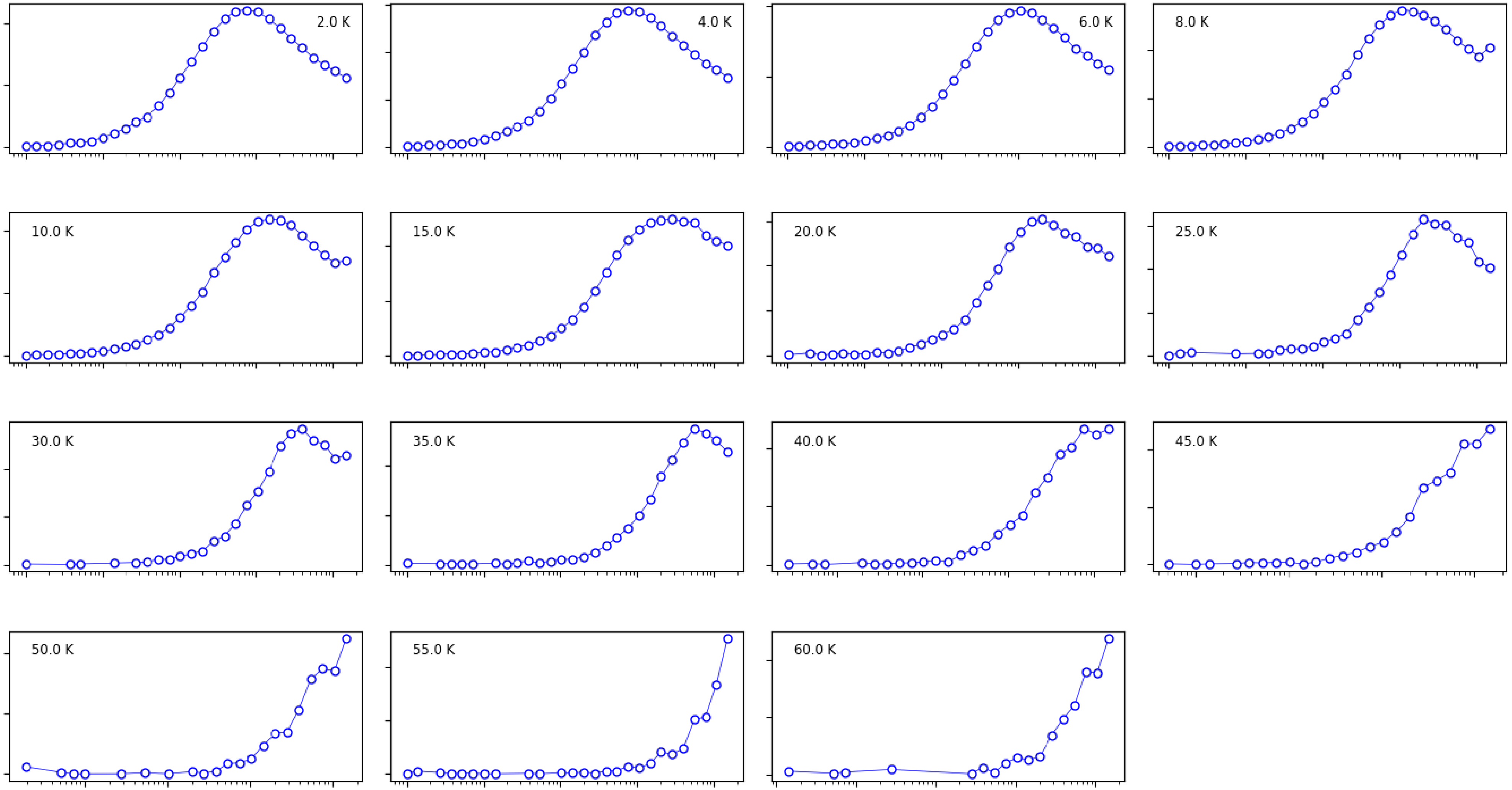

Figure S29. Out of phase susceptibility plots at zero applied field for **3** from 2 – 60 K.

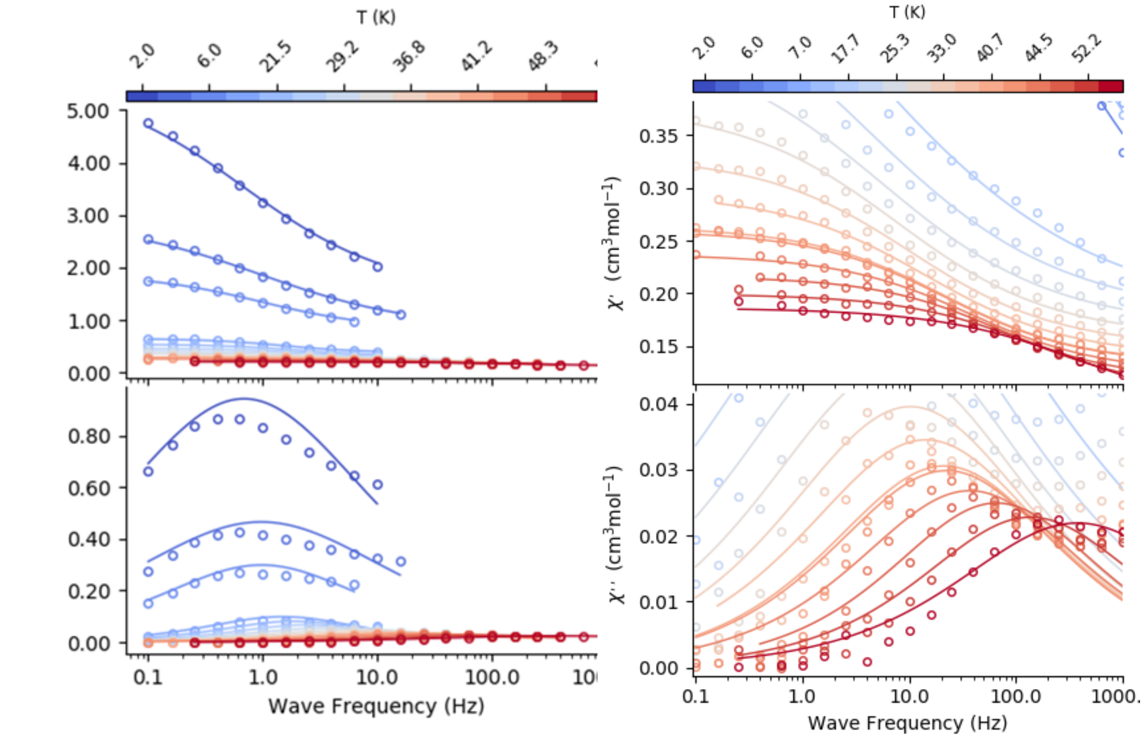

Figure S30. *Left*: ac frequency dependences between 2-8 K and 17-56 K for ac frequencies between 0.1 and 1000 Hz (in a zero-dc field) for **1**. Solid lines are the generalised Debye fit of the ac data used to extract the temperature dependence of the relaxation time. *Right*: Zoomed-in view of the left figure for visualising high temperature data.

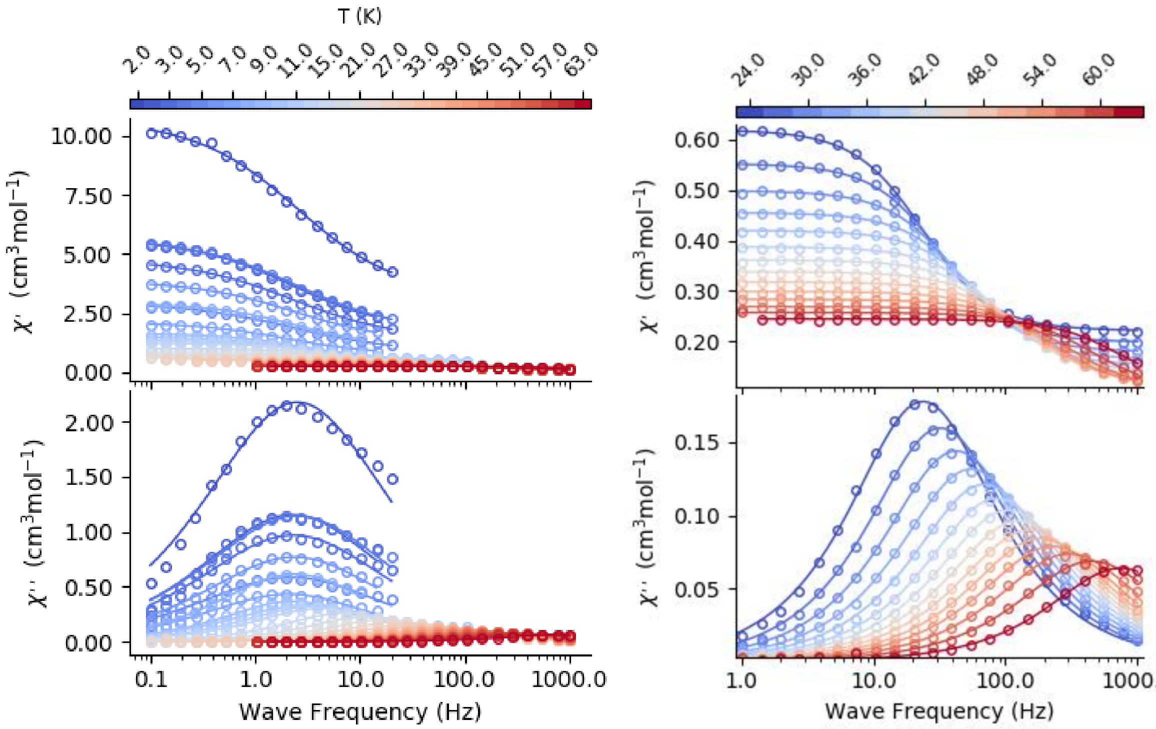

Figure S31. *Left*: ac frequency dependences between 2 and 63 K for **2**. The ac frequencies range between 0.1 and 1000 Hz for the temperature range 2-21 K and 0.1 and 1000 Hz for the temperature range 24-63 K, under zero dc field. Solid lines are the generalised Debye fit of the ac data used to extract the temperature dependence of the relaxation time. *Right*: Zoomed-in view of the left figure for visualising high temperature data (24 -63 K) lying in the frequency range 1-1000 Hz.

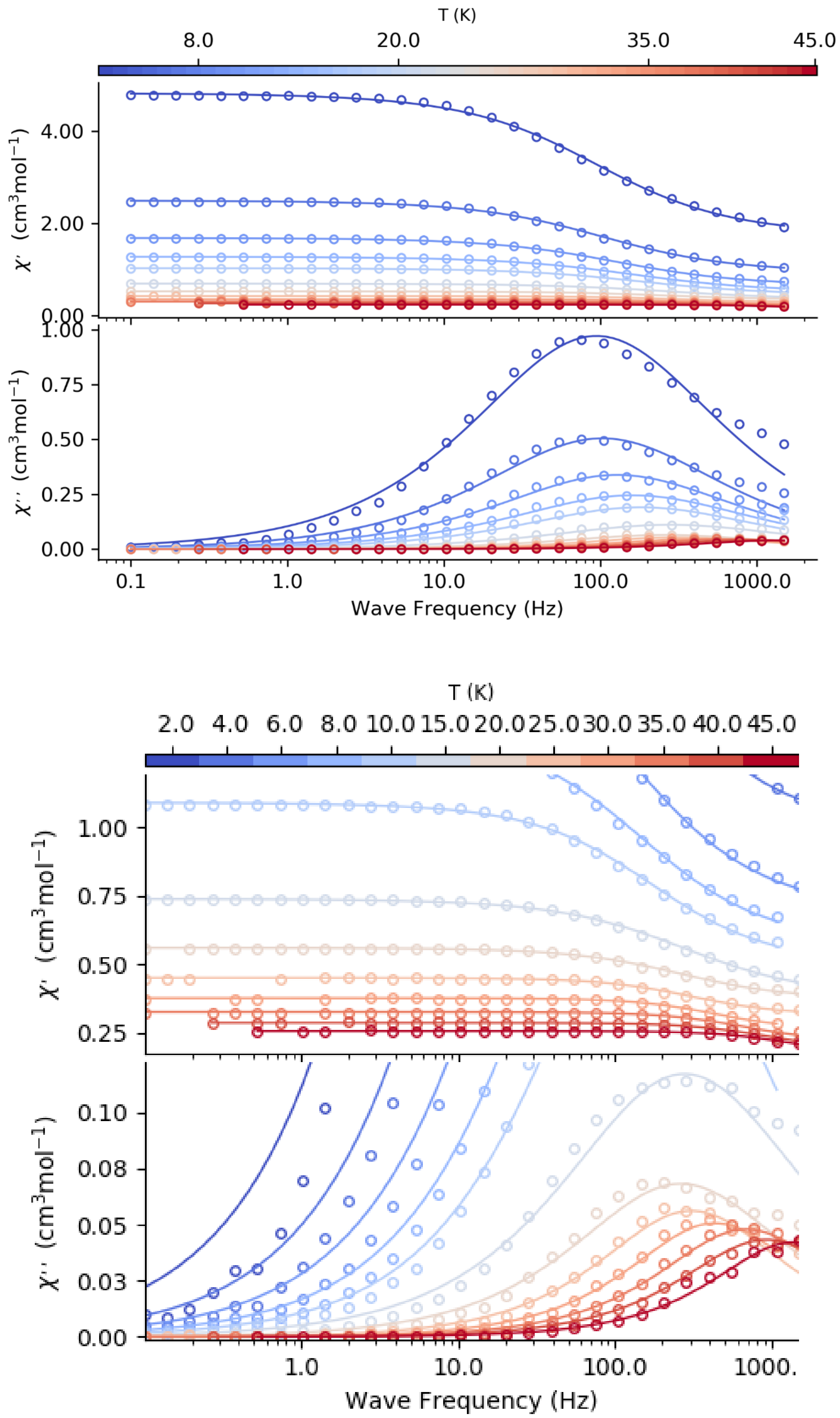

Figure S32. *Top*: ac frequency dependences between 2 and 45 K for ac frequencies between 0.1 and 1000 Hz (in a zero-dc field) for **3**. Solid lines are the generalised Debye fit of the ac data used to extract the temperature dependence of the relaxation time. *Bottom*: Zoomed-in view of the top figure for visualising high temperature data.

Magnetic relaxation times and their distribution from ac and dc data

Generalised Debye model:

$$\chi'(\omega) = \chi_s + (\chi_T - \chi_s)\frac{1 + (\omega\tau)^{1-\alpha}\sin\left(\frac{\pi\alpha}{2}\right)}{1 + 2(\omega\tau)^{1-\alpha}\sin\left(\frac{\pi\alpha}{2}\right) + (\omega\tau)^{2-2\alpha}} \quad (4)$$

$$\chi''(\omega) = (\chi_T - \chi_s)\frac{(\omega\tau)^{1-\alpha}\cos\left(\frac{\pi\alpha}{2}\right)}{1 + 2(\omega\tau)^{1-\alpha}\sin\left(\frac{\pi\alpha}{2}\right) + (\omega\tau)^{2-2\alpha}} \quad (5)$$

Where χ_s and χ_T are the adiabatic and isothermal susceptibilities, ω is the frequency, τ are the relaxation times and α represents the distribution of the relaxation times.

Extended Debye model for two processes representing two different relaxation channels operating simultaneously, is expressed as follows:

$$\chi'(\omega) = \chi_s + (\chi_T - \chi_s)\times\left[\frac{\beta\{1 + (\omega\tau_1)^{1-\alpha_1}\sin\left(\frac{\pi\alpha_1}{2}\right)\}}{1 + 2(\omega\tau_1)^{1-\alpha_1}\sin\left(\frac{\pi\alpha_1}{2}\right) + (\omega\tau_1)^{2-2\alpha_1}} + \frac{(1-\beta)\{1 + (\omega\tau_2)^{1-\alpha_2}\sin\left(\frac{\pi\alpha_2}{2}\right)\}}{1 + 2(\omega\tau_2)^{1-\alpha_2}\sin\left(\frac{\pi\alpha_2}{2}\right) + (\omega\tau_2)^{2-2\alpha_2}}\right] \quad (6)$$

$$\chi''(\omega) = (\chi_T - \chi_s)\times\left[\frac{\beta(\omega\tau_1)^{1-\alpha_1}\cos\left(\frac{\pi\alpha_1}{2}\right)}{1 + 2(\omega\tau_1)^{1-\alpha_1}\sin\left(\frac{\pi\alpha_1}{2}\right) + (\omega\tau_1)^{2-2\alpha_1}} + \frac{(1-\beta)(\omega\tau_2)^{1-\alpha_2}\cos\left(\frac{\pi\alpha_2}{2}\right)}{1 + 2(\omega\tau_2)^{1-\alpha_2}\sin\left(\frac{\pi\alpha_2}{2}\right) + (\omega\tau_2)^{2-2\alpha_2}}\right] \quad (7)$$

Where τ_1 and τ_2 are the relaxation times at a given temperature due to the two-independent process, α_1 and α_2 represent their distribution. β is the weight of the first relaxation, and $(1-\beta)$ is a similar τ_2 counterpart. Both in-phase and out of phase ac susceptibility components are fitted simultaneously to extract the relaxation parameters.

The CC-Fit2 program was used to fit the ac susceptibility data to extract the τ, α, χ_S, χ_T values and their associated errors; for details regarding the fitting procedure and uncertainty estimate, follow the procedures in reference 19.

DC

The time-dependent magnetic moment (magnetic decay curves) of a sample can be fitted using a stretched exponential function (Equation 8) in CC-FIT2.[19] The fitting parameters with their uncertainties for **5%Dy@1-Y** from 2-8 K are compiled in Table S15. The β parameter in the stretched exponential function relates to a distribution of τ and consequently to an intrinsic uncertainty on the value similar to α parameter in the ac data fiting. These uncertainties were calculated as described in the ESI of reference 20.

$$M(t) = M_{eq} + (M_0 - M_{eq})e^{(-t/\tau)^{\beta}} \quad (8)$$

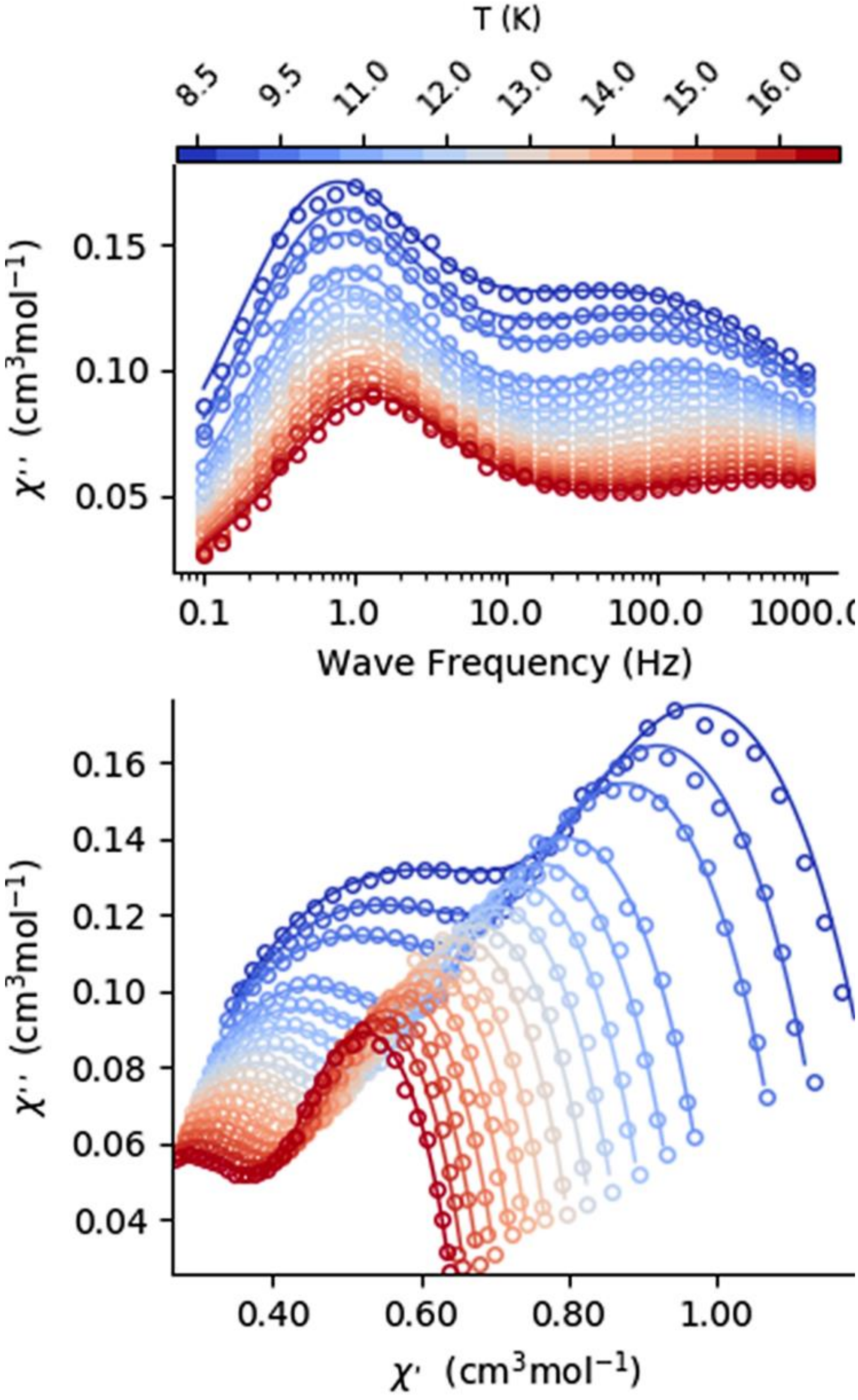

Figure S33. Temperature and frequency dependence of the out of phase molar magnetic susceptibility (top) and Cole-Cole plots (bottom) for **1** from 8–16.5 K. Solid lines represent the best fit using two processes.

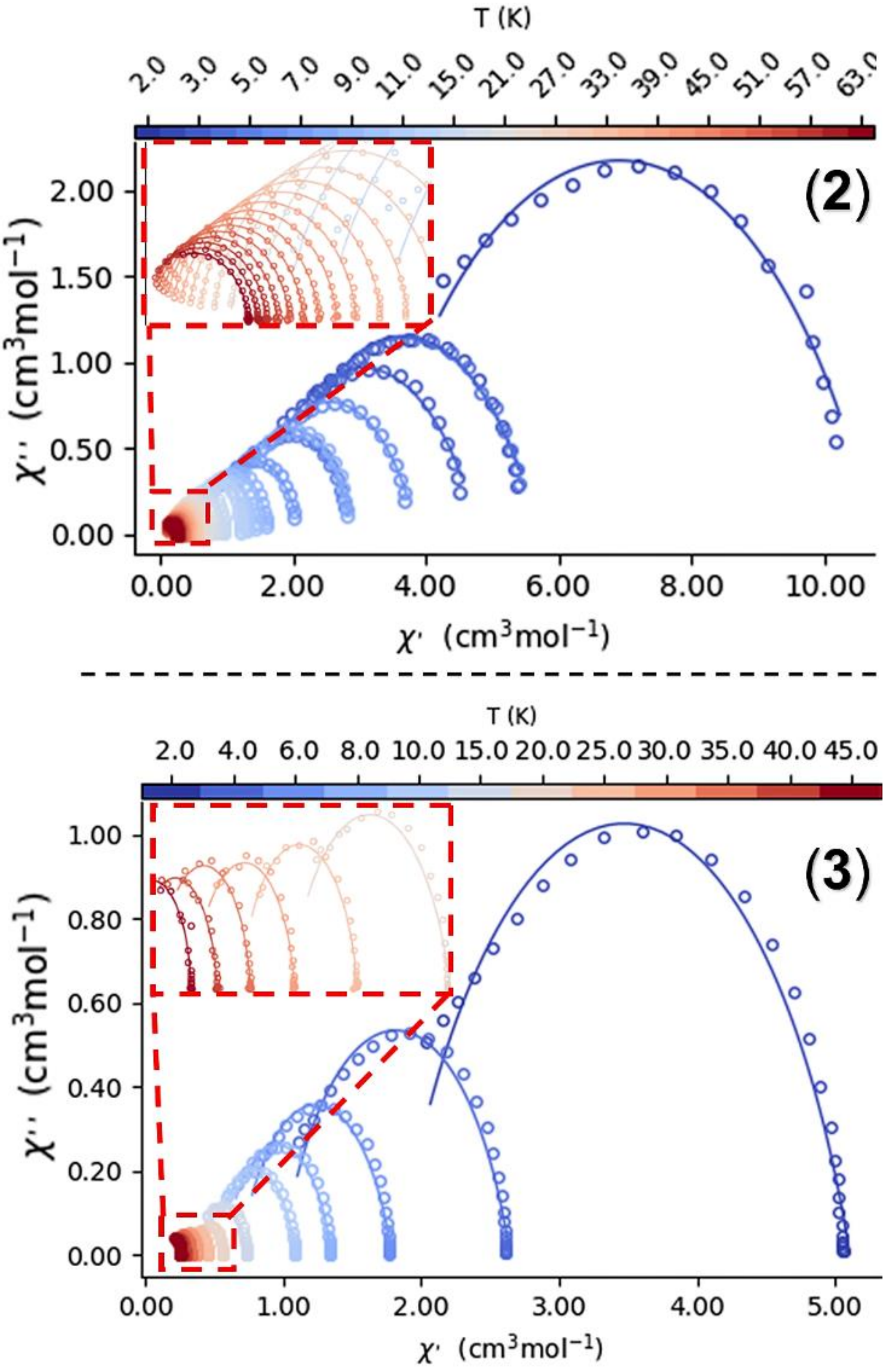

Figure S34. Cole-Cole plots from 2–63 K for **2** and 2–45 K for **3**. Solid lines represent the best fit using the generalised Debye model.

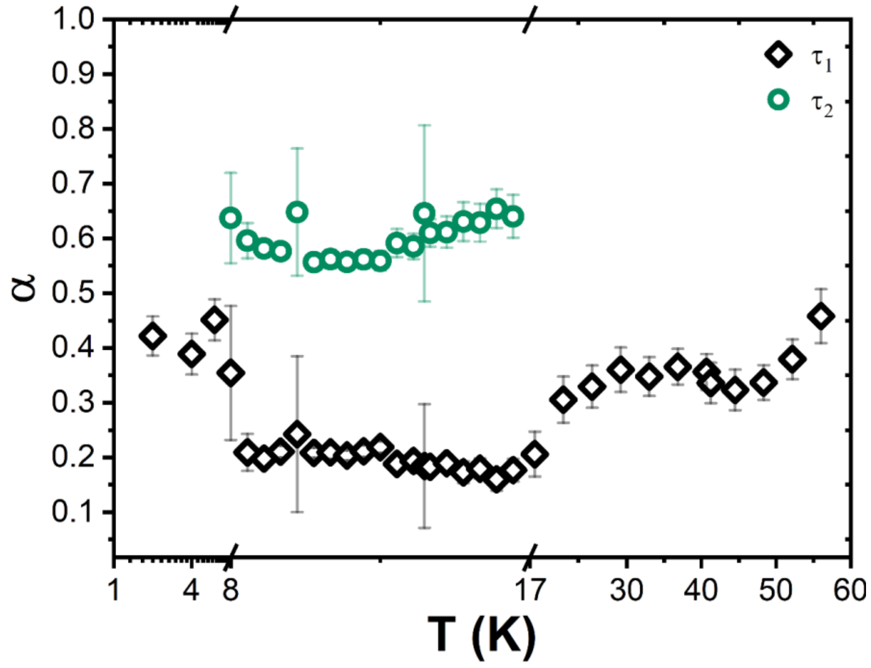

Figure S35. The relaxation parameter, α that represents the distribution of the relaxation time, against temperature for **1** as extracted from the generalised Debye fitting (2-8 K and 17-56 K) and extended Debye fitting (8-17 K) of the ac susceptibility data. Green circles show the α_2 parameter in the temperature range 8-17 K denoting the distribution about τ_2 of the secondary relaxation channel in **1**.

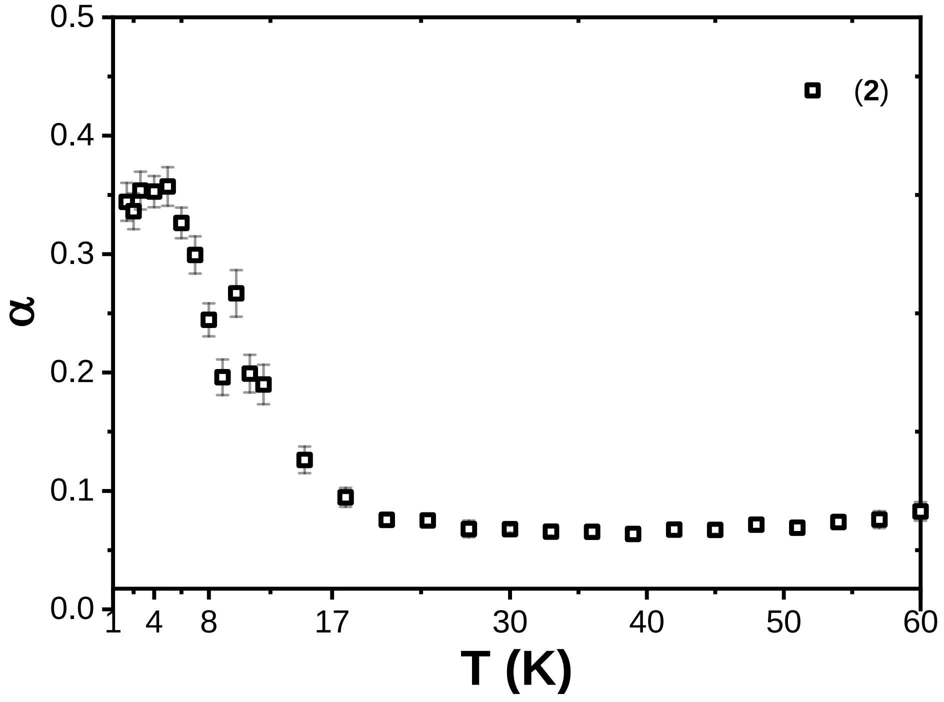

Figure S36. The relaxation parameter α with varying temperature from 2-63 K for **2** as extracted from the fitting of zero-field ac susceptibility data using the generalised Debye model.

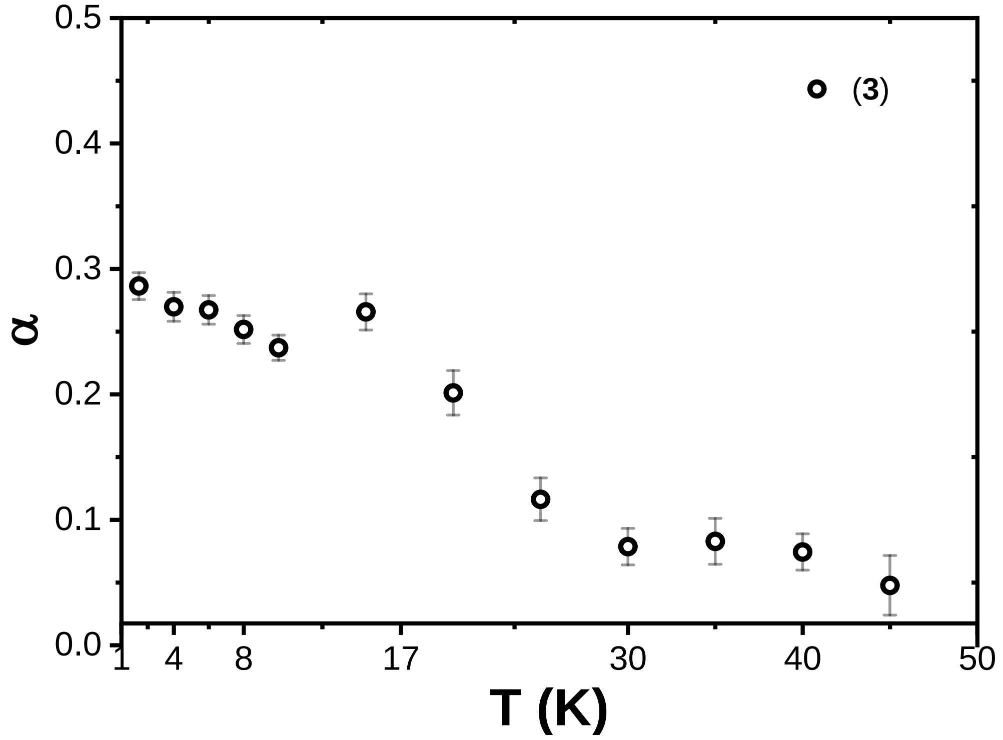

Figure S37. The relaxation parameter α with varying temperature from 2-45 K for **3** as extracted from the fitting of zero-field ac susceptibility data using the generalised Debye model.

Table S10. The fitting parameters of the Cole-Cole isotherms for **1** from 2-6 and 17-56 K under zero dc field using CC-FIT2.

T	τ (s)	τ (error)	χ_S	χ_T	α	α (error)
2	2.47E-01	2.30E-02	1.70E+00	5.71E+00	0.42	3.58E-02
4	1.95E-01	1.75E-02	1.05E+00	2.92E+00	0.39	3.73E-02
6	1.30E-01	1.44E-02	6.84E-01	2.02E+00	0.45	3.77E-02
17.67	1.06E-01	8.29E-03	3.90E-01	6.75E-01	0.21	4.11E-02
21.5	6.61E-02	6.61E-03	2.97E-01	5.68E-01	0.31	4.23E-02
25.33	4.58E-02	4.50E-03	2.53E-01	4.86E-01	0.33	3.85E-02
29.17	2.99E-02	3.42E-03	2.17E-01	4.25E-01	0.36	4.07E-02
33	2.20E-02	2.18E-03	1.99E-01	3.75E-01	0.35	3.54E-02
36.83	1.45E-02	1.37E-03	1.80E-01	3.36E-01	0.37	3.27E-02
40.67	9.87E-03	9.89E-04	1.68E-01	3.03E-01	0.36	3.29E-02
41.25	1.00E-02	1.11E-03	1.70E-01	2.98E-01	0.34	3.69E-02
44.5	6.10E-03	6.89E-04	1.60E-01	2.72E-01	0.32	3.72E-02
48.33	3.03E-03	2.97E-04	1.46E-01	2.49E-01	0.34	3.19E-02
52.17	1.40E-03	2.23E-04	1.30E-01	2.31E-01	0.38	3.65E-02
56	4.31E-04	1.62E-04	1.03E-01	2.16E-01	0.46	4.91E-02

Table S11. The fitting parameters of the extended Debye fitting for **1** from 8-17 K under zero dc field using CC-FIT2.

T	τ_1 (s)	τ_1 (error)	α_1	α_1 (error)	τ_2 (s)	τ_2 (error)	α_2	α_2 (error)
8	2.40E-01	1.70E-02	0.09	1.22E-01	3.14E-03	2.11E-03	0.64	8.25E-02
8.5	2.72E-01	1.03E-02	0.21	3.39E-02	1.70E-03	2.71E-04	0.60	3.23E-02
9	2.48E-01	4.50E-03	0.20	1.51E-02	1.39E-03	8.78E-05	0.58	1.58E-02
9.5	2.38E-01	3.34E-03	0.21	1.07E-02	1.15E-03	5.06E-05	0.58	1.23E-02
10	1.91E-01	1.66E-02	0.05	1.42E-01	1.40E-03	8.22E-04	0.65	1.16E-01
10.5	2.14E-01	2.74E-03	0.21	8.81E-03	8.62E-04	3.03E-05	0.56	1.18E-02
11	2.08E-01	3.68E-03	0.21	1.23E-02	7.64E-04	3.89E-05	0.56	1.72E-02
11.5	2.00E-01	2.62E-03	0.20	8.99E-03	6.81E-04	2.69E-05	0.56	1.33E-02
12	1.91E-01	2.96E-03	0.21	1.05E-02	5.89E-04	2.99E-05	0.56	1.66E-02
12.5	1.84E-01	2.99E-03	0.22	1.06E-02	5.13E-04	2.94E-05	0.56	1.79E-02
13	1.83E-01	3.49E-03	0.19	1.57E-02	4.76E-04	4.41E-05	0.59	2.57E-02
13.5	1.71E-01	2.92E-03	0.19	1.33E-02	4.24E-04	3.70E-05	0.59	2.33E-02
13.83	1.51E-01	1.17E-02	0.10	1.13E-01	4.04E-04	3.05E-04	0.65	1.61E-01
14	1.66E-01	2.57E-03	0.18	1.44E-02	3.72E-04	3.97E-05	0.61	2.52E-02
14.5	1.60E-01	2.73E-03	0.19	1.56E-02	3.35E-04	4.33E-05	0.61	2.88E-02
15	1.56E-01	2.78E-03	0.17	1.99E-02	3.06E-04	5.44E-05	0.63	3.55E-02
15.5	1.46E-01	2.47E-03	0.18	1.85E-02	2.87E-04	5.00E-05	0.63	3.45E-02
16	1.44E-01	2.12E-03	0.16	2.08E-02	2.68E-04	5.37E-05	0.65	3.54E-02
16.5	1.37E-01	2.29E-03	0.18	2.06E-02	2.47E-04	5.46E-05	0.64	3.94E-02

Table S12. The fitting parameters of the Cole-Cole isotherms for **2** from 2-63 K under zero dc field using CC-FIT2.

T	τ (s)	τ (error)	χ_S	χ_T	α	α (error)
2.0	6.28E-02	2.59E-03	3.07E+00	1.08E+01	0.34	1.60E-02
2.5	6.99E-02	2.71E-03	1.65E+00	5.67E+00	0.34	1.52E-02
3.0	6.87E-02	2.83E-03	1.32E+00	4.80E+00	0.35	1.60E-02
4.0	6.35E-02	2.19E-03	1.61E+00	5.71E+00	0.35	1.32E-02
5.0	7.17E-02	3.01E-03	8.36E-01	2.95E+00	0.36	1.63E-02
6.0	6.73E-02	2.21E-03	1.24E+00	3.88E+00	0.33	1.30E-02
7.0	7.66E-02	2.80E-03	7.07E-01	2.12E+00	0.30	1.58E-02
8.0	6.69E-02	2.09E-03	1.09E+00	2.91E+00	0.24	1.39E-02
9.0	6.92E-02	2.10E-03	6.31E-01	1.63E+00	0.20	1.51E-02
10.0	4.94E-02	2.23E-03	4.75E-01	1.50E+00	0.27	1.97E-02
11.0	4.46E-02	1.42E-03	4.70E-01	1.35E+00	0.20	1.59E-02
12.0	3.66E-02	1.23E-03	4.20E-01	1.24E+00	0.19	1.67E-02
15.0	2.23E-02	4.62E-04	3.49E-01	9.85E-01	0.13	1.12E-02
18.0	1.41E-02	2.00E-04	2.93E-01	8.22E-01	0.09	8.00E-03
21.0	9.58E-03	9.85E-05	2.55E-01	7.06E-01	0.08	5.91E-03
24.0	6.71E-03	4.45E-05	2.20E-01	6.20E-01	0.08	3.71E-03
27.0	4.95E-03	6.15E-05	1.98E-01	5.53E-01	0.07	7.08E-03
30.0	3.75E-03	1.92E-05	1.79E-01	4.99E-01	0.07	2.95E-03
33.0	2.91E-03	1.69E-05	1.64E-01	4.55E-01	0.07	3.47E-03
36.0	2.30E-03	1.42E-05	1.52E-01	4.20E-01	0.07	3.68E-03
39.0	1.85E-03	1.25E-05	1.41E-01	3.87E-01	0.06	4.06E-03
42.0	1.51E-03	1.20E-05	1.31E-01	3.61E-01	0.07	4.70E-03
45.0	1.26E-03	7.46E-06	1.24E-01	3.38E-01	0.07	3.49E-03
48.0	1.04E-03	6.81E-06	1.17E-01	3.18E-01	0.07	3.78E-03
51.0	8.63E-04	8.59E-06	1.11E-01	2.99E-01	0.07	5.72E-03
54.0	6.97E-04	7.31E-06	1.06E-01	2.83E-01	0.07	5.83E-03
57.0	5.23E-04	7.14E-06	1.03E-01	2.69E-01	0.08	7.18E-03
60.0	3.54E-04	5.98E-06	1.01E-01	2.57E-01	0.08	7.86E-03
63.0	2.15E-04	5.96E-06	1.03E-01	2.45E-01	0.07	1.05E-02

Table S13. The fitting parameters of Cole-Cole isotherms for **3** from 2-45 K under zero dc field using CC-FIT2.

T	τ (s)	τ (error)	χ_S	χ_T	α	α (error)
2	1.69E-03	5.05E-05	1.84E+00	5.11E+00	0.29	1.07E-02
4	1.55E-03	4.81E-05	9.88E-01	2.64E+00	0.27	1.15E-02
6	1.25E-03	3.88E-05	6.86E-01	1.79E+00	0.27	1.14E-02
8	1.07E-03	3.30E-05	5.68E-01	1.35E+00	0.25	1.11E-02
10	9.33E-04	2.56E-05	4.95E-01	1.09E+00	0.24	1.01E-02
15	5.75E-04	2.56E-05	3.79E-01	7.39E-01	0.27	1.44E-02
20	6.09E-04	2.72E-05	3.72E-01	5.61E-01	0.20	1.78E-02
25	5.20E-04	1.80E-05	3.16E-01	4.51E-01	0.12	1.70E-02
30	3.69E-04	1.14E-05	2.63E-01	3.78E-01	0.08	1.46E-02
35	2.33E-04	9.83E-06	2.18E-01	3.27E-01	0.08	1.83E-02
40	1.73E-04	6.36E-06	1.91E-01	2.88E-01	0.07	1.45E-02
45	1.15E-04	8.21E-06	1.67E-01	2.58E-01	0.05	2.37E-02
2	1.69E-03	5.05E-05	1.84E+00	5.11E+00	0.29	1.07E-02
4	1.55E-03	4.81E-05	9.88E-01	2.64E+00	0.27	1.15E-02
6	1.25E-03	3.88E-05	6.86E-01	1.79E+00	0.27	1.14E-02
8	1.07E-03	3.30E-05	5.68E-01	1.35E+00	0.25	1.11E-02
10	9.33E-04	2.56E-05	4.95E-01	1.09E+00	0.24	1.01E-02
15	5.75E-04	2.56E-05	3.79E-01	7.39E-01	0.27	1.44E-02
20	6.09E-04	2.72E-05	3.72E-01	5.61E-01	0.20	1.78E-02
25	5.20E-04	1.80E-05	3.16E-01	4.51E-01	0.12	1.70E-02
30	3.69E-04	1.14E-05	2.63E-01	3.78E-01	0.08	1.46E-02
35	2.33E-04	9.83E-06	2.18E-01	3.27E-01	0.08	1.83E-02
40	1.73E-04	6.36E-06	1.91E-01	2.88E-01	0.07	1.45E-02
45	1.15E-04	8.21E-06	1.67E-01	2.58E-01	0.05	2.37E-02

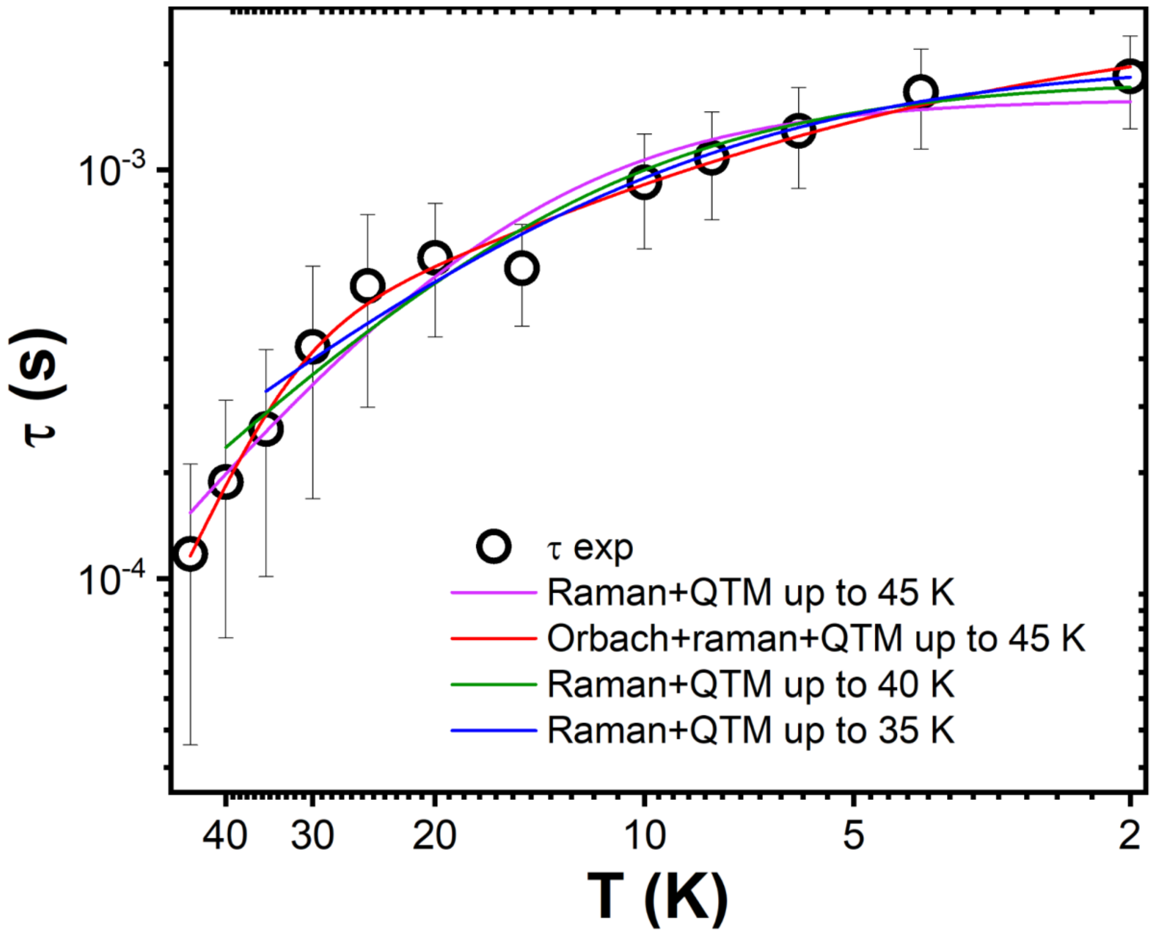

Figure S38. Fitting of the observed temperature dependent relaxation times for **3** between 2-45 K with and without including the Orbach process. The extracted parameters are tabulated below.

Table S14. U_{eff}, τ_0, C, *n* and τ_{QTM} parameters generated from the fit of the relaxation time-temperature dependence (Figure 5) for **3**. These parameters are given with their one-sigma ESDs (±), subscripts and superscripts.

	Orbach +Raman + QTM (2-45 K)	Raman + QTM (2-45 K)	Raman + QTM (2-40 K)	Raman + QTM (2-35 K)
**τ_0 (10^{-6} s)**	$1.24^{+3.74}_{-0.92}$	-	-	-
**U_{eff} (K)**	224 ± 62	-	-	-
**C (s^{-1} K^{-n})**	75^{+117}_{-45}	$2.3^{+4.8}_{-1.5}$	$8.1^{+13.6}_{-5.0}$	20^{+29}_{-12}
n	0.95 ± 0.29	2.08 ± 0.32	1.69 ± 0.28	1.40 ± 0.27
**τ_{QTM} (10^{-3} s)**	$2.41^{+1.04}_{-0.73}$	$1.49^{+0.21}_{-0.18}$	$1.66^{+0.28}_{-0.24}$	$1.85^{+0.39}_{-0.32}$

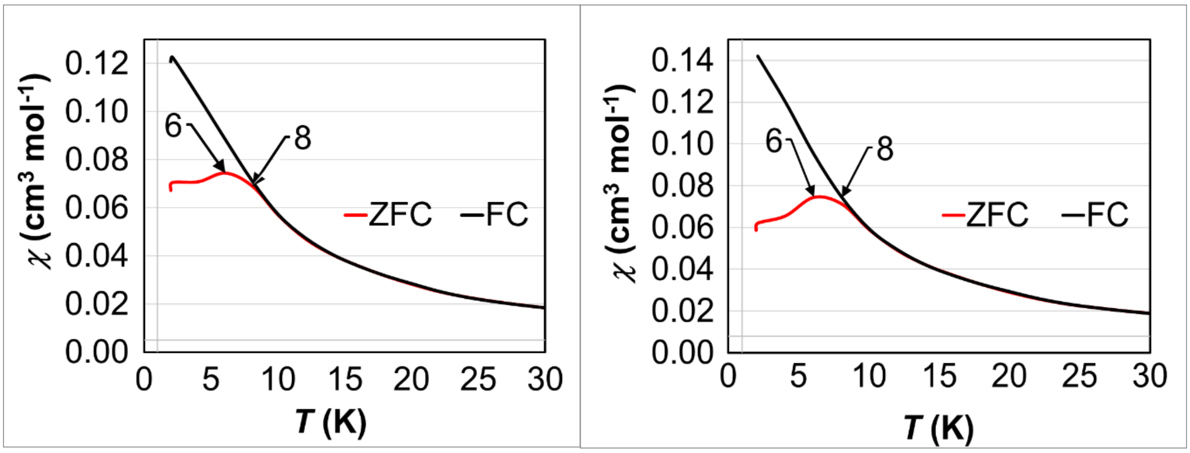

Figure S39. Zero field cooled (ZFC) – Field cooled (FC) plot for **5%Dy@1-Y** showing the temperature dependence of χ at 500 Oe (*left*) and 1000 Oe (*right*) in FC and ZFC modes. The arrows are showing T_{B}^{ZFC} at 6 K and $T_{\mathrm{irreversible}}$ at 8 K.

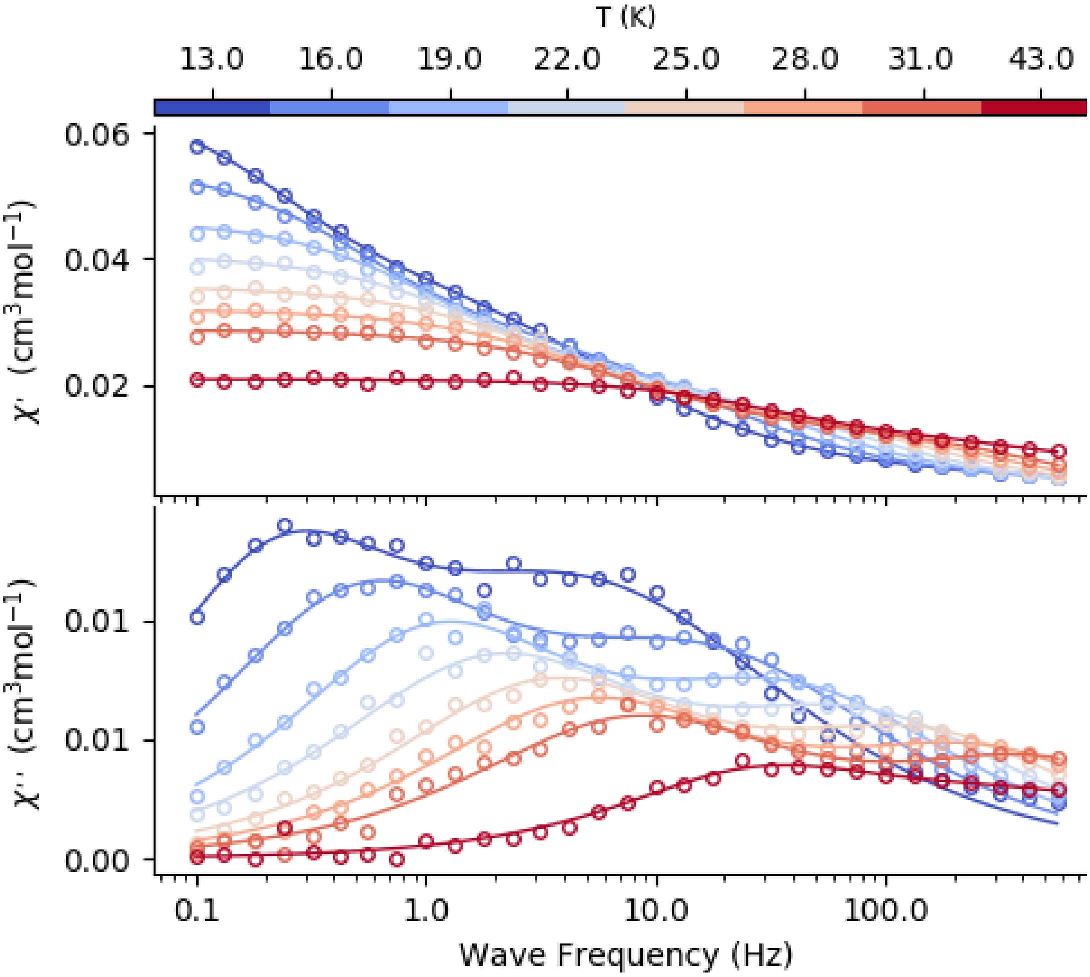

Figure S40. Temperature and frequency dependence of the out of phase molar magnetic susceptibility for **5%Dy@1-Y** from 13–43 K. Solid lines represent best fit using two processes.

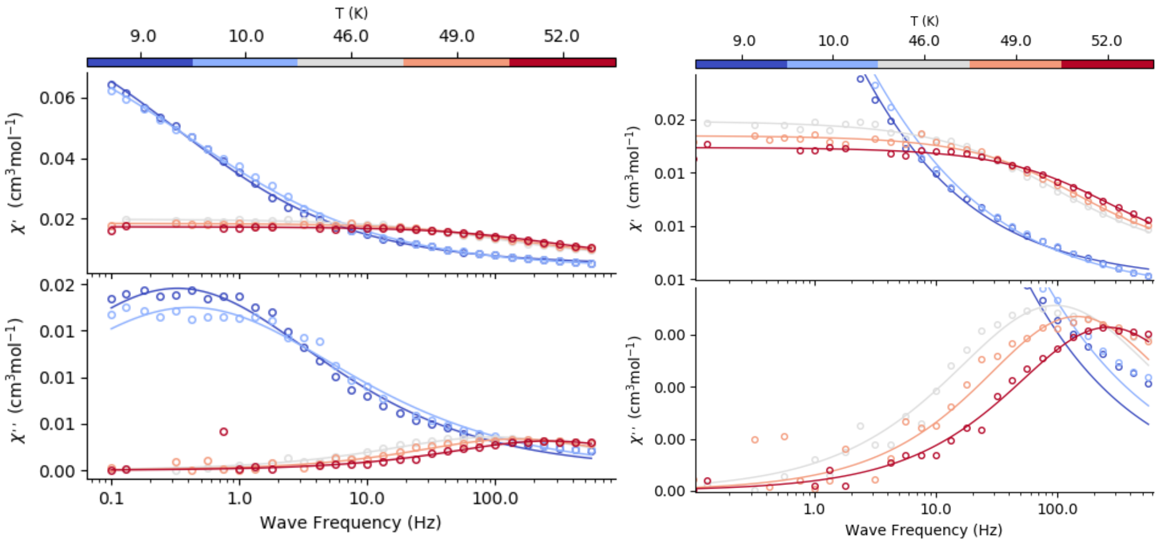

Figure S41. *Left*: ac frequency dependences for temperatures 9, 10 and 46-52 K for ac frequencies between 0.1 and 1000 Hz (in a zero-dc field) for **5%Dy@1-Y**. Solid lines are the generalised Debye fit of the ac data used to extract the temperature dependence of the relaxation time. *Right*: Zoomed-in view of the left figure for visualising high temperature data lying in the frequency range 10-1000 Hz.

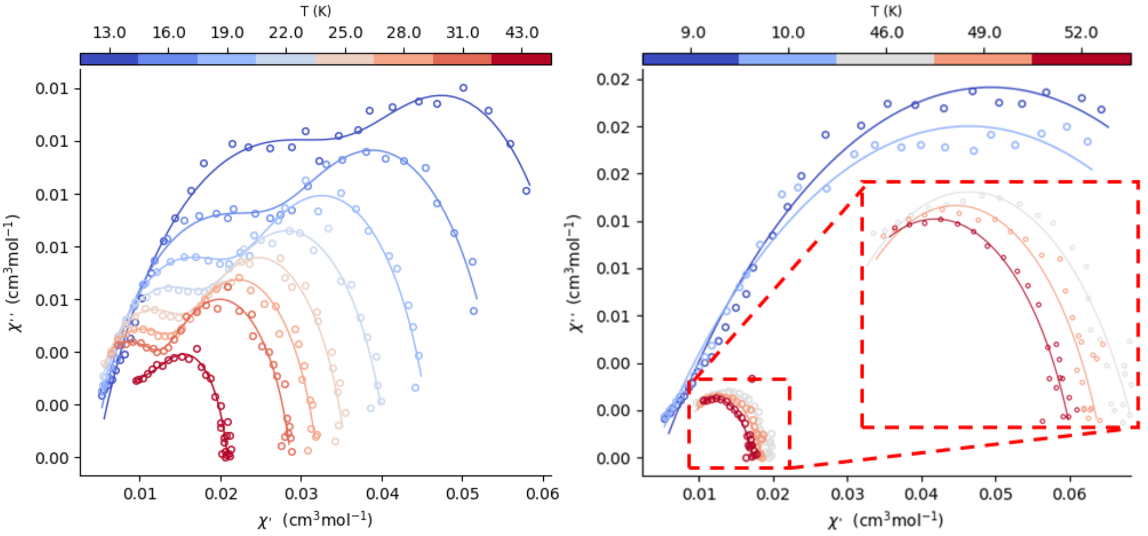

Figure S42. *Left*: The fitting of the Cole-Cole isotherms for **5%Dy@1-Y** 13-43 K via extended Debye model, and from 9, 10, 46-52 K via generalised Debye model and, under zero dc field using CC-FIT2.

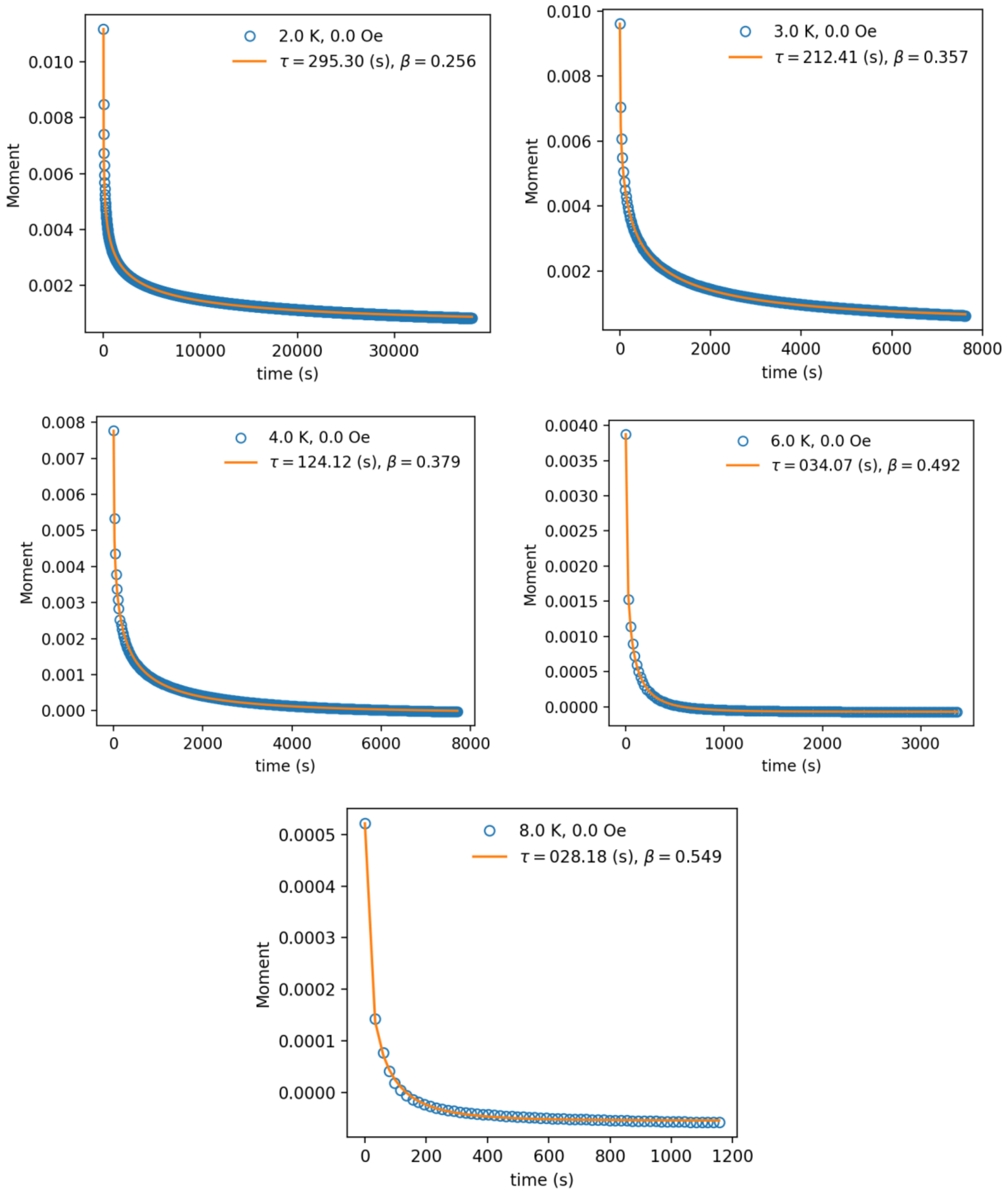

Figure S43. Magnetic relaxation (dc decays) for **5%Dy@1-Y** at 2, 3, 4, 6 and 8 K. Blue circles represent the observed data and solid orange lines represent best fit using the stretched exponential function in CC-FIT2.

Table S15. The fitting parameters of DC magnetic decays for **5%Dy@1-Y** from 2-8 K using the stretched exponential function in CC-FIT2.

T	M_0	M_{eq}	τ (s)	τ (error)	ß	ß (error)
2	1.12E-02	5.41E-04	2.95E+02	2.00E+00	2.56E-01	1.17E-03
3	9.62E-03	4.17E-04	2.12E+02	1.59E+00	3.57E-01	2.11E-03
4	7.76E-03	-6.72E-05	1.24E+02	1.21E+00	3.79E-01	2.65E-03
6	3.88E-03	-7.16E-05	3.41E+01	2.20E-01	4.92E-01	2.18E-03
8	5.22E-04	-5.46E-05	2.82E+01	6.02E-01	5.49E-01	9.73E-03

Table S16. The fitting parameters of the Cole-Cole isotherms for **5%Dy@1-Y** from 9, 10, 46-52 K via generalised Debye model and 13-43 K via extended Debye model, under zero dc field using the CC-FIT2.

T	τ_1 (s)	τ_1 (error)	α_1	α_1 (error)	τ_2 (s)	τ_2 (error)	α_2	α_2 (error)
13	7.23E-01	3.50E-02	0.10	4.34E-02	2.81E-02	3.04E-03	0.34	1.82E-02
16	3.12E-01	1.36E-02	0.18	2.37E-02	8.67E-03	7.21E-04	0.30	2.11E-02
19	1.49E-01	6.32E-03	0.18	1.92E-02	3.79E-03	2.66E-04	0.25	2.58E-02
22	8.32E-02	4.63E-03	0.22	2.05E-02	1.86E-03	1.46E-04	0.22	3.90E-02
25	4.76E-02	3.18E-03	0.21	2.28E-02	1.02E-03	9.03E-05	0.20	5.79E-02
28	3.17E-02	1.81E-03	0.22	1.81E-02	5.84E-04	5.13E-05	0.21	6.31E-02
31	2.00E-02	1.61E-03	0.23	2.34E-02	3.25E-04	6.93E-05	0.20	1.18E-01
43	6.03E-03	1.26E-03	0.13	1.33E-01	3.12E-04	3.01E-04	0.40	1.99E-01

T	τ (s)	τ (error)	χ_S	χ_T	α	α (error)
9	4.83E-01	3.07E-02	4.75E-03	9.41E-02	4.74E-01	8.97E-03
10	3.82E-01	2.88E-02	3.63E-03	8.93E-02	5.06E-01	1.03E-02
46	1.65E-03	1.12E-04	7.07E-03	1.98E-02	3.52E-01	1.87E-02
49	1.10E-03	1.10E-04	7.41E-03	1.85E-02	3.06E-01	2.79E-02
52	6.06E-04	1.99E-04	6.93E-03	1.74E-02	3.09E-01	7.57E-02

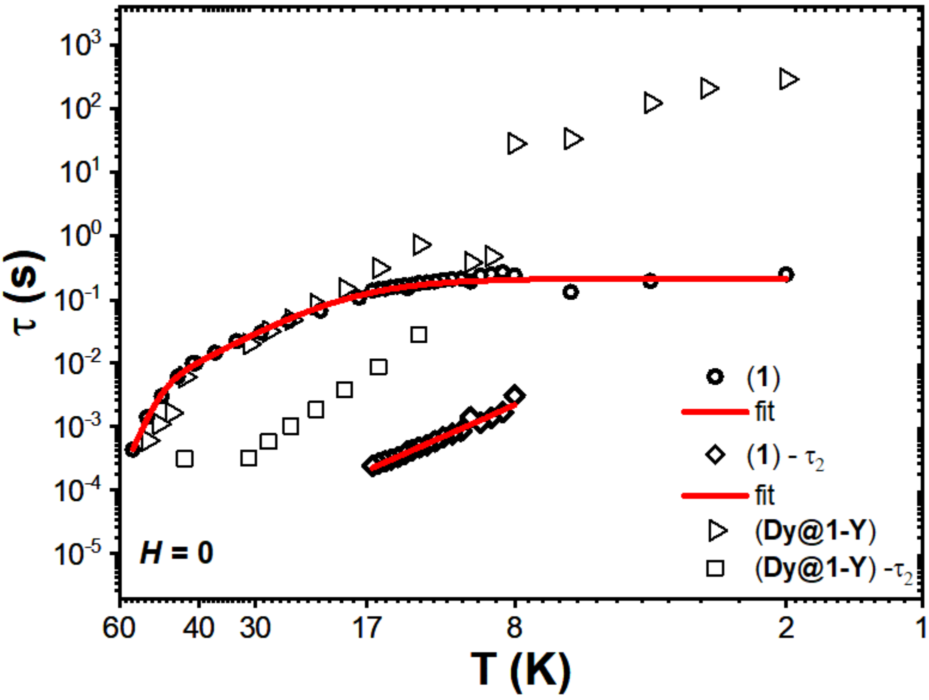

Figure S44. Temperature dependence of the relaxation time from 2 - 56 K for **1** (circles and diamonds) and **5%Dy@1-Y** (Triangles).

6. References

1 T. Watanabe, Y. Ishida, T. Matsuo and H. Kawaguchi, *Dalton Trans.*, 2010, **39**, 484–491.

2 V. S. Parmar, F. Ortu, X. Ma, N. F. Chilton, R. Clérac, D. P. Mills and R. E. P. Winpenny, *Chem. Eur. J.*, 2020, **26**, 7774–7778.

3 C. J. Hoerger, H. S. La Pierre, L. Maron, A. Scheurer, F. W. Heinemann and K. Meyer, *Chem. Commun.*, 2016, **52**, 10854–10857.

4 S. A. Moehring, M. Miehlich, C. J. Hoerger, K. Meyer, J. W. Ziller and W. J. Evans, *Inorg. Chem.*, 2020, **59**, 3207–3214.

5 Agilent, *Technol. UK Ltd, Yarnton, Oxford, UK*, 2014, **44**, 1–53.

6 G. M. Sheldrick, *Acta Crystallogr. Sect. A Found. Crystallogr.*, 2008, **64**, 112–122.

7 G. M. Sheldrick, *Acta Crystallogr. Sect. C Struct. Chem.*, 2015, **71**, 3–8.

8 O. V. Dolomanov, L. J. Bourhis, R. J. Gildea, J. A. K. Howard and H. Puschmann, *J. Appl. Crystallogr.*, 2009, **42**, 339–341.

9 L. J. Farrugia, *J. Appl. Crystallogr.*, 2012, **45**, 849–854.

10 Persistence of Vision Raytracer (Version 3.6), 2004.

11 S. Alvarez, P. Alemany, D. Casanova, J. Cirera, M. Llunell and D. Avnir, *Coord. Chem. Rev.*, 2005, **249**, 1693–1708.

12 F. Aquilante, J. Autschbach, R. K. Carlson, L. F. Chibotaru, M. G. Delcey, L. De Vico, I. Fdez. Galván, N. Ferré, L. M. Frutos, L. Gagliardi, M. Garavelli, A. Giussani, C. E. Hoyer, G. Li Manni, H. Lischka, D. Ma, P. Å. Malmqvist, T. Müller, A. Nenov, M. Olivucci, T. B. Pedersen, D. Peng, F. Plasser, B. Pritchard, M. Reiher, I. Rivalta, I. Schapiro, J. Segarra-Martí, M. Stenrup, D. G. Truhlar, L. Ungur, A. Valentini, S. Vancoillie, V. Veryazov, V. P. Vysotskiy, O. Weingart, F.

Zapata and R. Lindh, *J. Comput. Chem.*, 2016, **37**, 506–541.

13 I. Fdez. Galván, M. Vacher, A. Alavi, C. Angeli, F. Aquilante, J. Autschbach, J. J. Bao, S. I. Bokarev, N. A. Bogdanov, R. K. Carlson, L. F. Chibotaru, J. Creutzberg, N. Dattani, M. G. Delcey, S. S. Dong, A. Dreuw, L. Freitag, L. M. Frutos, L. Gagliardi, F. Gendron, A. Giussani, L. González, G. Grell, M. Guo, C. E. Hoyer, M. Johansson, S. Keller, S. Knecht, G. Kovačević, E. Källman, G. Li Manni, M. Lundberg, Y. Ma, S. Mai, J. P. Malhado, P. Å. Malmqvist, P. Marquetand, S. A. Mewes, J. Norell, M. Olivucci, M. Oppel, Q. M. Phung, K. Pierloot, F. Plasser, M. Reiher, A. M. Sand, I. Schapiro, P. Sharma, C. J. Stein, L. K. Sørensen, D. G. Truhlar, M. Ugandi, L. Ungur, A. Valentini, S. Vancoillie, V. Veryazov, O. Weser, T. A. Wesołowski, P.-O. Widmark, S. Wouters, A. Zech, J. P. Zobel and R. Lindh, *J. Chem. Theory Comput.*, 2019, **15**, 5925–5964.

14 B. O. Roos, R. Lindh, P. Å. Malmqvist, V. Veryazov and P. O. Widmark, *J. Phys. Chem. A*, 2004, **108**, 2851–2858.

15 B. O. Roos, R. Lindh, P. Å. Malmqvist, V. Veryazov and P. O. Widmark, *J. Phys. Chem. A*, 2005, **109**, 6575–6579.

16 L. Ungur and L. F. Chibotaru, *Chem. Eur. J.*, 2017, **23**, 3708–3718.

17 S. K. Sur, *J. Magn. Reson.*, 1989, **82**, 169–173.

18 B. Yin and C. C. Li, *Phys. Chem. Chem. Phys.*, 2020, **22**, 9923–9933.

19 D. Reta and N. F. Chilton, *Phys. Chem. Chem. Phys.*, 2019, **21**, 23567–23575.

20 P. Evans, D. Reta, G. F. S. Whitehead, N. F. Chilton and D. P. Mills, *J. Am. Chem. Soc.*, 2019, **141**, 19935–19940.
